# Impact of the Histidine-Triazole and Tryptophan-Pyrene Exchange in the WHW Peptide: Cu(II) Binding, DNA/RNA Interactions and Bioactivity

**DOI:** 10.3390/ijms23137006

**Published:** 2022-06-23

**Authors:** Ivona Krošl, Marta Košćak, Karla Ribičić, Biserka Žinić, Dragomira Majhen, Ksenija Božinović, Ivo Piantanida

**Affiliations:** 1 Division of Organic Chemistry and Biochemistry, Ruđer Bošković Institute, Bijenička Cesta 54, 10000 Zagreb, Croatia; ivona.krosl@irb.hr (I.K.); marta.koscak@irb.hr (M.K.); karla.ribica@gmail.com (K.R.); biserka.zinic@irb.hr (B.Ž.); 2 Division of Molecular Biology, Ruđer Bošković Institute, Bijenička Cesta 54, 10000 Zagreb, Croatia; dragomira.majhen@irb.hr (D.M.); ksenija.bozinovic@irb.hr (K.B.)

**Keywords:** WHW peptidoid, pyrene, DNA and RNA binding, cell uptake, cytotoxicity

## Abstract

In three novel peptidoids based on the tryptophan—histidine—tryptophan (WHW) peptide, the central histidine was replaced by Ala-(triazole), and two derivatives also had one tryptophan replaced with pyrene-alkyls of different lengths and flexibility. Pyrene analogues show strong fluorescence at 480–500 nm, attributed to intramolecular exciplex formation with tryptophan. All three peptidoids bind Cu^2+^ cation in water with strong affinity, with Trp- Ala-(triazole)-Trp binding comparably to the parent WHW, and the pyrene analogues even stronger, demonstrating that replacement of histidine with triazole in peptides does not hamper Cu^2+^ coordination. The studied peptidoids strongly bind to ds-DNA and ds-RNA, whereby their complexes with Cu^2+^ exhibit distinctively different interactions in comparison to metal-free analogues, particularly in the stabilization of ds-DNA against thermal denaturation. The pyrene peptidoids efficiently enter living cells with no apparent cytotoxic effect, whereby their red-shifted emission compared to the parent pyrene allows intracellular confocal microscopy imaging, showing accumulation in cytoplasmic organelles. However, irradiation with 350 nm light resulted in evident antiproliferative effect on cells treated with micromolar concentrations of the pyrene analogues, presumably attributed to pyrene-induced production of singlet oxygen and consecutive cellular damage.

## 1. Introduction

Many proteins base their biological activity on creating metal complexes [1,2,3]. Metals in proteins can form strong bonds that maintain the tertiary structure of the proteins and can also bind to more than one polypeptide chain, creating the quaternary structure of oligomers [4,5]. Besides their structural role, metals are also required for the function of metalloproteins [6]. For example, the copper ion plays an electron-transporting role based on its oxidation-reduction potential in mitochondrial cytochrome c oxidase [7], plastocyanins [8], superoxide dismutase [9] and other copper enzymes [10]. Peptides bind metal ions, in some cases very strongly, due to the presence of a large number of donor atoms. The binding of metals can be realized by the peptide backbone and also by donors present in amino acid side chains (Lys, Arg, Glu, Asp), the imidazole group of histidine and the aromatic rings of amino acids (Phe, Tyr, Trp) [11,12,13,14]. Interestingly, some very short peptides, like the W-H-W sequence, can form stable complexes with transition metals like copper [15], related to a vast multitude of biologically relevant processes. For instance, such a sequence is present in some amyloid-β (Aβ) peptides [16], the copper(II)-binding domains of some pheromone (a-factor) analogues [17] and other tripeptide sequences that contribute significantly to many biological processes [18].

Furthermore, peptides with tryptophan (W) are known to bind non-covalently to DNA or RNA [19], which brings another dimension and angle of interest to the related structures.

Both the W-H-W peptide’s properties (copper binding and DNA/RNA interaction) and the fact that Cu^2+^ can efficiently cleave DNA or RNA backbones under certain conditions [20,21,22] stirred our interest in new W-H-W analogues.

We have noticed the structural and metal-coordination similarities between the histidine heterocycle and triazole. Namely, 1,2,3-triazoles possesses two nitrogen atoms capable of coordinating metals and represents a good ligand moiety for transition metal complexes [23,24]. They are often used in peptides to mimic a *trans*-amide bond [25,26,27] and as an attractive bridging group to link two pharmacophores [28,29]. More importantly, the introduction of 1,2,3-triazoles by copper(I)-catalysed ‘click’ chemistry (CuAAC) is often used for the introduction of various molecular tools, like fluorophores or similar. Thus, replacement of the central histidine in WHW by a propargyl analogue (precursor to 1,2,3-triazole) would be a convenient route to orthogonal late-peptide modification, provided that the 1,2,3-triazole will have similar Cu-binding properties as histidine. 

**Scheme 1:**
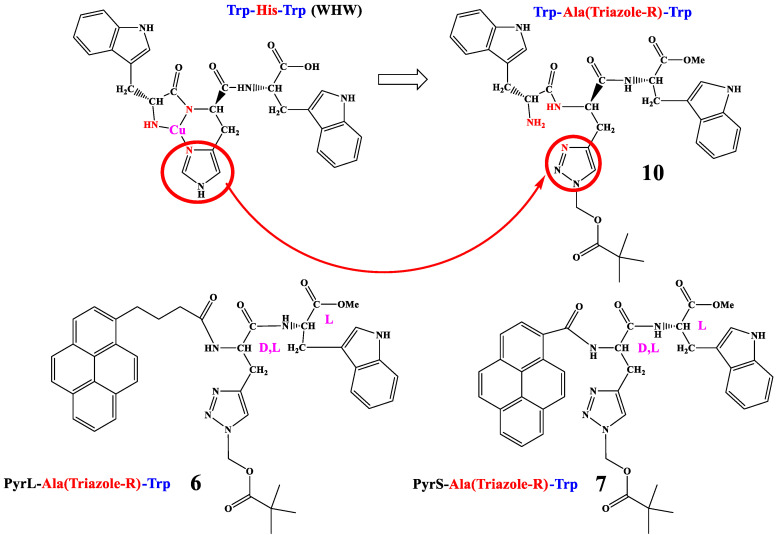
Comparison of the WHW peptide with the here-designed and prepared W-A(triazole)-W analogue **10**, its close pyrene analogue **6** (pyrene linker length analogous to the linker in **10** for Trp) and the short and rigid pyrene analogue **7**.

Therefore, in our design of novel WHW-based peptidoids (Scheme 1, **10**), the central histidine in WHW is replaced with the Ala-(triazole) motif, whereby triazole should mimic histidine in the coordination of copper cations, as demonstrated very recently for the FRH peptide [30]. The same work [30] demonstrated that such common ‘click’ chemistry (CuAAC) can be a very convenient synthetic route for the introduction of fluorophores into a peptide of interest while retaining its metal-binding properties.

Further, two analogous peptides were prepared, in which one tryptophan (W) fluorophore was replaced with pyrenes, giving the W-A(triazole)-Pyr motif (Scheme 1, **6** and **7**). An analogue with pyrene attached by a flexible and long linker (Scheme 1, **6**) corresponds nicely by the number of linker atoms to the W- A(triazole)-W peptide **10**; at variance to the short, rigid linker (**7**), the pyrene in that case being significantly closer to Cu(II) coordination site. The choice of the fluorophore was based on pyrene emission sensitivity to the microenvironment [31], pyrene being intensively used for various probing of DNA/RNA/proteins [32,33], as well as in theranostic agents [34]. Additionally, pyrene-peptide conjugates were recently efficiently applied for the fine sensing of various nucleic acid sequences [35,36,37]. Additionally, pyrenes may serve as anticancer agents and chemotherapeutics [38,39]. A particularly intriguing property of pyrene for this research is the ability to produce singlet oxygen upon irradiation [31], which could eventually lead to photo-induced DNA cleavage or bioactivity [39,40]. Taking into account recent advancements in Two-Photon-Absorption (TPA) techniques applied for photodynamic therapy (PDT) [41,42], pyrenes can now be applied as very efficient photosensitisers [40,41,42,43]. 

Taking into account that the Cu(II)-complexes of the here-prepared compounds could eventually also cleave ds-DNA or proteins of interest, and cause a Cu-controlled biological effect, the here-presented series of peptidoids has a dual mode of bio-activation, by Cu-cation and/or light irradiation.

## 2. Results

### 2.1. Synthesis

**Scheme 2:**
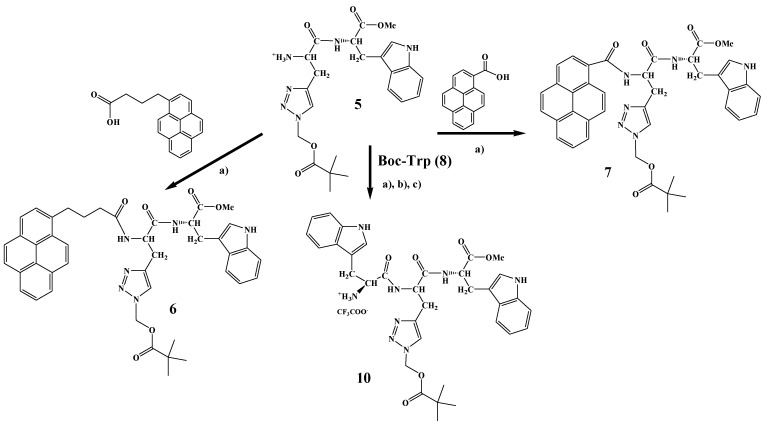
Synthesis of W-A(triazole)-Pyr conjugates **6** and **7** and W-A(triazole)-W tripeptide **10** starting from W-A(triazole) dipeptide **5** (Supplementary Information) (**a**) HOBt, HBTU, Et_3_N/CH_3_CN, rt, 16 h: **6** (66%), **7** (64%). **9** (63%); (**b**) Boc_2_O, 2M NaOH, dioxane/H_2_O; rt, 2 h; (**c**) TFA/CH_2_Cl_2_ (1:1), 20 h, 95%. The synthesis of 5 and 8 see in Supplementary Information.

Synthesis of the W-A(triazole) dipeptide **5** was executed in five steps from commercially available starting materials, *D,L*-propargylglycine (PG) and *L*-tryptophan (Supplementary Information Scheme S1). From the W-A(triazole) dipeptide **5,** synthesis branched in three directions (Scheme 2): a pyrene was attached to the free amino group of **5** by a longer and more flexible 1-pyrene butyric acid or a very short and rigid 1-pyrene carboxylic acid by a coupling reaction in the presence of HBTU/HOBt coupling reagents and triethylamine in acetonitrile, affording W-A(triazole)-Pyrenes **6** (66%) and **7** (64%), respectively. The same coupling protocol was used for the synthesis of the W-A(triazole)-W tripeptide **10**. For this purpose, dipeptide **5** was coupled with the Boc-protected [44] tryptophan **Boc-Trp (8)**, yielding conjugate **9** at 63% yield, which, upon deprotection with TFA/CH_2_Cl_2_, gave the desired W-A(triazole)-W tripeptide **10** (95%).

### 2.2. Spectrophotometric Characterisation

The studied peptides **6**, **7** and **10** were moderately soluble in water; thus, for easier handling, the stock solutions were prepared in DMSO (5 mM), and stored at +8 °C, and aliquots were diluted in an aqueous solution before the experiment. 

The absorbancies of buffered solutions of the studied **6**, **7** and **10** were proportional to their concentrations up to *c* = 1 × 10^−5^ M (Supplementary Information Figures S11 and S12). The absorption maxima and their corresponding molar extinction coefficients are given in Table 1.

**Figure 1:**
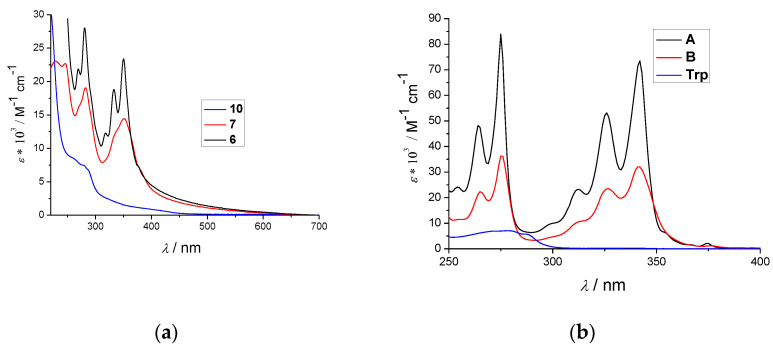
(**a**) UV/Vis spectra of studied peptides **6**, **7** and **10** in water (0.1% DMSO); (**b**) UV/Vis spectra of referent compounds 1-pyrenebutyric acid (**A**) and pyrenecarboxylic acid (**B**) and tryptophan (**Trp**) in water.

Comparison of the UV/vis spectra (Figure 1, Table 1) revealed pronounced differences between the referent chromophores (pyrene-carboxylic acids **A, B** and the amino acid **Trp**) and the corresponding peptides **6**, **7** and **10**, suggesting intramolecular aromatic stacking interactions in the peptides. For instance, the literature value for **Trp** in water at 280 nm is 5600 M^−1^cm^−1^; but here that obtained for bis-Trp **10** is 8322 M^−1^cm^−1^; thus 8322/5600 = 1.5, implying a strong hypochromic effect on the two Trp in the molecule, likely resulting from the intramolecular stacking interaction between the two Trp units.

Analogously, the UV/vis spectrum of rigid pyrene-peptide **7**, compared to referent **B** (Figure 1), showed a strong hypochromic effect and also strong broadening of absorption bands in the 300–360 nm range, accompanied by a complete loss of the fine vibronic structure of **B**. The UV/vis spectrum of peptide **6** (long and flexible pyrene linker) also showed hypochromicity in comparison to referent **A**; but unlike the rigid analogue **7**, the UV/vis spectrum of **6** mostly preserved the pyrene vibronic structure in the 300–360 nm range. Further, the temperature variation induced no effect on the UV/vis spectra of the referents **A** and **B**, at variance to the pronounced heating–cooling effects observed in the UV/vis spectra of the peptides **6**, **7** and **10**, the latter again confirming intramolecular aromatic stacking interactions of the chromophores (Supplementary Information Figure S13).

**Figure 2:**
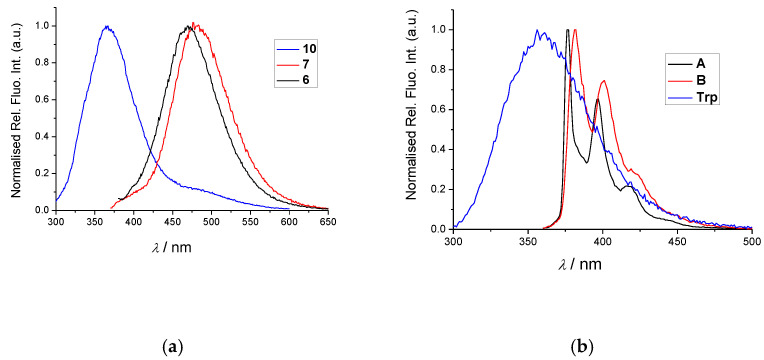
(**a**) Normalised fluorescence spectra of the studied peptides **6** (λ_exc_ = 352 nm), **7** (λ_exc_ = 350 nm) and **10** (λ_exc_ = 295 nm) in water (0.01% DMSO); (**b**) Normalised fluorescence spectra of the referent compounds 1-pyrenebutyric acid (**A**, λ_exc_ = 342 nm), pyrenecarboxylic acid (**B**, λ_exc_ = 341 nm) and tryptophan (**Trp**, λ_exc_ = 295 nm), in water. Normalisation was performed by dividing the complete spectrum by the maximal intensity of emission.

All the peptidoids possessed intrinsic fluorescence spectra in an aqueous solution (Figure 2, Supplementary Information Figures S14 and S15), with emission intensities proportional to their concentration up to *c* = 2 × 10^−6^ M; the excitation spectra closely resembled the corresponding UV/vis spectra (Figure 1, Supplementary Information Figures S14 and S15), supporting the assertion that the same chromophore is responsible to the emission and absorption of light. The fluorescence spectra of the peptides **6**, **7** and **10** (Figure 2a) strongly differed from the corresponding spectra of their referent compounds (Figure 2b), which could be attributed to the aforementioned intramolecular aromatic stacking interactions in **6**, **7** and **10**. Detailed analysis (Table 1, Figure 2) showed that bis-Trp **10**′s emission maximum is hypsochromically shifted by Δλ = −6 nm compared to the referent **Trp**, and the only emission spectrum of **10** showed a shoulder at λ > 450 nm, which could be attributed to the stacked Trp excimer. Additionally, the quantum yield of bis-Trp **10** (Table 1) was an order of magnitude lower than the referent Trp, which corroborated with the strong hypochromic effect of bis-Trp **10′s** UV spectrum (Table 1), supporting strong intramolecular aromatic stacking interactions between the two Trp units.

To characterise in more detail the emissive properties of the studied compounds, the decays of fluorescence were measured by time-correlated single-photon counting (TC-SPC) in both previously degassed and non-degassed aqueous solutions. Absolute quantum yields of fluorescence (Φ*_f_*) were measured at room temperature (25 °C) in sodium cacodylate buffer, pH = 7.0, I = 0.05 M, by the Integrating sphere SC-30 of Edinburgh FS5 spectrometer in a quartz cuvette of 10 mm path length. To prevent the incident light scattering at the liquid-air interface, sample 2 mL solutions were used (Table 1). 

The differences in emission spectra of the pyrene peptides **6** and **7** and their referent pyrene-analogues **A** and **B** were dramatic (Table 1, Figure 2): relative quantum yields of **6** or **7** were more than order of magnitude lower in respect to the referent pyrenes **A** and **B**; and the emission maxima of **6** and **7** were bathochromically shifted by Δλ ~ + 100 nm with respect to the emission of the referents **A** and **B**. Moreover, the fine vibronic splitting of the emission of the referent pyrenes **A** and **B** was completely lost in the peptides **6** and **7**. All these differences imply that the pyrenes of **6** and **7** are involved in aromatic stacking interactions [31]. In particular, strong emission of **6** and **7** at about 480–500 nm could be attributed to either (a) pyrene excimer emission resulting from intermolecular stacking of the two pyrenes [31,32,33,34,35] or (b) pyrene exciplex emission [35,45], in which a heterodimeric exciplex of pyrene and Trp is intramolecularly formed. Since the emission of **6** and **7** was proportional to their concentration (Supplementary Information Figures S14 and S15), that supports option (b); an intramolecularly formed exciplex formation between pyrene and Trp, eventually also including a triazole ring. A minor but measurable bathochromic shift of the rigid-analogue **7** with respect to the flexible-analogue **6**, corroborated with differences in their UV spectra (Figure 1), supports more intensive intramolecular interactions in **7**.

Comparison of the fluorescence decay results (*τ* in Table 1) obtained for bis-Trp **10** at both degassed and non-degassed conditions (Supplementary Information Figure S20) demonstrated a very similar two-exponential emission decay profile, pointing out that the presence of oxygen does not significantly influence the fluorescence decay properties of the fluorophores in **10**. The referent Trp is also characterised by a two-exponential emission decay profile, the second, longer-lived transition (τ = 3 ns) being somewhat shorter in comparison to bis-Trp **10** (τ = 4 ns).

The pyrene analogues’ fluorescence decay results were dominated by pyrene emission over Trp, due to the much higher absorptivity of pyrene, while overlapping in UV maxima. The pyrene analogues **A, B**, **6** and **7**′s fluorescence decay results were not sensitive to the presence of oxygen (Supplementary Information Figures S16–S19), all showing two-exponential decay; the only exceptions being the mono-exponential decay profiles of **7** in the absence of oxygen and **A** in the presence of oxygen—both likely due to the low percentage of second emissive species. The fluorescence decay profiles of **6** and **7** differ significantly in comparison to the referents **A** and **B** (Table 1), in line with the proposed intramolecular exciplexes of pyrene in **6** and **7**, which strongly altered the electronic properties of the pyrene.

### 2.3. Binding of Cu^2+^ Cation

As described in the Introduction and shown in Scheme 1, the studied peptides should bind to Cu^2+^, and therefore we took advantage of the peptides’ intrinsic fluorescence and performed fluorimetric titrations. For comparison reasons we also performed titrations of the referent compounds 1-pyrenebutyric acid (**A**, λ_exc_ = 342 nm) and pyrenecarboxylic acid (**B**, λ_exc_ = 341 nm) with a Cu^2+^ cation, which, as expected, did not yield any fluorescence change in the pyrene (data not shown). 

**Figure 3:**
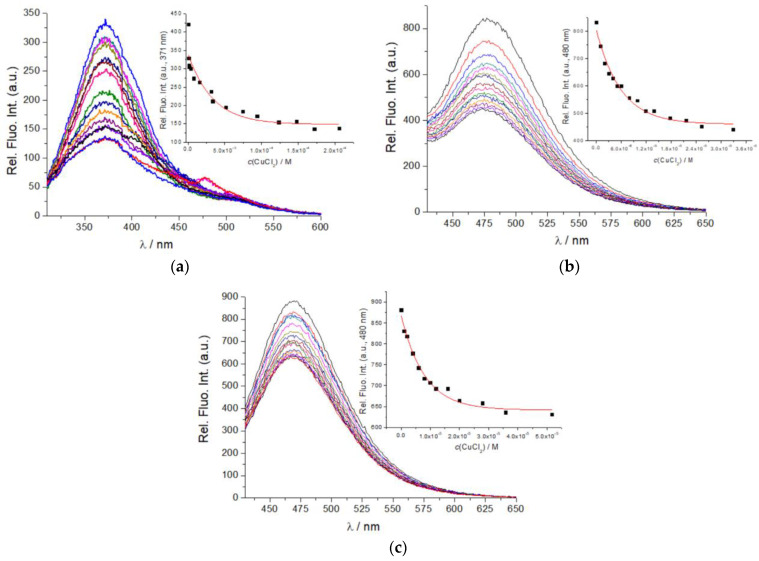
(**a**) Fluorimetric titration of (**a**) **10** (*c* = 5× 10^−6^ M; λ_exc_ = 295 nm) with CuCl_2_. dependence of fluorescence at λ_max_ = 371 nm on *c*(CuCl_2_); (**b**) Fluorimetric titration of **7** (*c* = 1 × 10^−6^ M; λ_exc_ = 352 nm) with CuCl_2_, dependence of fluorescence at λ_max_ = 480 nm on *c*(CuCl_2_); (**c**) Fluorimetric titration of **6** (*c* = 1 × 10^−6^ M; λ_exc_ = 350 nm) with CuCl_2_, dependence of fluorescence at λ_max_ = 470 nm on *c*(CuCl_2_). Performed at pH = 7.0, sodium cacodylate buffer, *I* = 0.05 M.

The emission of all studied peptides was strongly quenched by the addition of the Cu^2+^ cation (Figure 3); however, the emission spectra retained their general shape and emission maxima. A referent experiment with WHW (Supplementary Information Figure S21) showed that the emission of tryptophan was completely quenched by the Cu cation, at variance to the analogue **10**.

All titration data excellently fitted to 1:1 stoichiometry, giving strong binding constants (Figure 3, right); the affinity of the bis-tryptophan analogue (**10**) was about an order of magnitude lower (log*K* = 5.2 M^−1^) in comparison to the affinities of the pyrene analogues **6** and **7** (log*K* = 6.9 and 6.8 M^−1^, respectively). With the addition of Cu^2+^, the fluorescence lifetimes of the pyrene fluorophores of **6** and **7** did not change significantly (Supplementary Information Table S1), suggesting a static quenching mechanism [31].

### 2.4. Study of Interactions of ***6***, ***7*** and ***10*** with Ds-DNA and Ds-RNA in the Presence/Absence of Cu^2+^

#### 2.4.1. Fluorimetric Titrations

The addition of any ds-DNA/RNA to any of the studied compounds generally resulted in quenching their emission. Titration data were fitted by non-linear regression to the Scatchard equation [46], yielding the binding constants (Table 2).

The bis-Trp derivative **10**′s emission was quenched only at high concentrations of ct-DNA; very similar to the emission of the parent WHW (Supplementary Information Figure S22), both yielding rather low binding constants (Table 2). 

**Figure 4:**
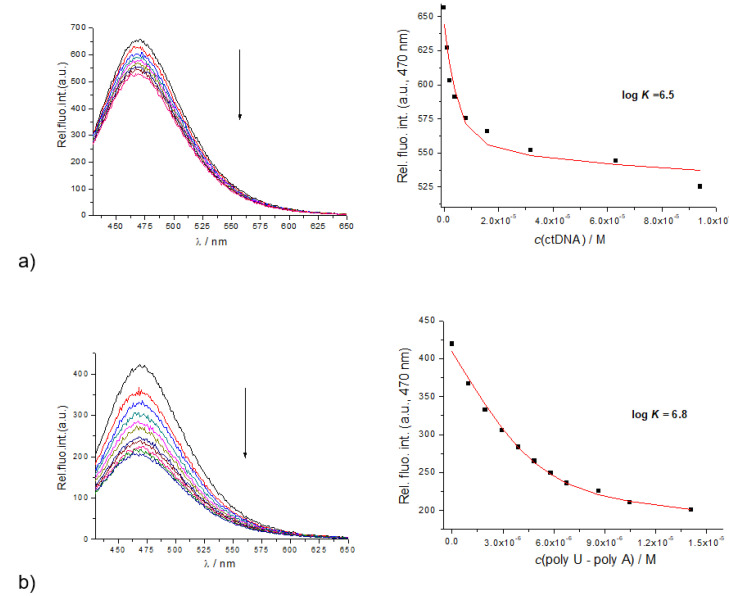
(**a**) Fluorimetric titrations of **6** (*c* = 1 × 10^−6^ M; λ_exc_ = 350 nm) with a) ctDNA; (**b**) with poly A –poly. U. RIGHT: Dependence of emission intensity at λ_max_ = 470 nm on c(polynucleotide), (–) data fit to the Scatchard equation [46]. Performed at pH = 7.0, buffer sodium cacodylate, *I* = 0.05 M, 0.04% DMSO.

The emission of the pyrene analogues **6** and **7** changed at two orders of magnitude lower concentrations of ds-DNA or ds-RNA, pointing to a much higher affinity than **10** (Table 2). Intriguingly, the emissions of **6** and **7** were much more strongly quenched by AU-RNA in comparison to ct-DNA (Figure 4 and Supplementary Information Figure S23).

We also prepared complexes of **6** and **7** with a 4-fold excess of Cu^2+^ (according to the binding constants determined in Section 2.3) and subsequently performed fluorimetric titrations with ds-DNA/RNA, which yielded only minor changes of **6** and **7**′s pyrene emission (Supplementary Information Figures S24–S27). Such small emission changes were attributed to the effect of the previously added Cu^2+^, which had already quenched pyrene emission to a large extent. Again, the addition of AU-RNA resulted in more noticeable quenching; however, small emission changes allowed only estimation of the binding constants within log *K_s_* = 5–6.

With the addition of ds-DNA (with Cu^2+^ or without), the fluorescence lifetimes of the pyrene fluorophores in **6** and **7** did not change significantly (Supplementary Information Table S1), suggesting a static quenching mechanism [31].

#### 2.4.2. Circular Dichroism (CD) Experiments

To investigate the mode of binding of the peptides **6**, **7** and **10** to ct-DNA and poly A–poly U in more structural detail, we used circular dichroism (CD) spectroscopy. CD spectroscopy is a useful analytical tool in the study of the binding of small molecules to chiral macromolecules such as DNA [47], since it can provide information on the mode of binding to a polynucleotide, with distinctive spectral differences for intercalators and groove-binding derivatives [48,49]. The peptides **6**, **7** and **10** are chiral; however, they showed no measurable CD bands within the 240–400 nm range (Supplementary Information Figures S28–S30), thus not interfering with the CD bands of DNA/RNA in the 240–290 nm range, nor with the eventual induced CD bands of the pyrenes, which could appear >300 nm upon binding to ds-DNA/RNA.

The addition of **6**, **7** or **10** to any ds-DNA or ds-RNA did not significantly change the CD spectrum of the polynucleotide (Supplementary Information Figures S27–S29), which excluded eventual intercalation of pyrene or tryptophan units between the DNA/RNA basepairs [48]. Additionally, upon the addition of **6** or **7** to ds-DNA/RNA, no ICD bands > 300 nm were observed, thus suggesting non-uniform orientation of the pyrene units with respect to the polynucleotide chiral axis [48,49]. Similarly, the addition of the pre-prepared **6**/Cu^2+^, **7**/Cu^2+^ or **10**/Cu^2+^ complexes caused only minor decreases of the ds-DNA/RNA CD spectra, with no ICD bands > 300 nm, thus generally suggesting that binding of either free **6**, **7** or **10** or their Cu^2+^ complexes causes only minor disturbance of the ds-DNA or ds-DNA helical structure. Such an effect could be correlated to the insertion of the studied peptidoids within the ds-DNA minor groove or ds-RNA major groove [48,49].

To check the binding mode of the studied peptidoids, we performed competition experiments with DAPI, a specific DNA minor-groove-binder [50], and, for monitoring displacement, we opted for circular dichroism as a fluorescence-independent method. Indeed, the addition of **6** or **7** displaced DAPI, as monitored by the disappearance of the DAPI ICD band (Supplementary Information Figure S34). We analysed the results by means of the Indicator Displacement Assay (IDA) methodology [51]. Observed IDA50% values (IDA50% = 0.02–0.01) suggested an order-of-magnitude stronger binding of DAPI with respect to **6** and **7**, which is in accordance with the determined binding constants. Thus, the competition experiments additionally supported the binding of the novel peptidoids within the DNA minor groove and also corroborated with the binding constants determined by fluorimetric titrations. Addition of **10** very weakly displaced DAPI (Supplementary Information Figure S34), as expected from the much weaker binding (Table 2). Attempted competition experiments involving the addition of peptidoid/copper complexes to DNA/DAPI were hampered due to only Cu^2+^ displacing DAPI, attributed to competitive interactions with DNA phosphates.

#### 2.4.3. Thermal Denaturation of Ds-DNA/RNA

Thermal denaturation experiments provided information about the ds-polynucleotide helix’s thermal stability as a function of interaction with the added small molecules [52]. The difference between the *T*_m_ value of free ds-polynucleotide and a complex with a small molecule (Δ*T*_m_ value) is an important factor in the characterization of small molecule/ds-polynucleotide interactions. For instance, moderate to strong stabilization (Δ*T*_m_ > 5 °C) supports pronounced intercalative or minor-groove-binding interaction, [53] whereas weak or negligible stabilization (Δ*T*_m_ = 0–5 °C) suggests a binding process driven mostly by a hydrophobic effect accompanied by weak H-bonding and/or electrostatic interactions—usually excluding classical intercalation as a binding mode. 

Thermal denaturation experiments (Table 3, Supplementary Information Figures S31–S33) revealed that the addition of **6**, **7** or **10** did not stabilise ct-DNA against thermal denaturation.

However, if **6**, **7** or **10** were pre-incubated before the thermal denaturation experiments with a five-fold excess of Cu^2+^, ensuring the formation of a peptidoid/Cu^2+^ complex (according to the determined binding constants: Table 2), the so-prepared complexes did influence the thermal denaturation properties of ds-DNA or ds-RNA (Table 3, Supplementary Information Figures S32 and S33), whereas it should be stressed that addition of only Cu^2+^ to any ds-DNA/RNA did not have any impact on the polynucleotide denaturation profile. The addition of the rigid **7**/Cu^2+^ complex yielded only minor stabilisation of ds-DNA/RNA, whereas upon addition of the more flexible **6**/Cu^2+^ complex, the thermal denaturation profiles of all polynucleotides strongly changed (Table 3, Supplementary Information Figure S32). 

**Figure 5:**
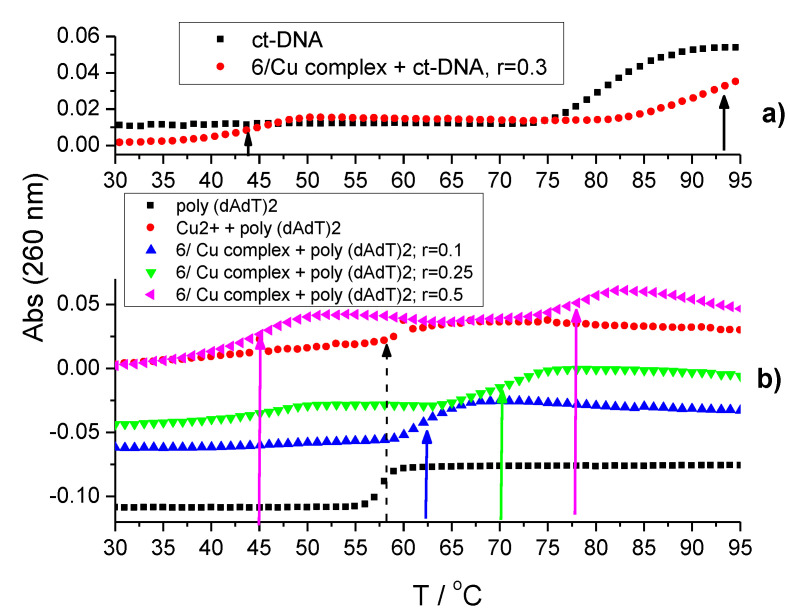

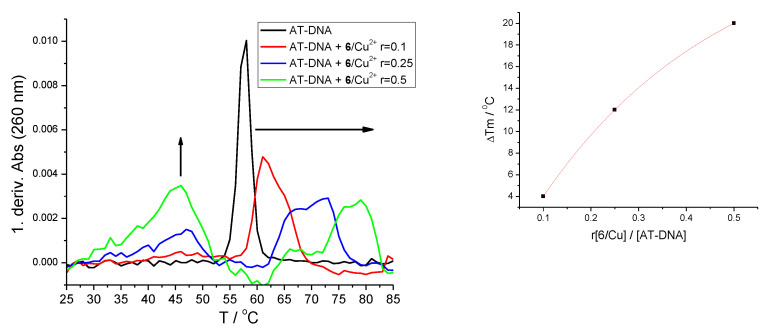
(**UP**) Thermal denaturation curves of (**a**) ct−DNA (*c*(ct-DNA) = 2.5 × 10^−5^M, r _[**6/Cu)**]/[ct-DNA]_ = 0.25) and (**b**) poly dAdT−poly dAdT (*c*(AT−DNA) = 2.5 × 10^−5^ M, r _[**6/Cu)**]/[AT-DNA]_ = 0.1; 0.25; 0.5. Performed at at pH = 7.0, sodium cacodylate buffer, *I* = 0.05 M. (**DOWN**): (**left**) The first-derivative analysis of the AT−DNA experiment results; (**right**) dependence of the stabilisation effect (∆*Tm*) on the ratio r of [**6**/Cu]/[AT−DNA].

Since the ct-DNA was stabilised beyond the measuring ability of the method (*Tm* > 100 °C, Table 3), an additional experiment with AT-DNA and the **6**/Cu^2+^ complex was performed (Figure 5), the lower denaturation point of AT-DNA allowing for a more detailed analysis of the results. 

Intriguingly, the addition of the **6**/Cu complex induced, in both ct-DNA and AT-DNA, biphasic denaturation curves (Figure 5, Table 3), which showed both strong destabilisation (up to −38 °C) and strong stabilisation (up to +20 °C). A detailed analysis showed that the stabilisation effect was strongly nonlinearly dependent on the ratio r of _[**6/Cu)**]/[AT-DNA]_ (Figure 5 down, right), at variance with the destabilisation effect, which was independent of ratio r (Figure 5 down, left). The addition of the **6**/Cu^2+^ complex caused only stabilisation of ds-RNA; thus the stabilisation/destabilisation effect of the **6**/Cu^2+^ complex is specific for ds-DNAs. The binding sites of ds-DNA or ds-RNA for small molecules differ due to the considerable difference in the polynucleotide structure (Supplementary Information Table S2): the AT-DNA minor groove or AU-RNA major groove are available for small molecule insertion [54], whereby the much deeper major groove of RNA allows deep insertion of small molecules and thus better exclusion from the bulk water, in that way distancing it from the water-exposed phosphate backbone. Consequently, the **6**/Cu^2+^ complex deeply inserted into the ds-RNA major groove is not close to the negatively charged RNA-backbone, diminishing the efficient electrostatic interaction between the copper cation and phosphate anions. 

At variance to the binding to ds-RNA, the **6**/Cu^2+^ complex was strongly bound inside a much shallower ds-DNA minor groove (Supplementary Information Table S2), the copper cation significantly more strongly contributing to the stabilization of the double-stranded helix by electrostatic interactions with the negatively charged DNA backbone. However, during the heating of the sample, copper-mediated oxidative cleavage of the polynucleotide backbone partially cleaved ds-DNAs into shorter fragments with lower melting points, which was manifested as a “destabilisation” transition. This copper-mediated oxidative cleavage of the polynucleotide backbone was not observed for ds-RNA, very likely due to the much deeper insertion of the **6**/Cu^2+^ complex into the RNA major groove.

#### 2.4.4. UV Exposure of Compounds **6** and **7** and/or Complexes with Cu Cation Induces Plasmid DNA Breaks

Photoactivated pyrene moieties can cause DNA breaks as a consequence of singlet oxygen production [39,40]. On the other hand, complexes of peptides with Cu cations can cause oxidative cleavage of the DNA backbone, also resulting in plasmid DNA disruptions [20,21]. We showed that the studied pyrene derivatives **6** and **7** efficiently bound to ds-DNA, even if present as **6**/Cu^2+^ or **7**/Cu^2+^ complexes (Table 2 and Table 3), thus bringing both pyrene and Cu^2+^ into the proximity of the DNA backbone.

Therefore, we assessed the plasmid DNA cleavage by (**i**) UV-light irradiation (30 s) of plasmid DNA treated with **6** or **7** or (**ii**) plasmid DNA mixing with pre-prepared **6**/Cu^2+^ or **7**/Cu^2+^ at 37 or 50 °C. 

**Figure 6:**
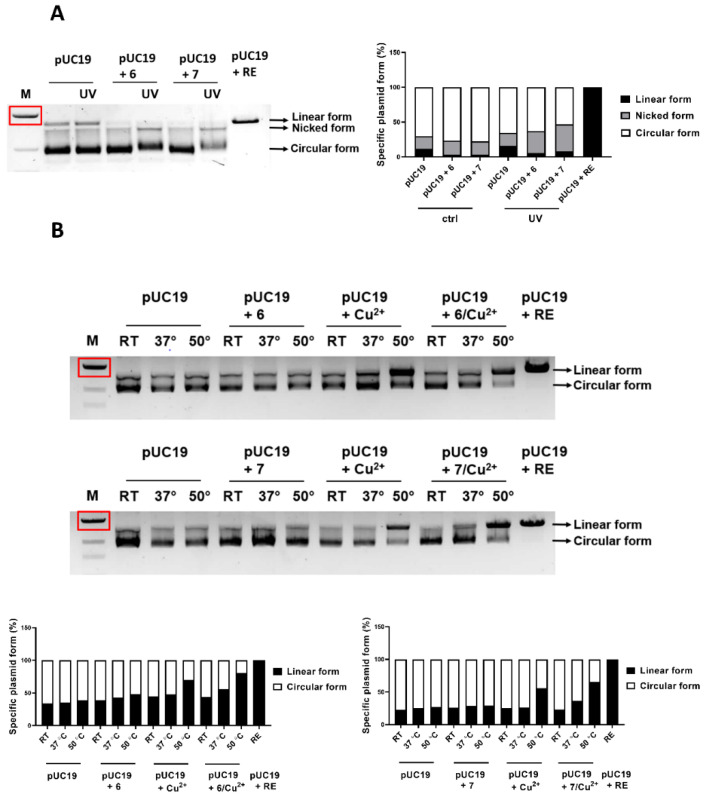
Representative agarose gel and quantitative analysis of supercoiled pUC19 (2686 bp) plasmid DNA incubated with 6 and 7, with or without (**A**) exposure to UV light (30 sec) or (**B**) presence of the Cu^2+^ cation at various temperatures (room temperature, 37 or 50 °C) for 24h. For (**A**) assay, 30 μM of the tested compound was incubated with 1 μg of pUC19, while for (**B**) assay, 10 μM of **6**, **7** and the complexes **6**/Cu^2+^ or **7**/Cu^2+^ were incubated with 1 μg of pUC19. A plasmid without compounds, a plasmid with only a Cu^2+^ cation and a plasmid linearized with restriction enzyme (RE) served as controls. M—GelPilot DNA Molecular Weight Marker 1kb (Qiagen, Germantown, MD, USA). The red rectangle represents a DNA fragment of 3000 bp. Quantitative analysis of specific plasmid forms was performed in ImageJ software 1.53e. Each form is presented as the percentage of total pUC19 forms per condition (white, grey and black columns). Graphs were constructed in GraphPad Prism 8. Representative data of three independent experiments which yielded similar results are shown.

The progress of the plasmid DNA cleavage was monitored by gel electrophoresis, indicating that **6** and **7** have photoinduced nuclease activity (Figure 6A). Three forms of plasmid DNA were visible on the agarose gel: linear form, nicked and supercoiled circular form. All three forms were quantitatively analysed and are presented in the graph. Because UV exposure for 30 s did not influence the plasmid DNA itself, it is evident that the creation and increasing amount of the nicked plasmid DNA form was induced solely by the photoactivated **6** and **7**, most likely due to the pyrene-mediated singlet oxygen production. However, **6** and **7** were less active than some previously reported pyrene analogues [31,39,40], likely due to the binding of **6** and **7** inside the DNA minor groove in self-folded form, where the pyrene is partially intramolecularly stacked with tryptophan. Such sterically restricted conformation inhibits the full effect of pyrene-mediated oxygen production and consequently causes only minor nicking of plasmid DNA, instead of full cleavage and DNA-linearization of, e.g., pyrene-intercalators.

Further, we assessed if pre-prepared **6**/Cu^2+^ or **7**/Cu^2+^ could induce thermal-dependent plasmid DNA linearization (Figure 6B). Samples of plasmid DNA with added **6**/Cu^2+^ or **7**/Cu^2+^ were incubated at room temperature (RT), 37 °C or 50 °C for 24h. Plasmid DNA pUC19 (2686 bp) without compounds, plasmid DNA with only a Cu^2+^ cation and plasmid DNA linearized with restriction enzyme (RE) served as negative or positive controls. Results indicated that **6**/Cu^2+^ or **7**/Cu^2+^ do not cleave plasmid DNA detectably, beyond the effect of the Cu^2+^ cation alone, which was evident only at 50 °C (>50% of the linear form). Again, it seems that intramolecularly self-folded **6**/Cu^2+^ or **7**/Cu^2+^ inside the DNA minor groove prevents efficient oxidative cleavage of the DNA backbone.

### 2.5. Biological Experiments

To study the cytotoxic effect of compounds **6** and **7**, we first confirmed that these compounds successfully enter A549 cells within 90 min of incubation at 37 °C (Supplementary Information Figure S35). It is essential to emphasize that to achieve this goal, we took advantage of the fluorescence emission properties of **6** and **7**, which are due to the pyrene-exciplex formation being strongly bathochromically shifted (Figure 2 left; λ_em_ = 450–550 nm) with respect to the referent pyrenes **A** and **B** (Figure 2 right; λ_em_ = 380–420 nm). This emission property of **6** and **7**, as well as bathochromically shifted UV/Vis spectra (Figure 1), allowed excitation of **6** and **7** at λ_exc_ = 405 nm and, in this way, omitted the cellular autofluorescence background by fine tuning of the microscope parameters on non-treated cells. Strong cell autofluorescence at the excitation/emission (295 nm/350 nm) wavelengths used for compound **10** hampered the confocal microscope experiment.

**Figure 7:**
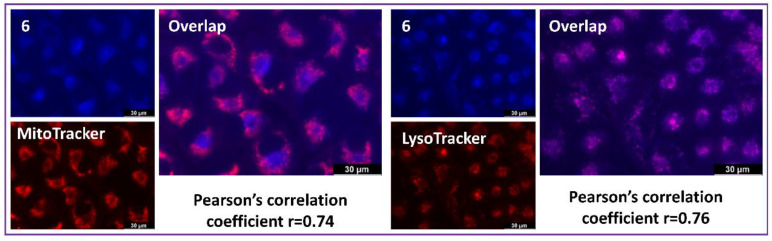
Intracellular localization of **6** (shown in blue) in A549 cells. Cells were treated with 10 µM of the compound for 90 min at 37 °C and co-localization with mitochondria (MitoTracker) or lysosomes (LysoTracker), all shown in red, was monitored by confocal microscopy. (λ_exc_ = 348 nm, λ_em_ = 430–550 nm). Analysis was performed using ImageJ software and the appropriate JACoP plugin. Co-localization was assessed by the Pearson correlation coefficient.

To determine the specific intracellular localization of the compounds 6 and 7, we live imaged A549 cells treated with **6** and **7** and performed co-localization experiments with stains specific for different cytoplasmic organelles, namely mitochondria (MitoTracker Deep Red) and lysosomes (LysoTracker Deep Red). Since strong emission was necessary for comparison with Tracker stains, for this experiment we used 348 nm excitation, tuning the instrument on the non-treated cells to avoid autofluorescence. As shown in Figure 7, compound **6** was distributed equally between mitochondria and lysosomes, exhibiting no obvious selectivity. Compound **7** showed strong co-localization with lysosomes (Supplementary Information Figure S36). Co-localization of **7** with MitoTracker Deep Red showed inconclusive results.

**Figure 8:**
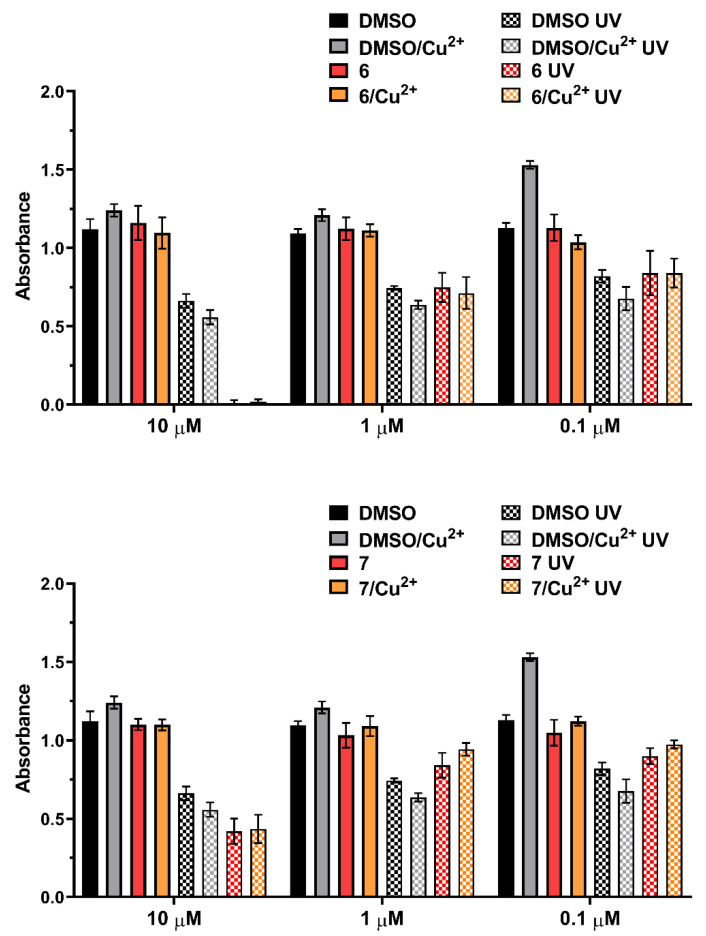
Cell survival of A549 cells treated with different concentrations of **6, 6/Cu** complex, **7** and **7/Cu** complex with or without UV light irradiation (350 nm) for 30 min. Measured absorbance (λ = 600 nm) directly correlates with cell survival. Data are presented as mean ± SD made in three replicates. Control samples are cells treated with DMSO in the same dilution as a tested compound. Representative data of two independent experiments which yielded similar results are shown.

Next, we treated A549 cells with 10 μM, 1 μM and 0.1 μM of the corresponding compound and assessed cell survival by MTT assay (Figure 8). Compounds **6** and **7** did not show significant cell toxicity in human A549 in either of the tested concentrations. We also assessed the influence of the Cu^2+^ cation and UV irradiation on the antiproliferative effects of the tested compounds against A549 cells. Without exposure to UV light, all tested compounds did not show a cytotoxic effect on A549, regardless of the concentration or presence of the Cu^2+^ cation. However, 30 min/day UV irradiation of cells treated with the compounds resulted in a very strong cytotoxic effect of **6** and a moderate effect of **7** at 10 μM concentration. We observed that the addition of the Cu^2+^ cation did not influence the cytotoxicity of **6** or **7** when incubated with A549 at 37 °C, regardless of the UV exposure.

## 3. Materials and Methods

### 3.1. General

Solvents were distilled from appropriate drying agents shortly before use. TLC was carried out on DC-plastikfolien Kieselgel 60 F254 and preparative thick-layer (2 mm) chromatography was performed on Merck 60 F254 plates (Merck KGaA, Darmstadt, Germany). (Merck, Merck KGaA, Darmstadt, Germany). NMR spectra were recorded on AV600 and AV300 MHz spectrometers (Bruker BioSpin GmbH, Rheinstetten, Germany), operating at 150.92 or 75.47 MHz for ^13^C and 600.13 or 300.13 MHz for ^1^H nuclei using DMSO-*d*_6_ as the internal standard (labels in the spectra: Trp = tryptophan ring; Pyr = pyrene ring). Mass spectrometry was performed on the Agilent 6410 Triple Quad mass spectrometer (Agilent Technologies, Santa Clara, CA, USA) and high-resolution mass spectra (HRMS) were obtained using a Q-Tof2 hybrid quadrupole time-of-flight mass spectrometer (Micromass, Cary, NC, USA). WHW was purchased from GenScript, Treubstraat 1, 1st floor. 2288EG, Rijswijk, the Netherlands, as a 95% pure white solid (TFA salt). Experimental procedures for preparing known compounds **1**, **2** and **8** are given in the Supplementary Information.

### 3.2. Synthesis

Methyl (2-((*tert*-butoxycarbonyl)amino)pent-4-ynoyl)-*L*-tryptophanate (3).

Boc-protected PG **1** (83 mg, 0.39 mmol) and tryptophan methyl ester hydrochloride **2** (100 mg, 0.39 mmol) were dissolved in dry CH_3_CN (7 mL) under argon and HOBt (97%, 54 mg, 0.39 mmol), HBTU (98%, 151 mg, 0.39 mmol) and dry Et_3_N (0.22 mL, 1.56 mmol) were added. The reaction mixture was stirred at room temperature for 16 h. Trp-A(alkyne) dipeptide **3** (114 mg, 71%) was isolated by preparative chromatography (CH_2_Cl_2_/CH_3_OH 9:1) as a yellow oil: *R*_f_ = 0.86 (CH_2_Cl_2_/CH_3_OH 9:1); ^1^H NMR (DMSO-*d*_6_) *δ*/ppm: 10.87 (s, 1H, NH-Trp), 8.32 and 8.25 (2xd, *J* = 7.7, 7.4 Hz, 1H, NH-C=O), 7.47 (d, *J* = 7.9 Hz, 1H, Trp), 7.33 (dd, *J* = 8.1, 2.6 Hz, 1H, Trp), 7.18–7.11 (m, 1H, Trp), 7.06 (t, *J* = 7.5 Hz, 1H, Trp), 7.01–6.96 (m, 1H, Trp), 6.92 and 6.86 (2xd, *J* = 8.6, 8.7 Hz, 1H, NH), 4.55–4.49 (m, 1H, CH), 4.20–4.01 (m, 1H, CH), 3.58 and 3.55 (2xs, 3H, OCH_3_), 3.20–3.01 (m, 2H, CH_2_), 2.81 and 2.75 (2xbrs, 1H, HC≡C-), 2.45–2.22 (m, 2H, CH_2_), 1.38 (s, 9H, Me_3_C-O-); ^13^C NMR (DMSO-*d*_6_) *δ*/ppm: 172.0 and 171.9 (MeO-C=O), 170.4 and 170.2 (NH-C=O), 155.1 and 155.0 (Me_3_C-O-C=O), 136.1 (Cq, Trp), 127.0 (Cq, Trp), 123.7 (CH, Trp), 120.9 (CH, Trp), 118.4 and 118.37 (CH, Trp), 117.9 (CH, Trp), 111.4 and 111.37 (CH, Trp), 109.1 and 109.0 (Cq, Trp), 80.8 and 80.7 (Cq, Me_3_C-O-), 78.3 and 78.27 (HC≡C-), 72.6 and 72.58 (HC≡C-), 53.1 and 53.0 (CH or OCH_3_), 52.9 and 52.7 (CH or OCH_3_), 51.8 and 51.78 (CH), 28.1 (Me_3_C-O-), 27.1 and 26.9 (CH_2_), 21.9 (CH_2_); (see Supporting Information Figure S3a,b). ESI-MS: *m*/*z* calcd. for C_22_H_27_N_3_NaO_5_ [M+Na]^+^ 436.18, found 436.4.

4-(3-(((*S*)-3-(1*H*-Indol-3-yl)-1-methoxy-1-oxopropan-2-yl)amino)-2-((*tert*-butoxycarbonyl)amino)-3-oxopropyl)-1*H*-1,2,3-triazol-1-yl)methyl pivalate (4).

To a stirred solution of chloromethyl pivalate (0.104 mL, 0.7 mmol, 97%) in EtOH/H_2_O (7:3, 1.8 mL), NaN_3_ (45 mg, 0.7 mmol), CuI (13 mg 0.07 mmol), sodium ascorbate (7 mg. 0.035 mmol, 99%) and *N*,*N*’-dimethylethylenediamine (DMEDA) (12 μL, 0.105 mmol, 95%) were added. After the addition of DMEDA, the solution turns blue, then green and finally yellow. After stirring for 30 min, the clear yellow solution changes colour to green. The mixture was heated at 100 °C for 1 h, then alkyne **3** (114 mg 0.28 mmol), sodium ascorbate (7 mg. 0.035 mmol, 99%), CuI (13 mg 0.07 mmol) and DMEDA (12 μL, 0.105 mmol, 95%) were added. The mixture was heated at 100 °C for 30 min. and, after cooling, the resulting solid was filtered off. The crude product was filtered through a short SiO_2_ column and evaporated. Recrystallization from methanol gave compound **4** (139 mg 87%) as a yellow solid: *R*_f_ = 0.72 (CH_2_Cl_2_/CH_3_OH 9:1); ^1^H NMR (DMSO-*d*_6_) *δ*/ppm: 10.95 (brs, 1H, NH-Trp), 8.49–8.23 (m, 1H, NH-C=O), 7.85 and 7.73 (2xs, 1H, H-5 triazole), 7.48 (d, *J* = 7.9 Hz, 1H, Trp), 7.33 (dd, *J* = 8.0, 0.9 Hz, 1H, Trp), 7.15 (dd, *J* = 13.8, 2.0 Hz, 1H, Trp), 7.11–7.01 (m, 1H, Trp), 7.00–6.85 (m, 2H, Trp and NH), 6.26 and 6.22 (2xs, 2H, CH_2_), 4.51 (dd, *J* = 12.9, 6.6 Hz, 1H, CH), 4.36–4.10 (m, 1H, CH), 3.58 and 3.55 (2xs, 3H, OCH_3_), 3.18–2.73 (m, 4H, 2x CH_2_), 1.32 (brs, 9H, Me_3_C-O-), 1.11 and 1.10 (2xs, 9H, Me_3_C=O); ^13^C NMR (DMSO-*d*_6_) *δ*/ppm: 176.4 and 176.39 (C=O, Piv), 172.1 and 172.05 (MeO-C=O), 171.3 and 171.0 (NH-C=O), 155.1 (Me_3_C-O-C=O), 143.7 and 143.67 (C4, triazole), 136.0 and 136.05 (Cq, Trp), 127.0 and 126.98 (Cq, Trp), 123.9 and 123.8 (C5, triazole), 123.75 and 123.72 (CH, Trp), 120.9 (CH, Trp), 118.4 (CH, Trp), 117.9 and 117.89 (CH, Trp), 111.4 (CH, Trp), 109.1 and 109.06 (Cq, Trp), 78.2 (Cq, Me_3_C-O-), 69.8 and 69.79 (N-CH_2_-O), 53.8 and 53.6 (CH), 53.1 and 53.0 (CH), 51.82 and 51.80 (OCH_3_), 38.2 and 38.1 (Me_3_C-C=O), 28.1 (Me_3_C-O-), 28.0 (CH_2_), 27.4 (CH_2_), 26.4 (Me_3_C-C=O); (see Supporting Information Figure S4). ESI-MS: *m*/*z* calcd. for C_28_H_39_N_6_O_7_ [M+H]^+^ 571.3, found 571.5.

-(((*S*)-3-(1*H*-Indol-3-yl)-1-methoxy-1-oxopropan-2-yl)amino)-1-oxo-3-(1-((pivaloyloxy)methyl)-1*H*-1,2,3-triazol-4-yl)propan-2-aminium 2,2,2-trifluoroacetate (5).

Compound **4** (139 mg, 0.24 mmol) was dissolved in a 1:1 mixture of TFA/CH_2_Cl_2_ (6 mL) and stirred at room temperature for 20 h. After removal of remaining TFA under reduced pressure, W-A(triazole) dipeptide **5** (140 mg, 100%) was obtained as a dark blue solid: *R*_f_ = 0.45 (CH_2_Cl_2_/CH_3_OH 9:1); ^1^H NMR (DMSO-*d*_6_) *δ*/ppm: 10.92 (s, 1H, NH-Trp), 9.11–8.87 (m, 1H, NH-C=O), 8.17 (brs, 3H, NH_3_^+^), 8.02 and 7.71 (2xs, 1H, H-5 triazole), 7.52–7.48 (m, 1H, Trp), 7.35 (d, *J* = 8.0 Hz, 1H, Trp), 7.16 (dd, *J* = 11.2, 2.2 Hz, 1H, Trp), 7.10–7.04 (m, 1H, Trp), 7.02–6.97 (m, 1H, Trp), 6.28 and 6.19 (2xs, 2H, CH_2_), 4.71–4.35 (m, 1H, CH), 4.13 (brs, 1H, CH), 3.61 and 3.60 (2xs, 3H, OCH_3_), 3.23–3.01 (m, 4H, 2x CH_2_), 1.13 and 1.11 (2xs, 9H, Me_3_C=O); ^13^C NMR (DMSO-*d*_6_) *δ*/ppm: 176.5 and 176.4 (C=O, Piv), 171.7 and 171.67 (Me-O-C=O), 167.9 and 167.6 (NH-C=O), 158.7, 158.4, 158.1 and 157.7 (CF_3_-C=O), 141.0 and 140.9 (C4, triazole), 136.2 and 136.1 (Cq, Trp), 126.9 (Cq, Trp), 125.0 and 124.7 (C5, triazole), 124.0 (CH, Trp), 121.1 (CH, Trp), 118.52 and 118.5 (CH, Trp), 118.0 and 117.9 (CH, Trp), 111.5 (CH, Trp), 108.84 and 108.81 (Cq, Trp), 69.9 (N-CH_2_-O), 53.3 and 53.1 (CH), 52.1 and 52.0 (CH), 51.7 and 51.6 (OCH_3_), 38.3 and 38.2 (Me_3_C-C=O), 27.5 and 27.3 (CH_2_), 27.2 and 27.0 (CH_2_), 26.5 (Me_3_C-C=O); (see Supporting Information Figure S5). ESI-MS: *m*/*z* calcd. for C_23_H_31_N_6_O_5_^+^ [M^+^] 471.24, found 471.4.

4-(3-(((*S*)-3-(1*H*-Indol-3-yl)-1-methoxy-1-oxopropan-2-yl)amino)-3-oxo-2-(4-(pyren-1-yl)butanamido)propyl)-1*H*-1,2,3-triazol-1-yl)methyl pivalate (6).

Compound **5** (236 mg, 0.4 mmol) and 1-pyrenebutyric acid **7** (119 mg, 0.4 mmol, 97%) were dissolved in dry CH_3_CN (10 mL) under argon and HOBt (56 mg, 0.4 mmol, 97%), HBTU (155 mg, 0.4 mmol, 98%) and dry Et_3_N (222 μL, 1.6 mmol) were added. The reaction was stirred at room temperature for 16 h. W-A(triazole)-Pyr **6** (194 mg, 66%) was isolated by preparative chromatography (CH_2_Cl_2_/CH_3_OH 9:1) as a yellow foam. *R*_f_ = 0.82 (CH_2_Cl_2_/CH_3_OH 9:1); ^1^H NMR (DMSO-*d*_6_) *δ*/ppm: 10.88 (s, 1H, NH-Trp), 8.43 (dd, *J* = 7.5, 1.6 Hz, 1H, NH-C=O), 8.34 (d, *J* = 9.2 Hz, 1H, Pyr), 8.27–7.77 (m, 10H, 8H-Pyr, H-5 triazole, NH-C=O), 7.48 and 7.47 (2xd, *J* = 8.0 Hz, 1H, Trp), 7.32 and 7.31 (2xd, *J* = 4.8 Hz, 1H, Trp), 7.18 and 7.13 (2xd, *J* = 2.3 Hz, 1H, Trp), 7.06–7.02 (m, 1H, Trp), 6.97–6.94 (m, 1H, Trp), 6.20 and 6.16 (d, *J* = 1.6 Hz, and brs, 2H, CH_2_), 4.70–4.66 (m, 1H, CH), 4.61–4.46 (m, 1H, CH), 3.55 and 3.54 (2xs, 3H, OCH_3_), 3.29–3.20 (m, 2H, CH_2_), 3.18–3.08 (m, 2H, CH_2_), 3.07–2.76 (m, 2H, CH_2_), 2.29–2.14 (m, 2H, CH_2_), 1.97–1.87 (m, 2H, CH_2_), 0.99 and 0.98 (2xs, 9H, Me_3_C=O); ^13^C NMR (DMSO-*d*_6_) *δ*/ppm: 176.4 and 176.3 (C=O, Piv), 172.1 (NH-C=O, Pyr), 172.0 and 171.9 (MeO-C=O), 171.1 and 170.8 (NH-C=O), 143.7 and 143.68 (C4, triazole), 136.5 (Cq, Pyr), 136.1 and 136.0 (Cq, Trp), 130.9 (Cq, Pyr), 130.4 (Cq, Pyr), 129.3 (Cq, Pyr), 128.1 (Cq, Pyr), 127.5 (CH, Pyr), 127.4 (CH, Pyr), 127.2 (CH, Pyr), 127.0 (Cq, Trp), 126.5 (CH, Pyr), 126.1 (CH, Pyr), 124.89 and 124.87 (C5, triazole), 124.7 (Cq, Pyr), 124.2 (CH, Pyr), 124.1 (CH, Pyr), 123.8 (Cq, Pyr), 123.8 and 123.77 (CH, Trp), 123.7 (CH, Pyr), 123.5 (CH, Pyr), 121.0 (CH, Trp), 118.4 (CH, Trp), 118.0 and 117.9 (CH, Trp), 111.4 (CH, Trp), 109.2 and 109.1 (Cq, Trp), 69.9 (N-CH_2_-O), 53.3 and 53.1 (CH), 51.9 (CH), 51.8 (OCH_3_), 38.2 and 38.1 (Me_3_C-C=O), 34.8 (CH_2_), 32.1 (CH_2_), 28.1 and 28.0 (CH_2_), 27.4 (CH_2_), 27.1 and 26.9 (CH_2_), 26.3 (Me_3_C-C=O); (see Supporting Information Figure S6). HRMS (MALDITOF/TOF): *m*/*z* calcd. for C_43_H_44_N_6_O_6_ [M+Na]^+^: 763,3220; found 763,3229.

4-(3-(((*S*)-3-(1*H*-Indol-3-yl)-1-methoxy-1-oxopropan-2-yl)amino)-3-oxo-2-(pyrene-1-carboxamido)propyl)-1*H*-1,2,3-triazol-1-yl)methyl pivalate (7).

Dipeptide **5** (108 mg, 0.185 mmol) and 1-pyrenecarboxylic acid (47 mg, 0.185 mmol) were dissolved in dry DMF (5 mL) under argon and HOBt (26 mg, 0.185 mmol), HBTU (72 mg, 0.185 mmol) and dry Et_3_N (103 μL, 0.74 mmol) were added. The reaction was stirred at room temperature for 16 h. W-A(triazole)-Pyr **7** (82 mg, 64%) was isolated by preparative chromatography (CH_2_Cl_2_/CH_3_OH 9:1) as a yellow foam. *R*_f_ = 0.80 (CH_2_Cl_2_/CH_3_OH 9:1); ^1^H NMR (DMSO-*d*_6_) *δ*/ppm: 10.92 (s, 1H NH-Trp), 8.87 and 8.83 (2x brd, 1H, NH-C=O), 8.65 and 8.61 (2xbrd, 1H, -NH-C=O), 8.14 (m, 10H, 9H-Pyr+H-5 triazole), 7.54 (d, *J* = 7.9 Hz, 1H, Trp), 7.35 (d, *J* = 8.0 Hz, 1H, Trp), 7.28 and 7.22 (2xd, *J* = 2.2 Hz, 1H, Trp), 7.09–7.06 (m, 1H, Trp), 7.02–6.98 (m, 1H, Trp), 6.35–6.26 (m, 2H, CH_2_), 5.04–4.99 (m, 1H, CH), 4.68–4.64 (m, 1H, CH), 3.65 and 3.62 (2xs, 3H, OCH_3_), 3.24–2.99 (m, 4H, 2xCH_2_), 1.04 and 1.03 (2xs, 9H, Me_3_C=O); ^13^C NMR (DMSO-*d*_6_) *δ*/ppm: 176.5 and 176.47 (C=O, Piv), 172.3 and 172.2 (MeO-C=O), 171.1 and 170.8 (NH-C=O), 168.8 and 168.79 (NH-C=O, Pyr), 143.95 and 143.93 (C4, triazole), 136.14 and 136.11 (Cq, Trp), 131.7 and 131.6 (Cq, Pyr), 131.3 and 131.26 (Cq, Pyr), 130.7 (Cq, Pyr), 130.2 (Cq, Pyr), 128.3 (Cq, Pyr), 128.02 and 128.00 (CH, Pyr), 127.9 (CH, Pyr), 127.2 (CH, Pyr), 127.1 and 127.08 (Cq, Trp), 126.6 (CH, Pyr), 125.8 (CH, Pyr), 125.6 (CH, Pyr), 125.3 and 125.26 (C5, triazole), 124.7 (Cq, Pyr), 124.2 (CH, Pyr), 124.1 and 124.0 (CH, Pyr), 123.9 and 123.8 (CH, Pyr), 123.7 (Cq, Pyr), 123.6 and 123.56 (CH, Trp), 121.0 (CH, Trp), 118.5 (CH, Trp), 118.0 and 117.99 (CH, Trp), 111.5 (CH, Trp), 109.3 (Cq, Trp), 69.91 and 69.90 (N-CH_2_-O), 53.4 and 53.1 (CH), 53.1 and 52.9 (CH), 52.0 and 51.9 (OCH_3_), 38.14 and 38.13 (Me_3_C-C=O), 27.8 and 27.7 (CH_2_), 27.2 and 26.97 (CH_2_), 26.4 and 26.38 (Me_3_C-C=O); (see Supporting Information Figure S7). HRMS (MALDI-TOF/TOF): *m*/*z* calcd. for C_40_H_38_N_6_O_6_Na^+^ [M+Na]^+^: 721,2751; found 721,2763.

ethyl (6*S*,12*S*)-6,12-bis((1*H*-indol-3-yl)methyl)-2,2-dimethyl-4,7,10-trioxo-9-((1-((pivaloyloxy)methyl)-1*H*-1,2,3-triazol-4-yl)methyl)-3-oxa-5,8,11-triazatridecan-13-oate (9).

Compound **5** (231mg 0.395 mmol) and Boc-protected tryptophan **8** (120 mg, 0.395 mmol) were dissolved in dry CH_3_CN (15 mL) under argon and HOBt (97%, 54 mg, 0.395 mmol), HBTU (98%, 153 mg, 0.395 mmol) and dry Et_3_N (0.224 mL, 1.58 mmol) were added. The reaction mixture was stirred at room temperature for 16 h. Protected W-A(triazole)-W tripeptide **9** (189 mg, 63%) was isolated by preparative chromatography (CH_2_Cl_2_/CH_3_OH 9:1) as a light yellow powder: *R*_f_ = 0.76 (CH_2_Cl_2_/CH_3_OH 9:1); ^1^H NMR (DMSO-*d*_6_) *δ*/ppm: 10.86 (s, 1H, NH-Trp), 10.76 (s, 1H, NH-Trp), 8.93–8.61 (m, 1H, NH-C=O), 8.52–8.13 (m, 1H, NH-C=O), 7.96 and 7.71 (2xs, 1H, H-5 triazole), 7.61–7.41 (m, 2H, Trp), 7.35–7.27 (m, 2H, Trp), 7.16–6.74 (m, 7H, NH and Trp), 6.24 and 6.14 (2xs, 2H, CH_2_), 4.63 (brs, 1H, CH), 4.58–4.46 (m, 1H, CH), 4.26–4.07 (m, 1H, CH), 3.56 and 3.55 (2xs, 3H, OCH_3_), 3.23–2.83 (m, 6H, 3x CH_2_), 1.28 (brs, 9H, Me_3_C-O-), 1.09 and 1.03 (2xs, 9H, Me_3_C=O); ^13^C NMR (DMSO-*d*_6_) *δ*/ppm: 176.4 and 176.3 (C=O, Piv), 171.9 (MeO-C=O), 170.7 and 170.3 (NH-C=O), 164.6 (NH-C=O), 155.3 (Me_3_C-O-C=O), 143.3 (C4, triazole), 136.0 (Cq, Trp), 135.99 (Cq, Trp), 127.31 and 127.30 (Cq, Trp), 127.0 and 126.97 (Cq, Trp), 124.0 and 123.9 (C5, triazole), 123.8 (CH, Trp), 123.7 and 123.6 (CH, Trp), 120.9 (CH, Trp), 120.7 (CH, Trp), 118.5 (CH, Trp), 118.4 (CH, Trp), 118.1 (CH, Trp), 117.9 (CH, Trp), 111.4 (CH, Trp), 111.1 (CH, Trp), 110.1 (Cq, Trp), 109.1 (Cq, Trp), 78.1 (Cq, Me_3_C-O-), 69.8 (N-CH_2_-O), 55.3 and 55.0 (CH), 53.1 (CH), 51.9 (CH), 51.79 and 51.77 (OCH_3_), 38.2 (Me_3_C-C=O), 31.3 (CH_2_), 28.1 (Me_3_C-O-), 27.5 and 27.4 (CH_2_), 27.2 and 26.9 (CH_2_), 26.4 and 26.37 (Me_3_C-C=O); (see Supporting Information Figure S9). ESI-MS: *m*/*z* calcd. for C_39_H_48_N_8_NaO_8_ [M+Na]^+^ 779.35, found 779.7.

(2*S*)-1-((1-(((*S*)-3-(1*H*-indol-3-yl)-1-methoxy-1-oxopropan-2-yl)amino)-1-oxo-3-(1-((pivaloyloxy)methyl)-1*H*-1,2,3-triazol-4-yl)propan-2-yl)amino)-3-(1*H*-indol-3-yl)-1-oxopropan-2-aminium 2,2,2-trifluoroacetate (10).

Compound **9** (42 mg, 0.24 mmol) was dissolved in 1:1 mixture of TFA/CH_2_Cl_2_ (1.2 mL) and stirred at room temperature for 20 h. After removal of the remaining TFA under reduced pressure, W-A(triazole)-W tripeptide **10** (41 mg, 95%) was obtained as a dark blue solid. *R*_f_ = 0.62 (CH_2_Cl_2_/CH_3_OH 9:1); ^1^H NMR (DMSO-*d*_6_) *δ*/ppm: 10.97 (brs, 1H, NH-Trp), 10.89 (brs, 1H, NH-Trp), 8.95–8.67 (m, 2H, 2x NH-C=O), 8.02–7.88 (m, 4H, NH_3_^+^ and H-5 triazole), 7.73–7.66 (m, 2H, Trp), 7.51 (t, *J* = 7.6 Hz, 1H, Trp), 7.36–7.32 (m, 2H, Trp), 7.21–713 (m, 1H, Trp), 7.11–6.94 (m, 4H, Trp), 6.26 and 6.14 (2xs, 2H, CH_2_), 4.80–4.37 (m, 2H, 2x CH) 4.12–3.89 (m, 1H, CH), 3.58 and 3.56 (2xs, 3H, OCH_3_), 3.29–2.88 (m, 6H, 3x CH_2_), 1.11 and 1.01 (2xs, 9H, Me_3_C=O); ^13^C NMR (DMSO-*d*_6_) *δ*/ppm: 176.5 and 176.4 (C=O, Piv), 172.2 and 172.0 (MeO-C=O), 170.3 and 170.1 (NH-C=O), 168.5 and 168.4 (NH-C=O), 159.5, 158.4, and 157.9 (CF_3_-C=O), 143.0 and 142.9 (C4, triazole), 136.3 (Cq, Trp), 136.1 (Cq, Trp), 127.03 and 127.00 (Cq, Trp), 125.1 and 125.0 (Cq, Trp), 124.3 (C5, triazole), 124.2 (C5, triazole), 124.0 (CH, Trp), 123.9 (CH, Trp), 121.1 (CH, Trp), 121.0 (CH, Trp), 118.4 (CH, Trp), 118.3 (CH, Trp), 118.2 (CH, Trp), 118.0 (CH, Trp), 111.5 (CH, Trp), 111.4 (CH, Trp), 109.2 (Cq, Trp), 106.8 (Cq, Trp), 69.9 (N-CH_2_-O), 69.8 (N-CH_2_-O), 52.4 (CH), 52.3 (CH), 52.0 (CH), 51.9 and 51.8 (OCH_3_), 38.2 (Me_3_C-C=O), 38.1 (Me_3_C-C=O), 31.9 and 31.7 (CH_2_), 30.7 (CH_2_), 29.7 (CH_2_), 26.5 and 26.4 (Me_3_C-C=O); (see Supporting Information Figure S10). HRMS (MALDI-TOF/TOF): *m*/*z* calcd. for C_34_H_41_N_8_O_6_^+^ [M^+^] 657.3149, found 657.3124.

### 3.3. Spectrophotometric Characterisation

Absorption spectra were recorded on a Varian Cary 100 Bio spectrophotometer at room temperature. Fluorescence measurements were performed on an Agilent Cary Eclipse fluorometer, taking care that, at the excitation wavelength, the absorbance was > 0.05. Fluorescence decays were measured by a time-correlated single-photon counting (TC-SPC) Edinburgh FS5 spectrometer, in a previously degassed aqueous solution of dye. Absolute quantum yields of fluorescence (Φ_f_) were measured at the room temperature (25 °C) in sodium cacodylate buffer, pH = 7.0, I = 0.05 M, by the Integrating sphere SC-30 (Edinburgh FS5 Inst.). For all spectrophotometric measurements, a quartz cuvette of 10 mm path length was used.

### 3.4. Study of DNA/RNA Interactions

All measurements were performed in aqueous buffer solution (pH = 7.0, *I* = 0.05 M, sodium cacodylate buffer). The UV-Vis spectra were recorded on a Varian Cary 100 Bio spectrometer, fluorescence spectra were recorded on a Varian Cary Eclipse fluorimeter and CD spectra were recorded on a JASCO J815 spectropolarimeter at 25.0 °C using appropriate quartz cuvettes (path length: 1 cm).

Polynucleotides were purchased as noted: poly dAdT—poly dAdT, poly dGdC—poly dGdC, poly A—poly U, (Sigma) and *calf thymus* (ct)—DNA (Aldrich), and dissolved in sodium cacodylate buffer, *I* = 0.05 M, pH = 7.0. The ct-DNA was additionally sonicated and filtered through a 0.45 mm filter to obtain mostly short (ca. 100 base pairs) rod-like B-helical DNA fragments [45]. Polynucleotide concentration was determined spectroscopically [46] as the concentration of phosphates (corresponds to *c*(nucleobase)).

Circular dichroism (CD) spectra were recorded on a JASCO J-815 spectropolarimeter at room temperature using 1 cm-path quartz cuvettes with a scanning speed of 200 nm/min (an average of three accumulations). A buffer background was subtracted from each spectrum. CD experiments were performed by adding portions of the compound stock solution into the solution of the polynucleotide (*c* = 2 × 10^–5^ M).

The DAPI displacement experiment was performed on a Jasco J815 CD spectropolarimeter, in 1.5 mL of sodium cacodylate buffer in a 1 cm-path-length quartz cuvette (pH 7.0, *I* = 0.05 M), range 230–500 nm, high sensitivity of the detector and a scanning speed of 200 nm/min (an average of three accumulations). First, the CD spectrum of the AT-DNA solution (*c* = 2 × 10^−5^ M) was recorded, and then DAPI (*r*[DAPI]/DNA] = 0.6) was added, yielding a strong positive ICD band at 375 nm. Gradually, the studied compound was added for ratios *r* = [compd]/[DNA] and CD spectra were collected, monitoring a decrease in the ICD band at 375 nm.[50] The percentage of DAPI displaced by the compound was calculated, given IDA50% values representing the ratio *r* = [DAPI]/[compound] at which 50% of the ICD375nm band is decreased with respect to the zero baseline of free DNA.

Thermal melting curves for ds-DNA, ds-RNA and their complexes with the studied compounds were determined as previously described [52], by following the absorption change at 260 nm as a function of temperature. The absorbance of the ligands was subtracted from every curve and the absorbance scale was normalized. *T_m_* values are the midpoints of the transition curves determined from the maximum of the first derivative and checked graphically by the tangent method. The Δ*T_m_* values were calculated by subtracting the *T_m_* of the free nucleic acid from the *T_m_* of the complex. Every Δ*T_m_* value here reported was the average of at least two measurements. The error in Δ*T_m_* is ±0.5 °C. 

Complexes of **6** and **7** with CuCl_2_ were prepared by adding a 5-fold excess of CuCl_2_ to the 5 × 10^−6^ M of peptide in 1 mL of the sodium cacodylate buffer (pH = 7.0). After 1 min. of the incubation period (to ensure complex formation), an aliquot of DNA or RNA was added, and the thermal denaturation experiment was performed immediately. It should be stressed that, in experiments with Cu^2+^-peptide complexes to referent DNA/RNA solutions, identical amounts of DMSO and Cu^2+^ were added and so the obtained referent denaturation curves for DNA or RNA were used for the calculation of ∆*Tm* values caused by the Cu^2+^-peptide complexes.

### 3.5. Plasmid Electrophoresis

Sample preparation. For the plasmid electrophoresis assay, compounds **6** and **7** were dissolved in mili-Q water with 10% ethanol to obtain a stock solution of 1 mM. The working solution of 30 µM (prepared in Na cacodylate buffer, pH = 7.0) or 10 µM (prepared in PBS, pH = 7.4) was freshly prepared for purposes of assessing the photoinduced DNA nuclease activity or investigating the influence of Cu^2+^ and thermal-dependent plasmid DNA linearization, respectively. For both experiments, 1 μg of pUC19 plasmid DNA (2686 pb) was incubated with the compounds. To investigate the influence of Cu^2+^, 10 µM of solution of **6** or **7** was mixed with 1 mM CuCl_2_ (dissolved in mili-Q water) in a 1:3 ratio, respectively.

#### 3.5.1. Photoinduced DNA Nuclease Activity of Compounds **6** and **7**

Plasmid DNA with or without the compounds was exposed to UV light for 30 s using a xenon arc lamp (Oriel, Stratford, CT, USA). Samples were run on 0.6% agarose gel and stained with Midori Green DNA binding dye (Nippon Genetics, Europe), together with samples containing compounds and plasmid DNA that were not irradiated, which served as a control for assessing the photoinduced nuclease activity of a certain compound. Plasmid incubated with restriction enzyme served as a control for identifying the linear form of pUC19. Moreover, plasmid without compound was irradiated with UV light in the same conditions to check if UV light itself induced changes in the plasmid DNA forms (linear and supercoiled circular form). After gel electrophoresis, plasmid DNA forms were visualized on a UVITEC Imager (Cleaver Scientific, Rugby, UK).

#### 3.5.2. Thermal-Dependent Plasmid DNA Linearization

Plasmid DNA with or without the compounds, peptidoid/Cu^2+^ complexes or Cu^2+^ itself was incubated at room temperature, at 37 or at 50 °C for 24 h. Plasmid incubated with restriction enzyme served as a control for identifying a linear form of pUC19. Samples were run on 0.6% agarose gel stained with Midori Green DNA binding dye (Nippon Genetics, Europe). After gel electrophoresis, the plasmid DNA forms were visualized on a UVITEC Imager (Cleaver Scientific, Rugby, UK).

### 3.6. Biology

Cells: A549 (human lung carcinoma; ATCC CCL-185) were obtained from the ATCC Cell Biology Collection and were cultured according to the manufacturer’s instructions. Cells were grown in Dulbecco Modified Eagle’s Medium (DMEM, Sigma Aldrich, St. Louis, MI, USA) supplemented with 10% of fetal bovine serum (FBS, Sigma Aldrich, St. Louis, MI, USA) at 37 °C and 5% CO_2_ in a humified atmosphere. Three biological replicas were performed for all experiments.

Cytotoxicity assay; MTT: Studied compounds were dissolved in the appropriate volume of dimethyl sulfoxide solution (DMSO) under sterile conditions to obtain the stock of 10 mM solution and kept in the dark at +4 °C. Before each assay, a new fresh working solution was prepared from the stock solution by diluting it with DMEM. Cells were seeded on a 96-well plate at a concentration of 7 × 10^3^ cells/well in 100 μL of DMEM (10% FBS) and left in the incubator overnight (37 °C, 5% CO_2_). The next day, 100 μL of the working solution was added to the wells; thus, the final concentration of tested compounds was obtained in a total volume of 200 μL/well. All conditions were tested in quadruplicates. Cells treated with the same dilutions of DMSO represented controls, while cells treated only with DMEM (10% FBS) represented negative controls. The plate was then incubated for the next 72 h (37 °C, 5% CO_2_). After the incubation, the medium was removed and 40 μL of MTT solution was added to each well. The plate was incubated in the cell incubator for 3 h, allowing the formazan crystals to form. After 3 h, 170 μL of DMSO was added to each well and put on a shaker for 20 min, allowing crystals to dissolve. The absorbance of the MTT–formazan product was measured with a microplate reader at 600 nm. The absorbance value directly correlated with a cell survival.

For the UV-A irradiation experiments, cell culture plates prepared as above were treated with the studied compounds and irradiated in a Luzchem reactor with eight overhead-mounted LZC-UVA lamps (in total, 64W, 315–400 nm, dose 50.6 mw ∗ m^−2^), with an ~18 cm lamp-to-cell-plate distance, at 90 min, 24 h and 48 h after treatment. Exposure to light was 30 min per day.

Live cell imaging: Live imaging of the cells treated with compounds was performed on the A549 cell line. The cells were seeded in Ibidi imaging cell chambers (Ibidi^®^, Gräfelfing, Germany) in 500 μL of the medium, with a concentration of 5 × 10^4^ cells/well, and left in the cell incubator for 48 h (37 °C, 5% CO_2_). After two days, the cells were treated with a 10 μM solution of a tested compound and left in the cell incubator for 90 min to allow the compound to enter the cells. For analysing co-localization with mitochondria or lysosomes, after incubation with the tested compounds, the cells were rinsed and incubated with 100 nM MitoTracker Deep Red or LysoTracker Deep Red solution (Invitrogen, Molecular Probes), respectively. The cells were incubated for 20 min at 37 °C, allowing the MitoTracker or LysoTracker dye to enter the cells. After incubation, the medium was replaced with 500 μL of fresh DMEM and the cells were immediately observed by a Leica SP8 X confocal microscope (Leica Microsystems, Wetzlar/Mannheim, Wetzlar, Germany). The probability of co-localization was expressed by using the Pearson correlation coefficient. Analysis was performed by ImageJ software and the appropriate JACoP plugin (https://doi.org/10.1111/j.1365-2818.2006.01706.x, 13 June 2022). The Pearson correlation coefficient, r, can take a range of values from +1 to −1. A value of 0 indicates that there is no association between the two variables. A value greater than 0 indicates a positive association; that is, as the value of one variable increases, so does the value of the other variable. A value less than 0 indicates a negative association; that is, as the value of one variable increases, the value of the other variable decreases.

## 4. Conclusions

The here-presented results demonstrated that replacement of the central histidine tryptophan—histidine—tryptophan (WHW) peptide by Ala-(triazole) did not deteriorate the Cu^2+^-cation-binding properties of the peptide. Moreover, when one of the tryptophans was replaced by pyrene alkyl-substituents, the binding affinity toward the Cu^2+^ cation increased by an order of magnitude, reaching the submicromolar range. Such an affinity increase could be attributed to the intramolecular exciplex formation between pyrene and tryptophan, which pre-folded the peptidoid conformation conveniently for Cu^2+^ cation inclusion.

Due to the exciplex formation with Trp fluorophore, the pyrene analogues **6** and **7** showed bathochromically shifted absorbance, reaching into the visible range (about 400 nm), and, even more importantly, strong bathochromically shifted fluorescence at 480–500 nm. This property allowed for the performance of confocal microscopy imaging on living cells in the visible light range, avoiding conditions of strong cellular autofluorescence. 

The studied peptidoids strongly bind to ds-DNA and ds-RNA, particularly the pyrene analogues with micromolar affinity, by insertion within the DNA minor groove or RNA major groove. The peptidoid/Cu^2+^ complexes exhibit distinctively different interactions with ds-DNA/RNA in comparison to the metal-free analogues, particularly in the stabilization of ds-DNA against thermal denaturation. 

The pyrene peptidoids efficiently enter living cells with no apparent cytotoxic effect, the intracellular confocal microscopy imaging showing their accumulation in cytoplasmic organelles. However, irradiation with light (350–400 nm) resulted in a strong antiproliferative effect on cells treated with micromolar concentrations of the pyrene analogues, presumably attributed to pyrene-induced production of singlet oxygen and consecutive cellular damage. 

Thus, these particular pyrene analogues, combining fluorescent emission with photo-induced bioactivity, can be considered promising lead compounds for the development of photo-induced peptide-based theranostic agents [34], particularly in PDT therapy under two-photon absorption (TPA) [39,40,41,42,43] conditions. The obtained results, as well as very recently published advances in pyrene-carrying peptide vectors for detection of free radicals in cell organelles [55], strongly support further biological studies focused on the exact intracellular location of the pyrene peptidoids **6** and **7**, as well as their new analogues, and study of the mechanism of action using analysis of photo-induced intracellular ROS species.

## Figures and Tables

**Table 1:** Electronic absorption and emission data of **10** and its referent ^a^ **Trp** [31], **6** and its referent 1-pyrene butyric acid (**A** [31] and **7** and its referent 1-pyrene carboxylic acid (**B** [31]).

Compd.	λ_max_/nm	*ε*/M^−1^cm^−1^	Φ_f_	^c^ λ_exc_/nm	λ_em_/nm	*τ*/ns ^b^ (Purged)	χ^2^
**A_(ref6)_**	342	62,596+/−1248	^a^ 0.15 ± 0.02	340	398	2.5 (1%) 100.3 (99.2%)	1.060
**B_(ref7)_**	341	31,586+/−1297	^a^ 0.16 ± 0.03	340	401	38.3 (-) 566.2(-B)	1.207
**6**	350	25,012+/−638	^d^ 0.02 ± 0.005	340	472	11.6 (62%) 36.4 (37%)	1.223
**7**	352	15,197+/−225	^d^ 0.08 ± 0.001	340	481	32.2 (100%)	1.286
**^a^ Trp**	295	5600	^a^ 0.14 ± 0.02	280	366	^a^ 0.53 ^a^ 3.1	-
**10**	295	8322+/−0.5	^d^ 0.017 ± 0.002	280	360	0.96 (29%) 4.1 (71%)	1.091

^a^ See ref. [31]; ^b^ Aqueous solutions were purged by argon; ^c^ Samples were excited by pulsing diodes at 340 nm or 280 nm. The measurements were performed three times and the average values are reported. The associated errors correspond to the maximum absolute deviation; ^d^ Absolute fluorescence quantum yield was determined by integrating sphere SC-30, Edinburgh Inst., for argon-purged solutions, by λ_exc_ = 353 nm.

**Table 2:** Binding constants (log *K_s_*) ^a^ and spectroscopic properties of complexes of the conjugates **6**, **7** and **10** with ds-polynucleotides, calculated by processing fluorimetric titrations, performed at pH = 7.0, sodium cacodylate buffer, *I* = 0.05 M.

Compound	ct-DNA	Poly A–Poly U
**6**	6.5	6.8
**7**	6.8	6.6
**10**	4.6	-
**WHW**	4.1	-

^a^ Processing of titration data by means of the Scatchard equation [46] gave values of the ratio *n*[bound dye]/[polynucleotide] = 0.1 and 0.3. For easier comparison, all log *K_s_* values were re-calculated for fixed *n* = 0.2. Correlation coefficients were >0.99 for all calculated values of log *K_s_*.

**Table 3:** Thermal denaturation (^a^ ∆*Tm/*°C) of ct-DNA (*c*(ct-DNA) = 2.5 × 10^−5^ M, r_[(compd.)]/[ct-DNA]_ = 0.25) and poly A poly U (*c*(RNA)= 2.5 × 10^−5^ M, r_[(compd)]/[poly A poly U]_ = 0.25) at pH = 7.0 (sodium cacodylate buffer, *c* = *50* mM) upon addition of **6**, **7** or **10** or addition of pre-prepared complexes of **6**, **7** or **10** with five equivalents of Cu^2+^.

Compound	ct-DNA	AU-RNA	AT-DNA
**6** ^b^**6** + 5Cu^2+^	0 ^c^ −38; +>12	0 +32	0 ^c^ −12; +>12
**7** ^b^**7** + 5Cu^2+^	0 +2.5	0 +1.0	- -
**10** ^b^**10** + 5Cu^2+^	0 +9.8	0 0	- -

^a^ Error in ∆*Tm*: ± 0.5 °C; ^b^ The complex of peptidoids **6, 7** or **10** with 5 equivalents of Cu^2+^ was prepared in cuvettes and, after 1 min. of equilibration, DNA or RNA was added and incubated at room temp. for 2 min. before the thermal denaturation experiment. As a control experiment, DNA/RNA solutions with identical amounts of DMSO and Cu^2+^ were studied and so the obtained denaturation curves were used for the calculation of ∆*Tm* values. ^c^ Biphasic denaturation curves, suggesting that, at a given ratio ***r***, two different types of ds-DNA/compound complexes are present: destabilised ds-DNA (∆*Tm* = −38 °C) and thermally stabilized ds-DNA (∆*Tm* = +>12 °C).

## Data Availability

All data is contained within this manuscript and the Supplementary Materials file.

## Supplementary Materials

The following supporting information can be downloaded at: https://www.mdpi.com/article/10.3390/ijms23137006/s1. References [56, 57, 58, 59, 60, 61, 62, 63, 64, 65] are cited in the supplementary materials.
