# Supplementary material for: Impact of the Histidine-Triazole and Tryptophan-Pyrene Exchange in the WHW Peptide: Cu(II) Binding, DNA/RNA Interactions and Bioactivity
Source: Int J Mol Sci. 2022 Jun 23;23(13):7006. doi: 10.3390/ijms23137006 (PMC9266797; doi:10.3390/ijms23137006)
Supplement: Supplementary file 1 [file ijms-23-07006-s001.zip › ijms-1736983-supplementary.pdf]

International Journal of *Molecular Sciences*

MDPI

Supporting information: Impact of the Histidine-Triazole and Tryptophan-Pyrene Exchange in the WHW Peptide: Cu(II) Binding, DNA/RNA Interactions and Bioactivity

Ivona Krošl [1], Marta Košćak [1], Karla Ribičić [1], Biserka Žinić [1], Dragomira Majhen [2], Ksenija Božinović [2] and Ivo Piantanida [1,*]

[1] Division of Organic Chemistry and Biochemistry, Ruđer Bošković Institute, Bijenička Cesta 54,
10000 Zagreb, Croatia; ivona.krosl@irb.hr (I.K.); marta.koscak@irb.hr (M.K.); karla.ribica@gmail.com (K.R.); biserka.zinic@irb.hr (B.Ž.)
[2] Division of Molecular Biology, Ruđer Bošković Institute, Bijenička Cesta 54, 10000 Zagreb, Croatia;
dragomira.majhen@irb.hr (D.M.); ksenija.bozinovic@irb.hr (K.B.)
* Correspondence: pianta@irb.hr; Tel.: +385-1-4571-326

Contents

1. Synthesis 2
2. Physico-chemical properties of aqueous solutions 20
3. Study of interactions with double-stranded DNA/RNA 29
4. Biology: 41

International Journal of *Molecular Sciences*

MDPI

1. Synthesis

The Boc-protected PG **1** and tryptophan methyl ester hydrochloride **2** were prepared using known methods,[1,2] and subjected to standard coupling reaction using HBTU/HOBt coupling reagents and triethylamine in acetonitrile.[3] The Trp-A(alkyne) dipeptide **3** was isolated in 71% yield. The proton NMR spectrum of the diastereomeric mixture **3** (*D,L* +*L,L*) shows duplicated signals in a 1:1 diastereomeric ratio (see Supporting information Fig. S3b: e.g., two signals for OCH_3 at δ 3.58 and 3.55 ppm with total integration 3H for both signals).

The 1,2,3-triazole ring was synthesized by an *in situ* azidation/cycloaddition protocol[4,5] from Trp-A(alkyne) dipeptide **3** using sodium azide, chloromethyl pivalate, and the copper(II) sulfate/sodium ascorbate system. The protected dipeptide **4** was isolated with an excellent 87 % yield, and after deprotection of the Boc group with TFA/CH_2Cl_2, W-A(triazole) dipeptide **5** was obtained in quantitative yield.

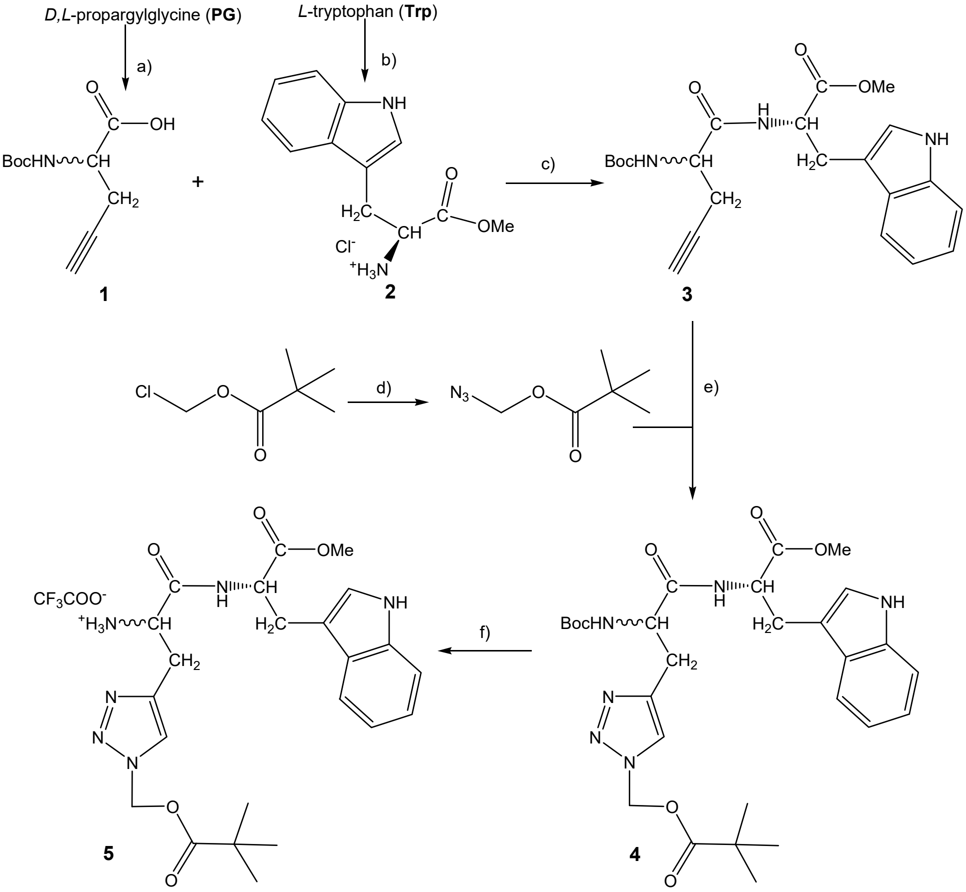

Scheme S1. Synthesis of W-A(triazole) dipeptide **5**: a) Boc_2O, 2M NaOH, dioxane/H_2O; rt 2 h, 97 %; b) Me_3SiCl/MeOH, rt 48 h, 90 %; c) HOBt, HBTU, Et_3N/CH_3CN, rt 16 h, 71 %; d) NaN_3, Na-askorbat, CuI, DMEDA/EtOH,H_2O; 90 ^oC, 1 h; e) Na-askorbat, CuI, DMEDA, 90 ^oC, 30 min, 87 %; f) TFA/CH_2Cl_2 (1:1), 20 h, 100 %.

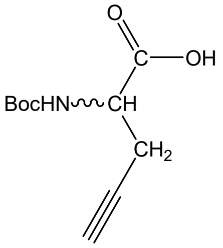

2-((*tert*-Butoxycarbonyl)amino)pent-4-ynoic acid (1) [**Error! Bookmark not defined.**]

D,L-Propargylglycine (PG) (1 g, 8.84 mmol) was dissolved in a solvent mixture of 1,4-dioxane/H_2O (*v*:*v* = 2:1; 18 mL), cooled to 0 °C, and 2 M NaOH solution (4.42 mL, 8.84 mmol) was added dropwise followed by di-*tert*-butyl dicarbonate (2.14 g, 9.81 mmol). The reaction was stirred for 15 min at 0 °C, then allowed to warm to room temperature and stirred for an additional 2 h. The 1,4-dioxane was removed *in vacuo*, and the resulting aqueous residue washed with ethyl acetate (20 mL). The aqueous solution was acidified to pH 2-3 with saturated aqueous $KHSO_4$ solution (7 mL) and extracted with ethyl acetate (3 × 40 mL). The combined organics extracts were washed with water (25 mL) and dried over Na_2SO_4. The solvent was removed *in vacuo* to afford Boc-protected PG **1**, as a light yellow oil which with drying turns to solid (1.838 g, 97 %): R_f = 0.22 (CH_2Cl_2/MeOH = 9:1); ^{1}H NMR (DMSO-d_6) δ/ppm: 12.76 (brs, 1H, COOH), 7.05 (d, *J* = 8.3 Hz, 1H, NH), 4.05 (td, *J* = 8.4, 5.3 Hz, 1H, CH), 2.85 (s, 1H, HC≡C-), 2.70–2.33 (m, 2H, CH_2 covered with DMSO), 1.39 (s, 9H, $\underline{Me}_3$C-O-); ^{13}C NMR (DMSO-d_6) δ/ppm: 172.2 (HO-$\underline{C}$=O), 155.2 (Me_3C-O-$\underline{C}$=O), 80.7 (Cq, $Me_3\underline{C}$-O-), 78.3 (HC≡$\underline{C}$-), 72.8 (H$\underline{C}$≡C-), 52.4 (CH), 28.1 ($\underline{Me}_3$C-O-), 21.1(CH_2); (see Supporting Information Fig. S1).

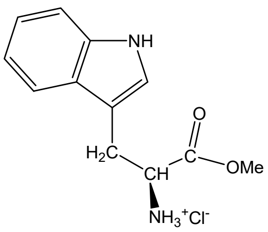

(*S*)-3-(1*H*-Indol-3-yl)-1-methoxy-1-oxopropan-2-aminium chloride (2) [**Error! Bookmark not defined.**]

International Journal of *Molecular Sciences*

MDPI

Freshly distilled chlorotrimethylsilane (2.588 mL, 20.4 mmol) was added slowly to *L*-tryptophan (2.081 g, 10.2 mmol) in a round bottom flask. Then methanol (10 mL) was added and the resulting suspension was stirred at room temperature for 48 h. The solvent was removed *in vacuo* to give the product **2** (2.34 g, 90 %) as a white solid: R_f = 0.65 (CH_2Cl_2/MeOH = 9:1); ^{1}H NMR (DMSO-d_6) δ/ppm: 11.15 (s, 1H, NH-Trp), 8.70 (s, 3H, NH_3^+), 7.52 (d, J = 7.7 Hz, 1H, Trp), 7.37 (d, J = 7.9 Hz, 1H, Trp), 7.26 (d, J = 1.9 Hz, 1H, Trp), 7.08 (t, J = 7.3 Hz, 1H, Trp), 7.00 (t, J = 7.3 Hz, 1H, Trp), 4.19 (t, J = 6.0 Hz, 1H, CH), 3.63 (s, 3H, OCH_3), 3.42–3.27 (m, 2H, CH_2); ^{13}C NMR (DMSO-d_6) δ/ppm: 169.7 (MeO-C=O), 136.2 (Cq, Trp), 126.9 (Cq, Trp), 125.0 (CH, Trp), 121.1 (CH, Trp), 118.6 (CH, Trp), 118.0 (CH, Trp), 111.5 (CH, Trp), 106.3 (Cq, Trp), 52.6 (CH or OCH_3), 52.57 (CH or OCH_3), 26.1 (CH_2); (see Supporting Information Fig. S2).

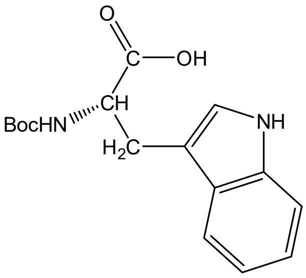

(*tert*-Butoxycarbonyl)-*L*-tryptophan (8) [6]

L-Tryptophan (1 g, 4.8 mmol, 98%) was suspended in 1 M NaOH (4.8 mL), diluted with 3.6 mL of *tert*-butyl alcohol and di-*tert*-butyl dicarbonate (1.237 g 5.28 mmol) was then added in portions over 20 min. The reaction mixture was stirred at room temperature for 15 hours. The solvent was removed *in vacuo* and the resulting thick oil was dissolved in water. The aqueous solution was acidified to pH 1-2 with saturated aqueous $KHSO_4$ solution and extracted with ethyl acetate (3 × 15 mL). The combined organic extracts were washed with water and dried over Na_2SO_4. The solvent was removed *in vacuo* to afford the target material Boc-protected tryptophan **8**, as a white solid (1.184 g, 81 %). ^{1}H NMR (DMSO-d_6) δ/ppm: 12.52 (s, 1H, COOH), 10.82 (s, 1H, NH-Trp), 7.53 (d, J = 7.9 Hz, 1H, Trp), 7.34 (d, J = 8.1 Hz, 1H, Trp), 7.15 (d, J = 1.3 Hz, 1H, Trp), 7.07 (t, J = 7.5 Hz, 1H, Trp), 6.99 (t, J = 7.4 Hz, 1H, Trp), 6.94 (d, J = 8.0 Hz, 1H, NH), 4.16 (td, J = 8.8, 4.8 Hz, 1H, CH), 3.14 (dd, J = 14.6, 4.6 Hz, 1H, Ha-CH_2), 2.99 (dd, J = 14.6, 9.4 Hz, 1H, Hb-CH_2), 1.33 (s, 9H, Me_3C-O-); ^{13}C NMR (DMSO-d_6) δ/ppm: 173.9 (HO-C=O), 155.4 (Me_3C-O-C=O), 136.1 (Cq, Trp), 127.2 (Cq, Trp),

International Journal of
Molecular Sciences

MDPI

123.6 (CH, Trp), 120.9 (CH, Trp), 118.3 (CH, Trp), 118.1 (CH, Trp), 111.4 (CH, Trp), 110.2 (Cq, Trp), 78.0 ($Me_3\underline{C}$-O-), 54.5 (CH), 28.2 ($\underline{Me_3}$C-O-) 26.8 (CH_2); (see Supporting Information Fig. S8).

NMR characterisation:

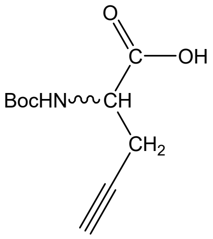

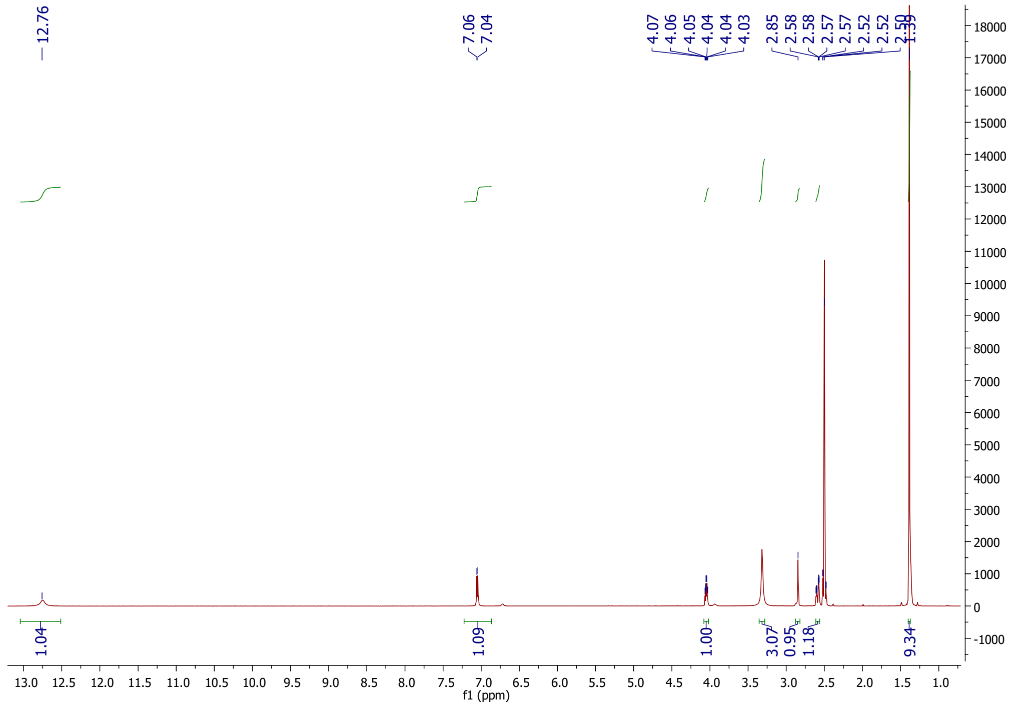

International Journal of
Molecular Sciences

MDPI

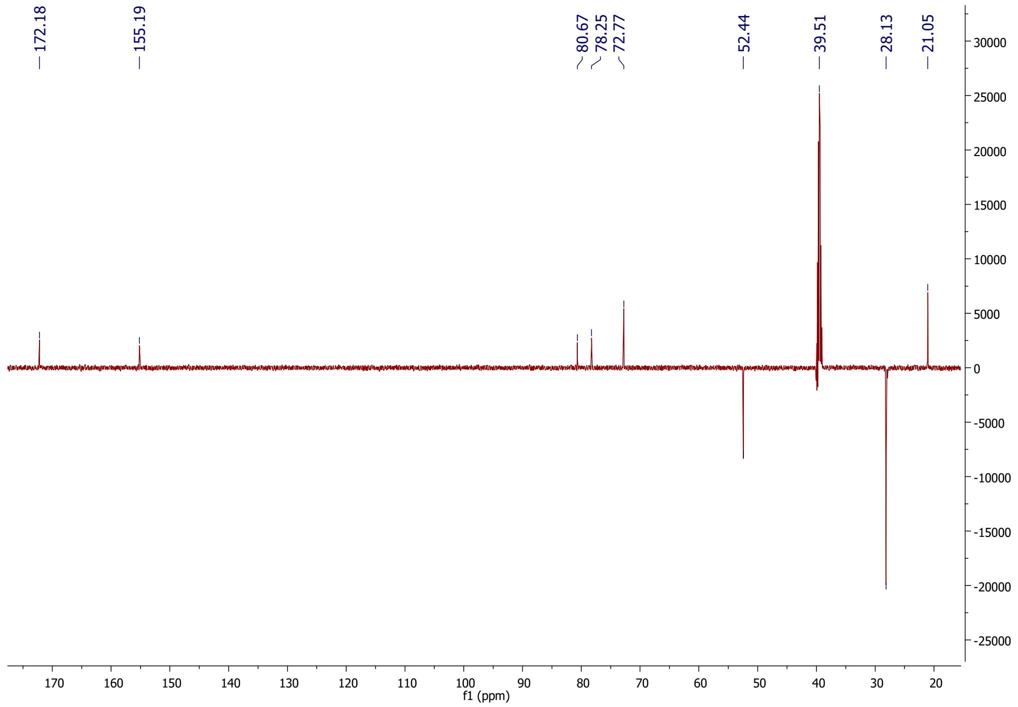

Figure S1. ^{1}H NMR (600 MHz, DMSO-d_6) and ^{13}C NMR (151 MHz, APT, DMSO-d_6) spectra of compound **1**.

International Journal of
Molecular Sciences

MDPI

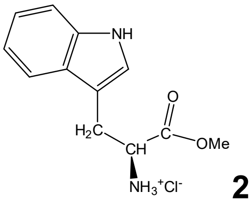

2

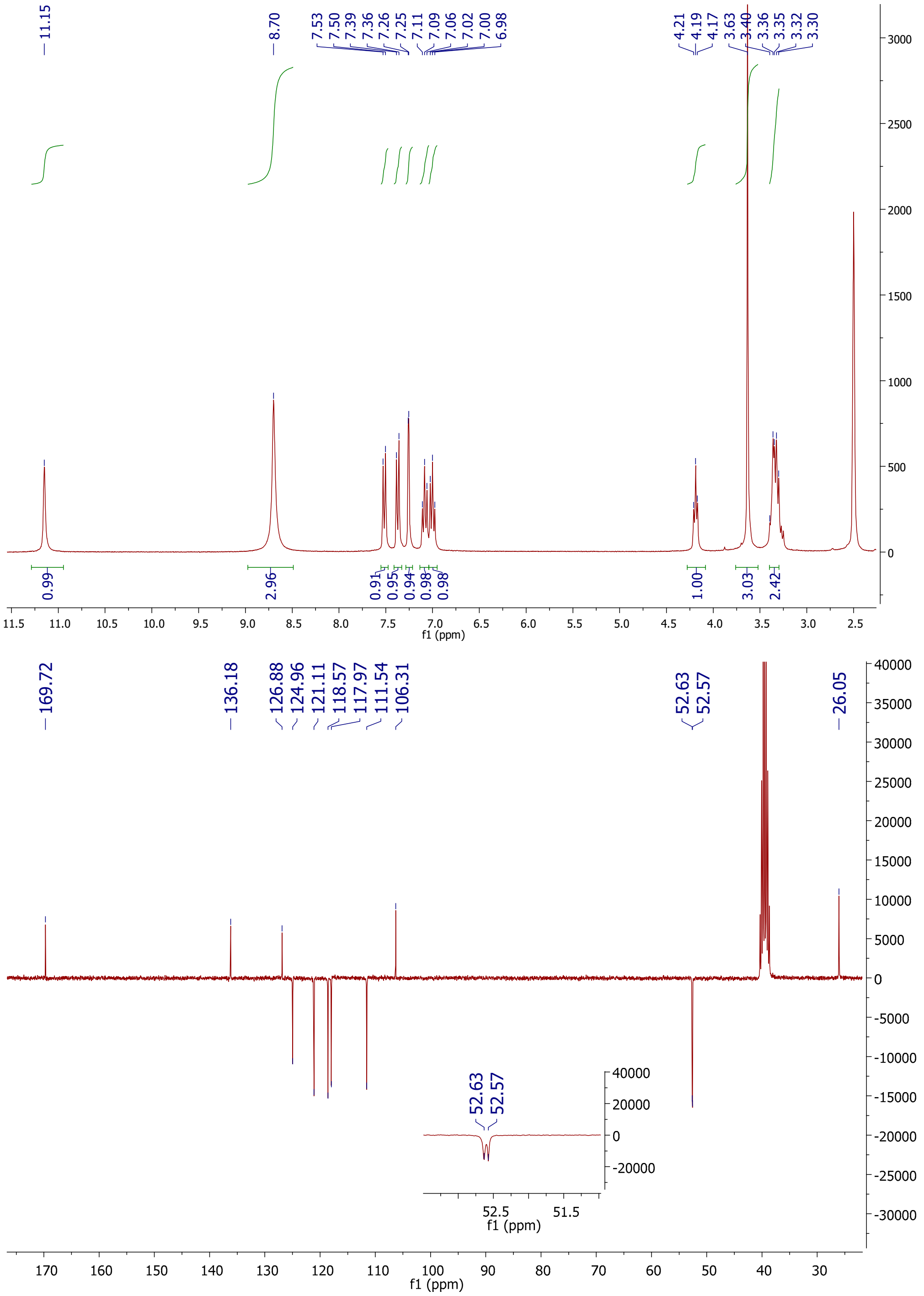

Figure S2. ^{1}H NMR (300 MHz, DMSO-*d*6) and ^{13}C NMR (75 MHz, APT, DMSO-*d*6) spectra of compound **2**.

International Journal of
Molecular Sciences

MDPI

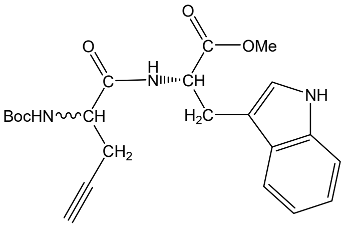

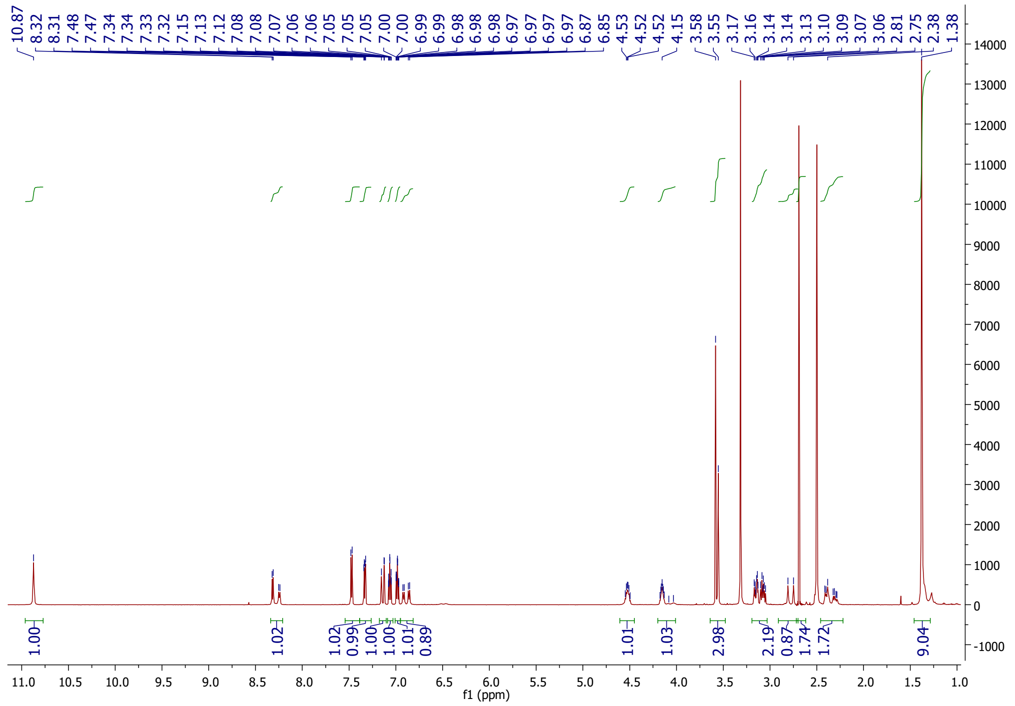

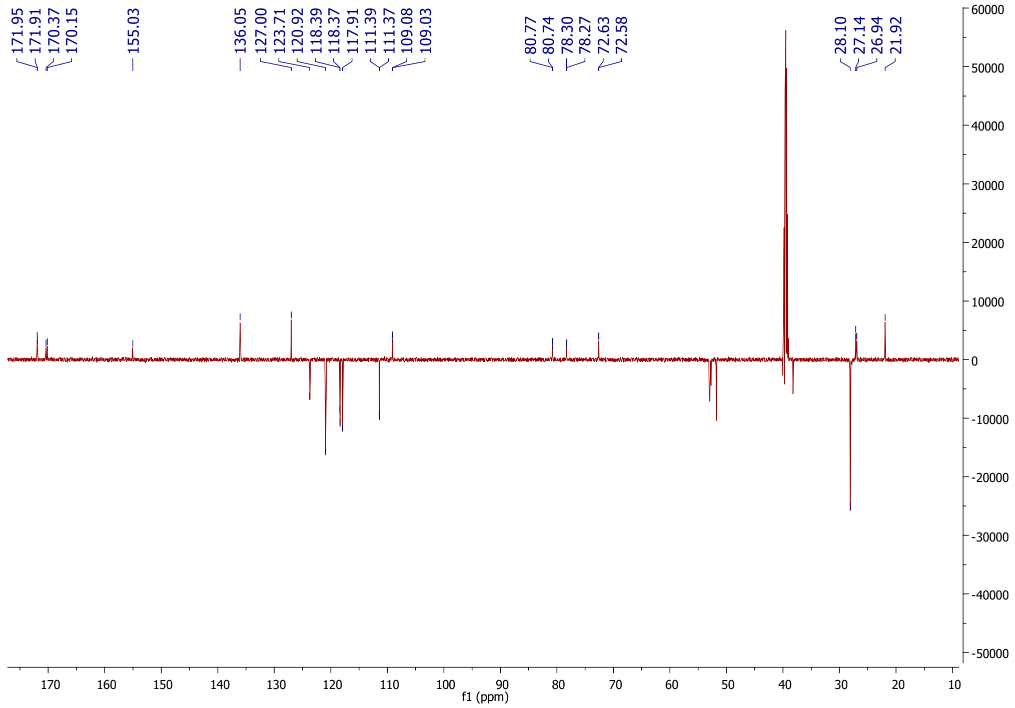

International Journal of *Molecular Sciences*

MDPI

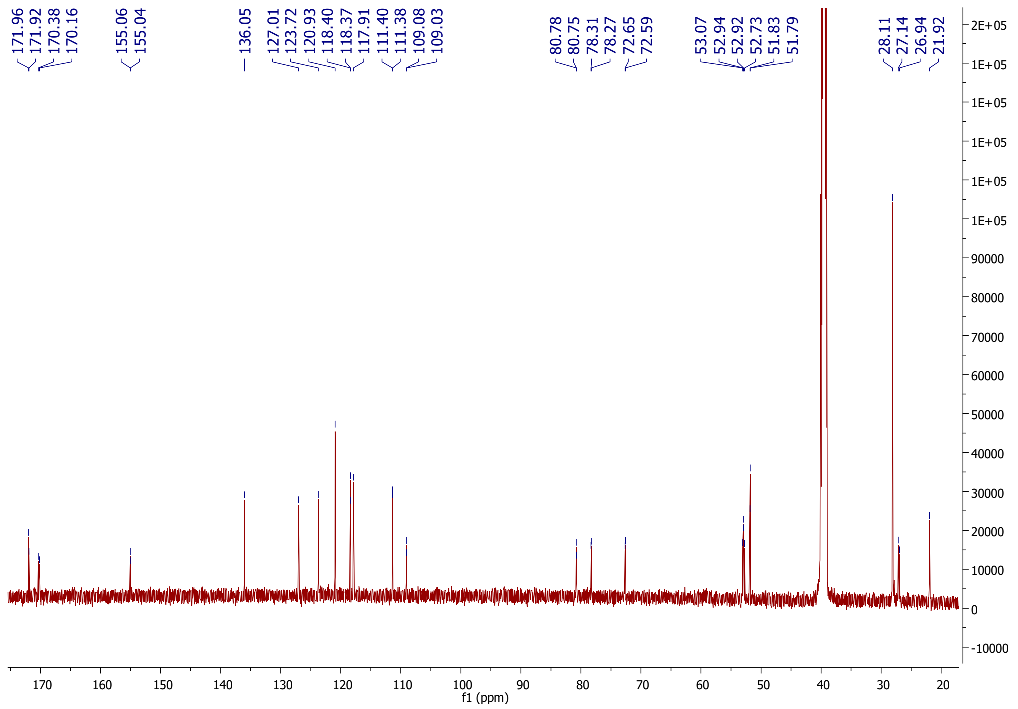

(a)

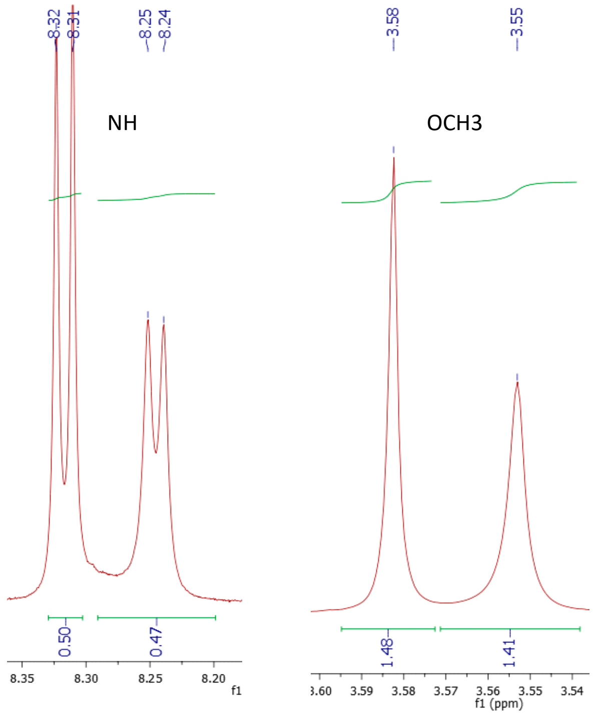

(b)

Figure S3: (a). ^{1}H NMR (600 MHz, DMSO-*d*6) and ^{13}C NMR (151 MHz, DMSO-*d*6) spectra of compound **3**. **(b)**. Part of the ^{1}H NMR (600 MHz, DMSO-*d*6) spectrum of compound **3** for NH and OCH_3 protons.

International Journal of
Molecular Sciences

MDPI

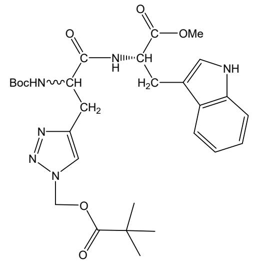

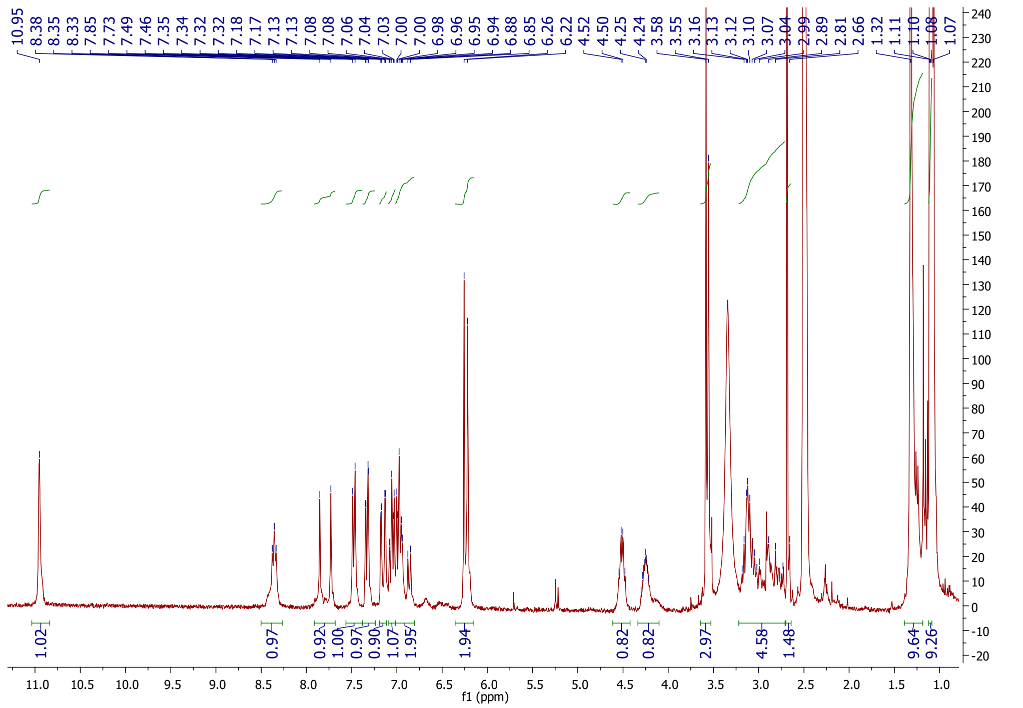

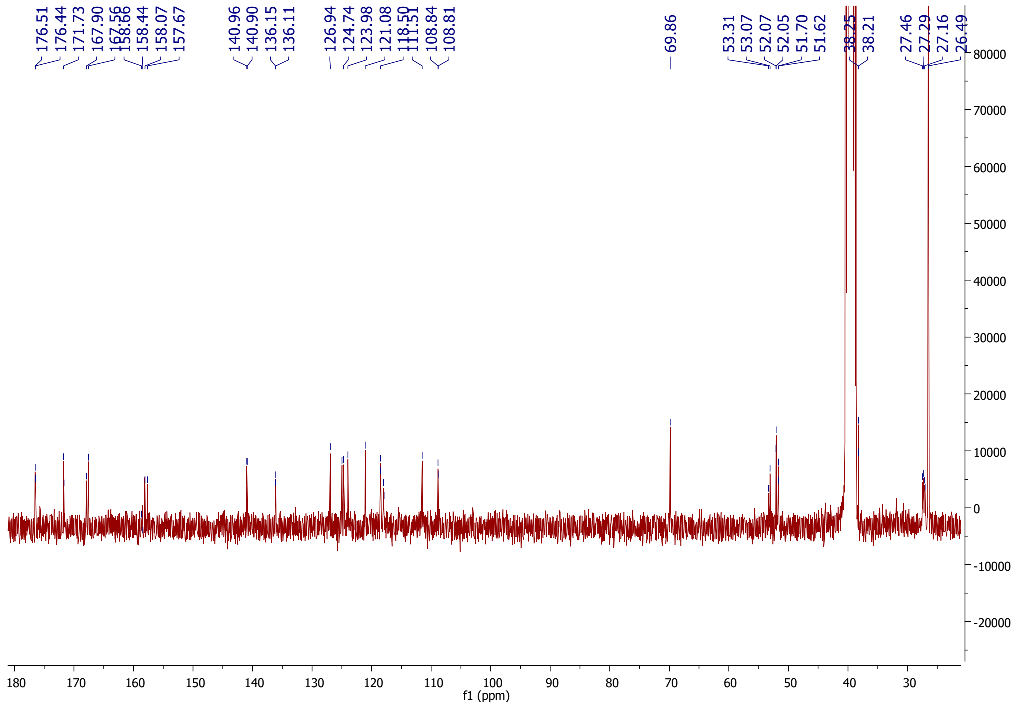

Figure S4. ^{1}H NMR (300 MHz, DMSO-d_6) and ^{13}C NMR (151 MHz, DMSO-d_6) spectra of compound **4**.

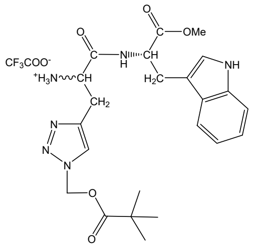

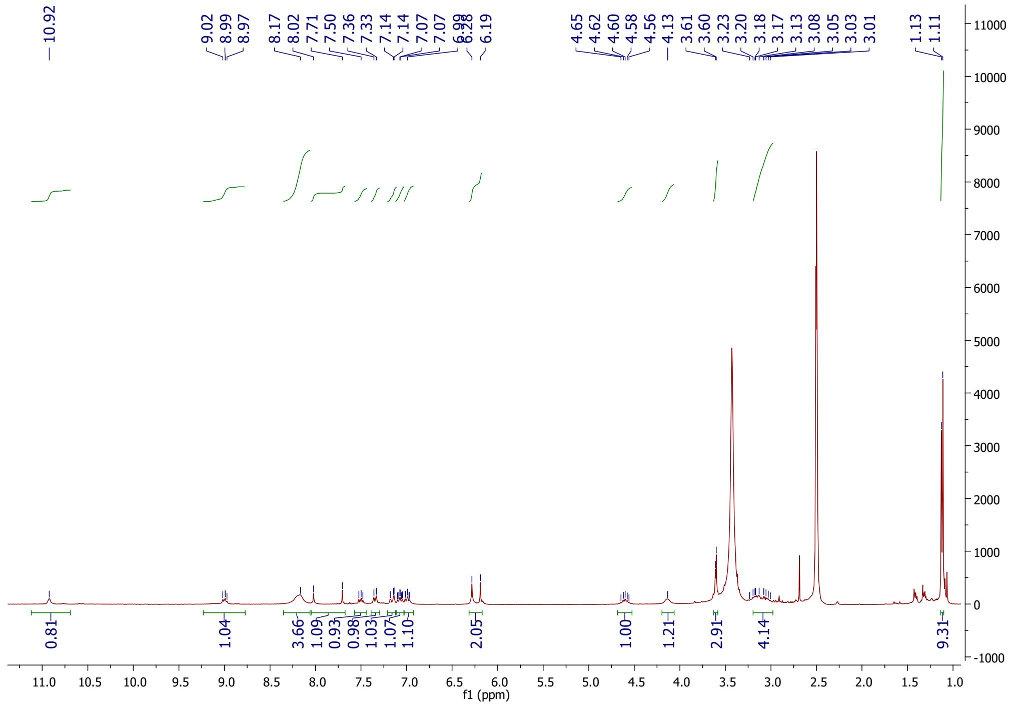

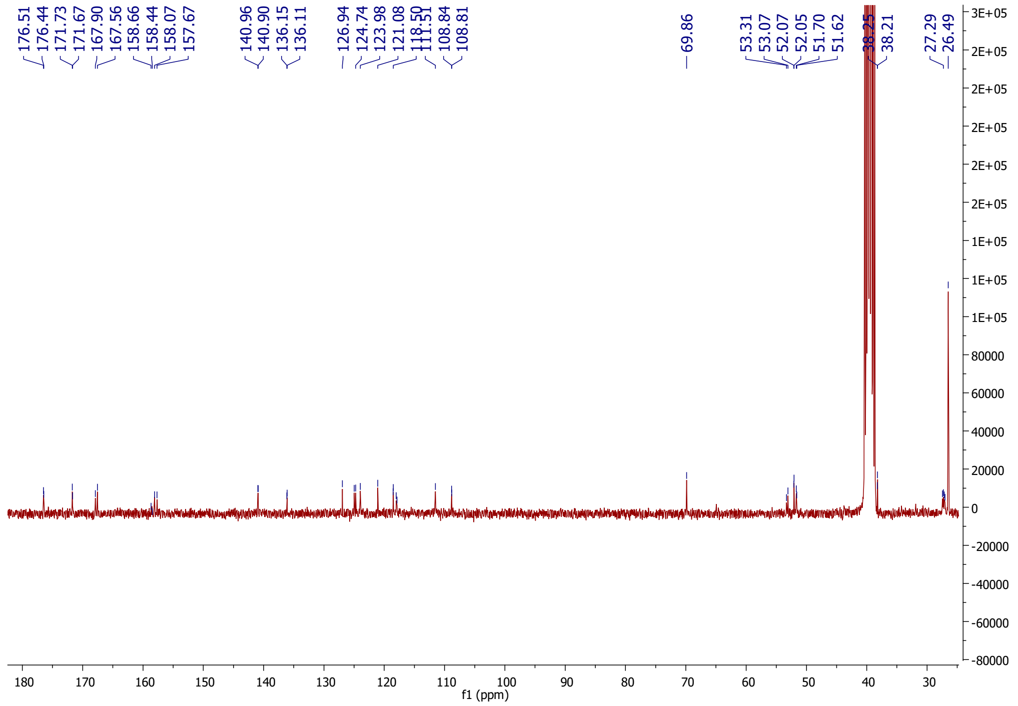

Figure S5. ^{1}H NMR (300 MHz, DMSO-d_6) and ^{13}C NMR (75 MHz, DMSO-d_6) spectra of compound **5**.

International Journal of Molecular Sciences

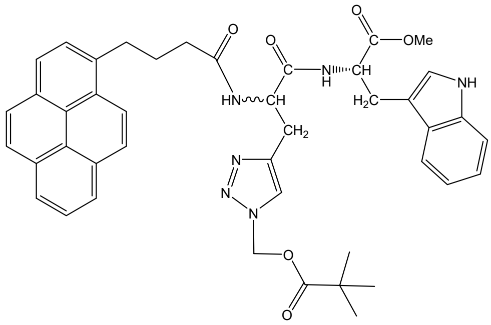

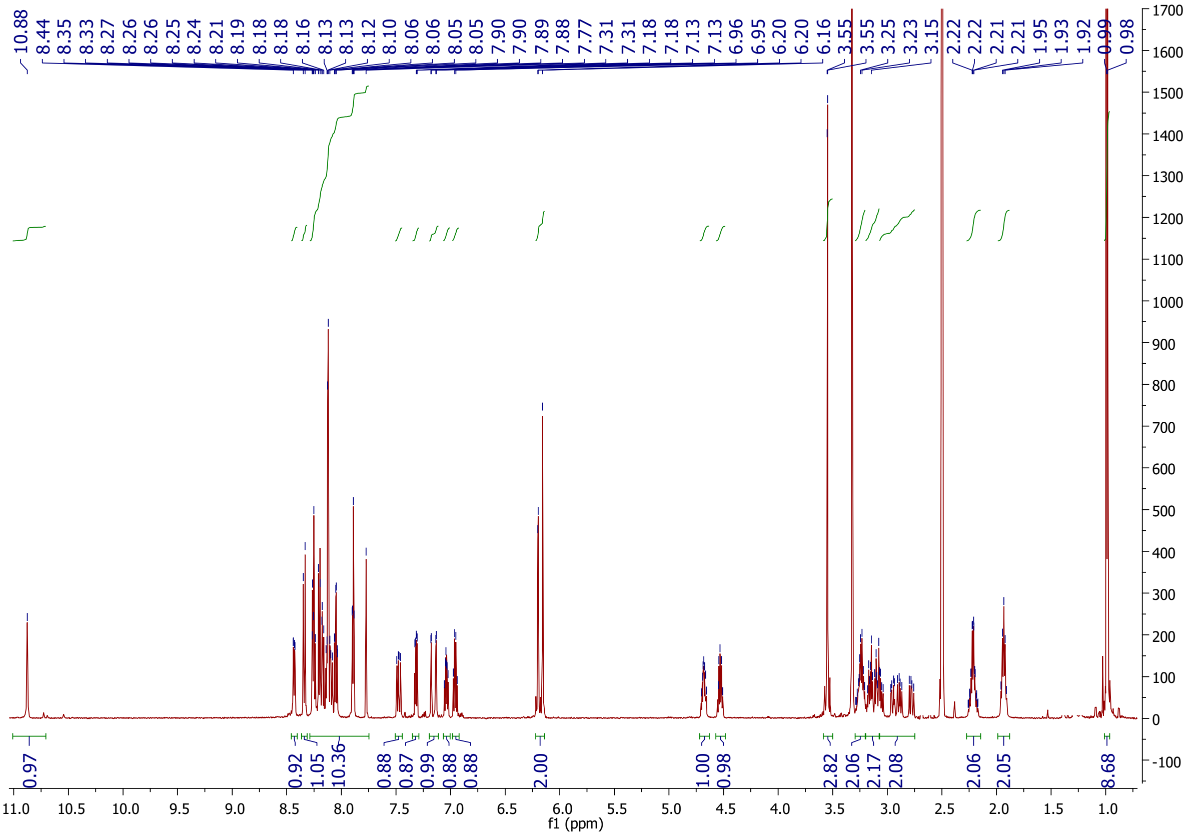

International Journal of
Molecular Sciences

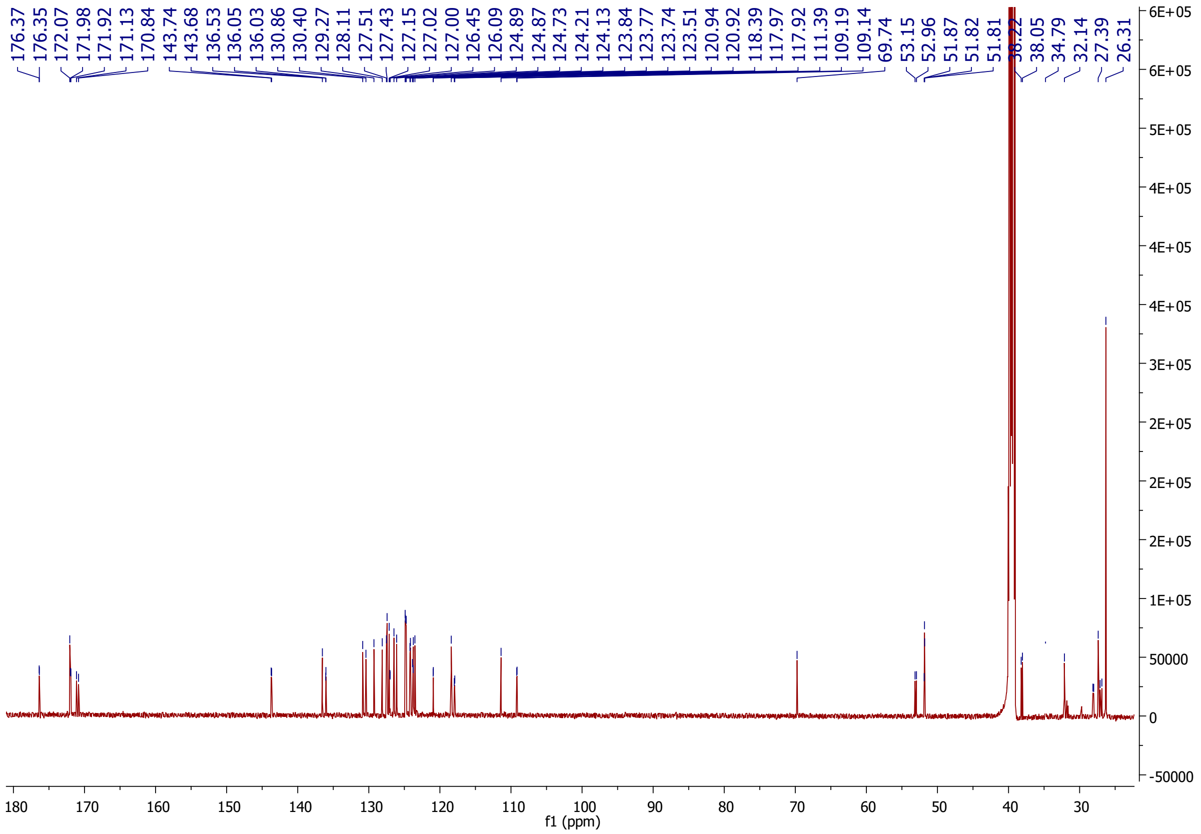

Figure S6. ^{1}H NMR (600 MHz, DMSO-*d*6) and ^{13}C NMR (151 MHz, DMSO-*d*6) spectra of compound **6**.

International Journal of
Molecular Sciences

MDPI

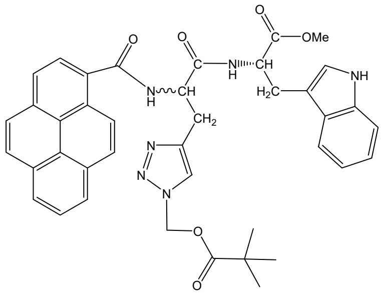

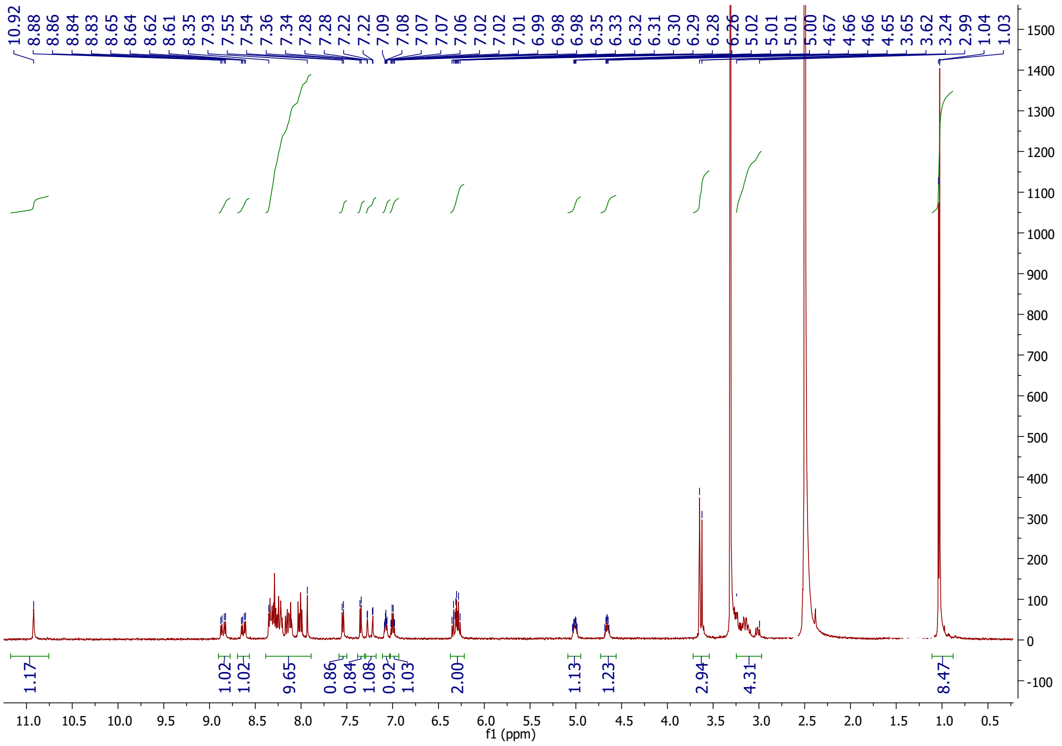

International Journal of
Molecular Sciences

MDPI

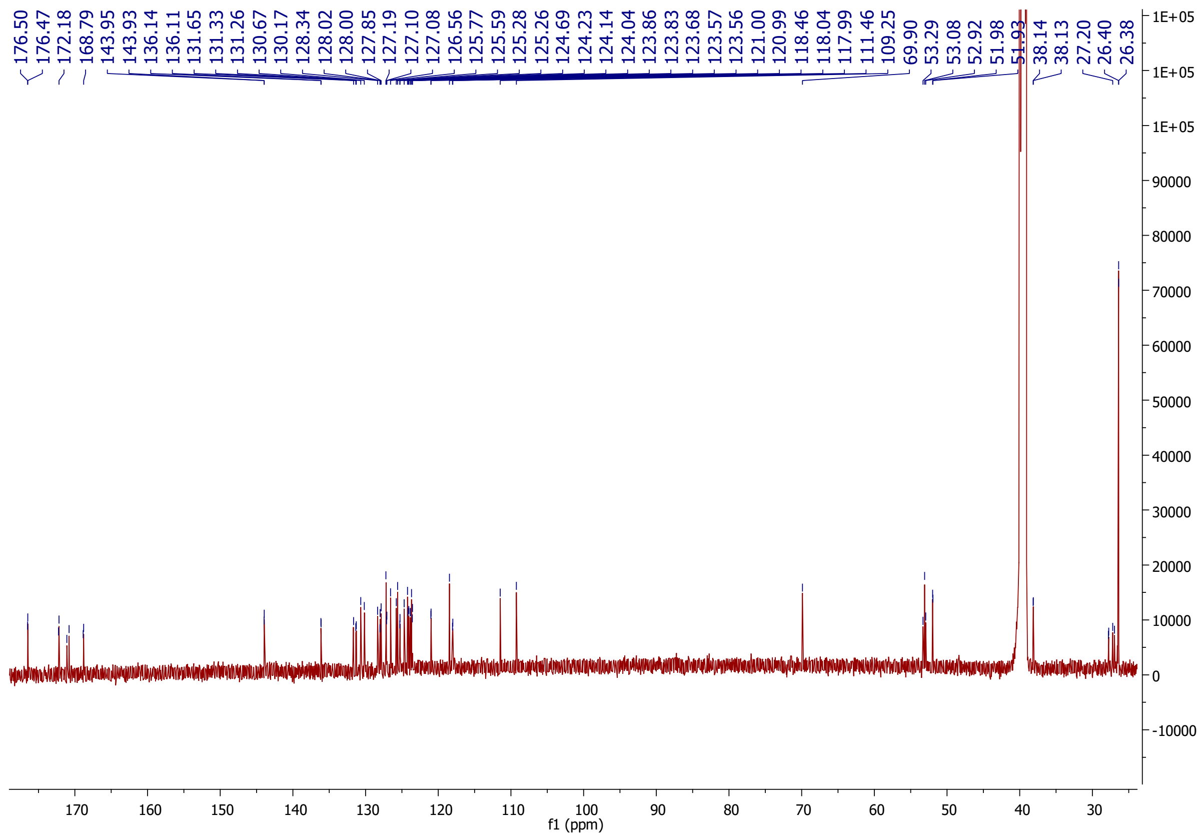

Figure S7. ^{1}H NMR (600 MHz, DMSO-*d*6) and ^{13}C NMR (151 MHz, DMSO-*d*6) spectra of compound **7**.

International Journal of
Molecular Sciences

MDPI

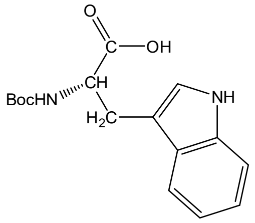

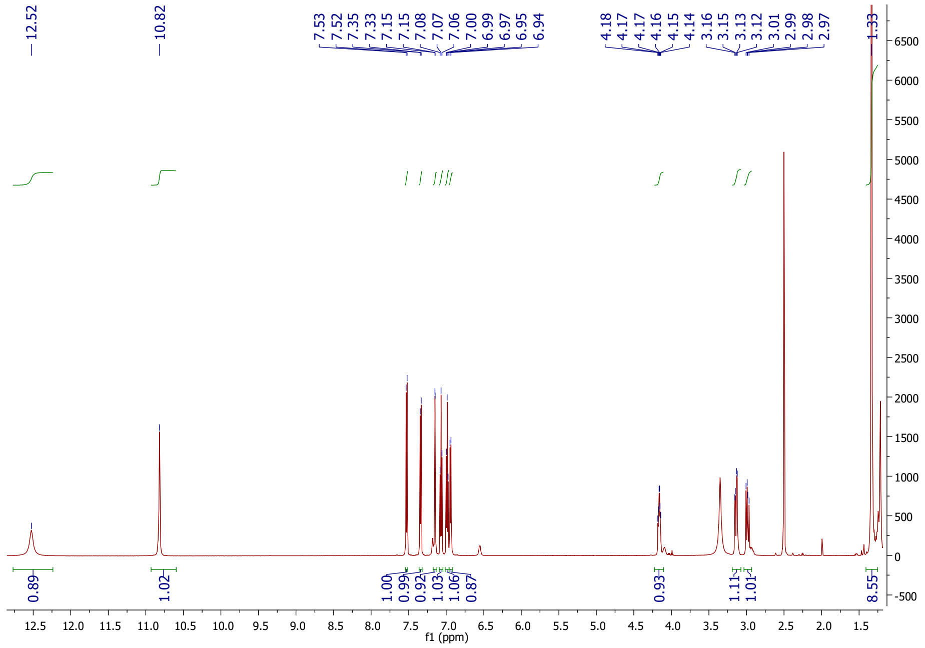

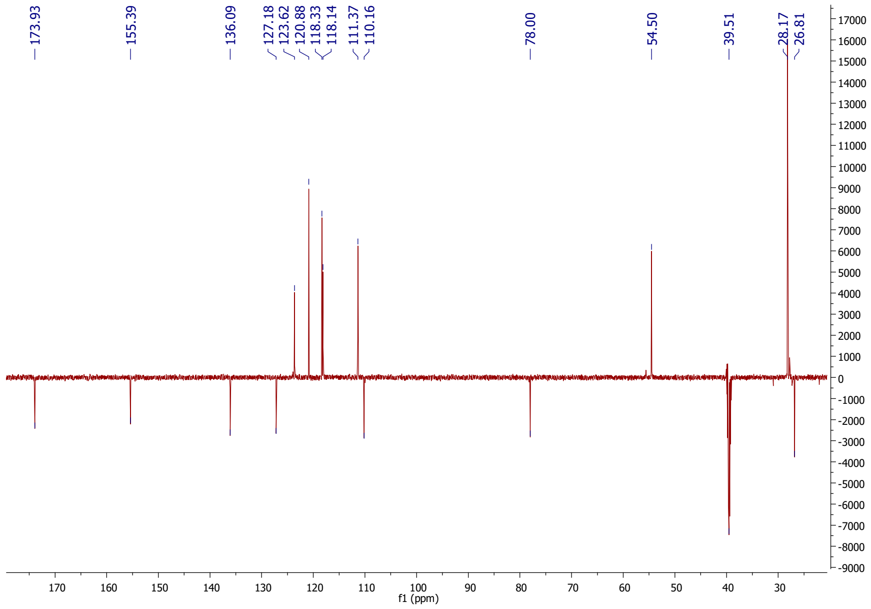

Figure S8. ^{1}H NMR (600 MHz, DMSO-*d*6) and ^{13}C NMR (151 MHz, DMSO-*d*6) spectra of compound **8**.

International Journal of
Molecular Sciences

MDPI

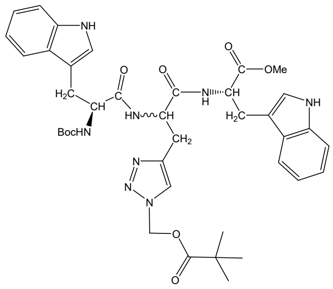

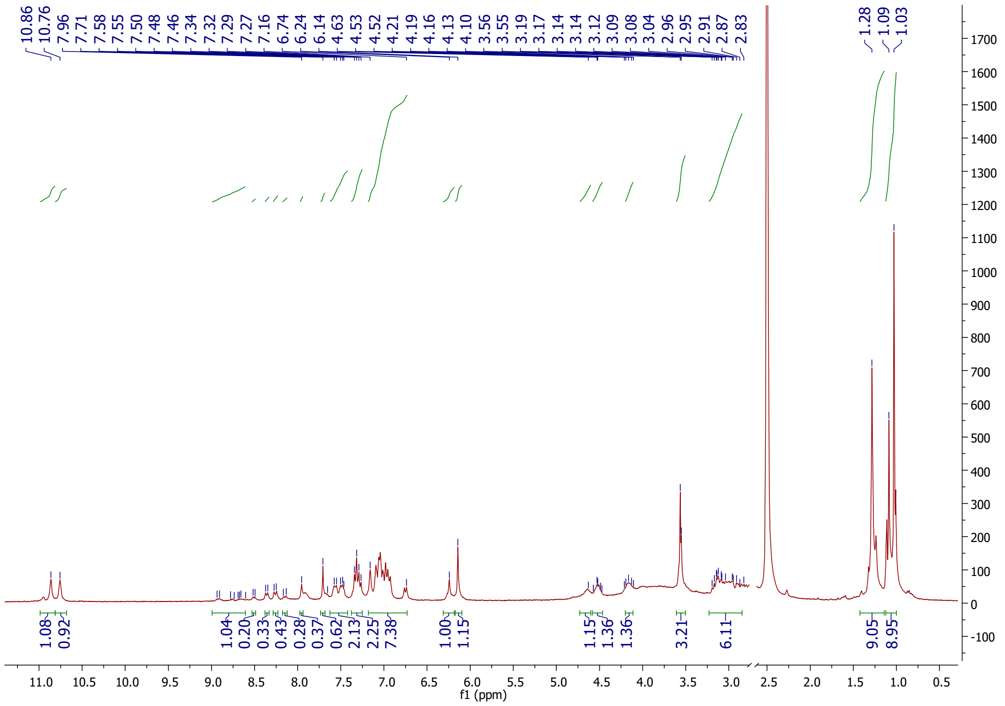

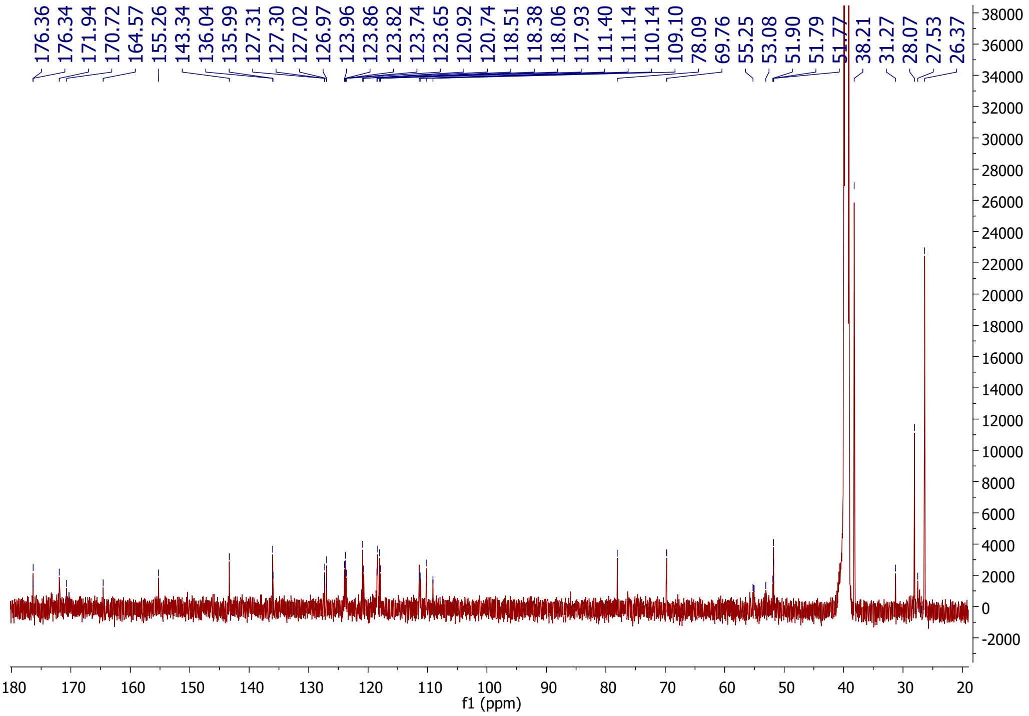

International Journal of Molecular Sciences

MDPI

Figure S9. ^{1}H NMR (300 MHz, DMSO-d_6) and ^{13}C NMR (151 MHz, DMSO-d_6) spectra of compound **9**.

International Journal of *Molecular Sciences*

MDPI

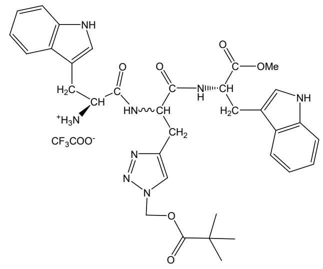

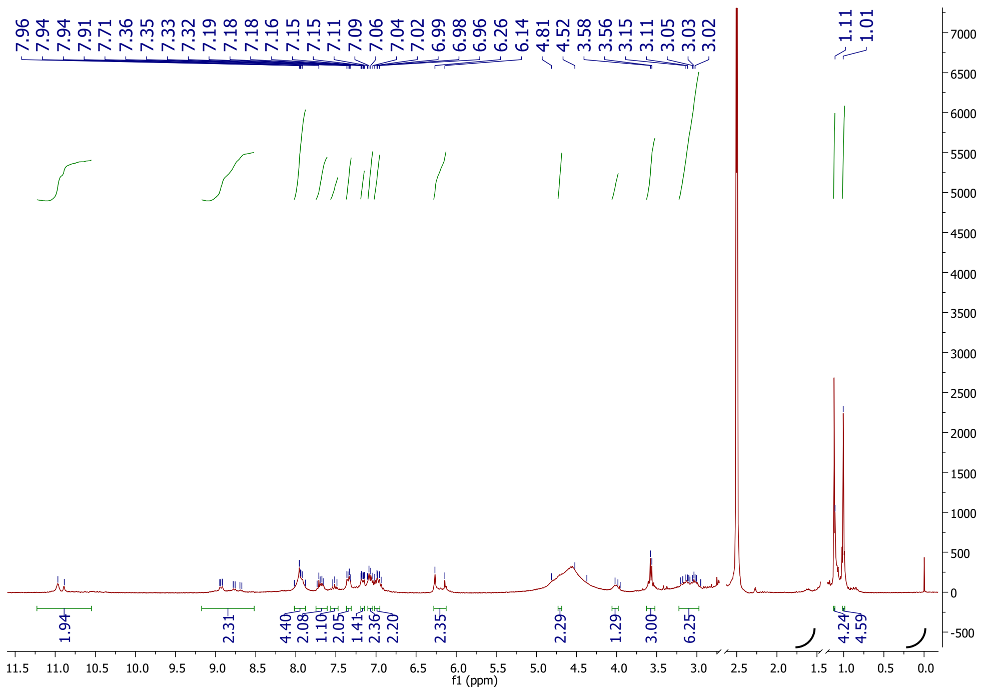

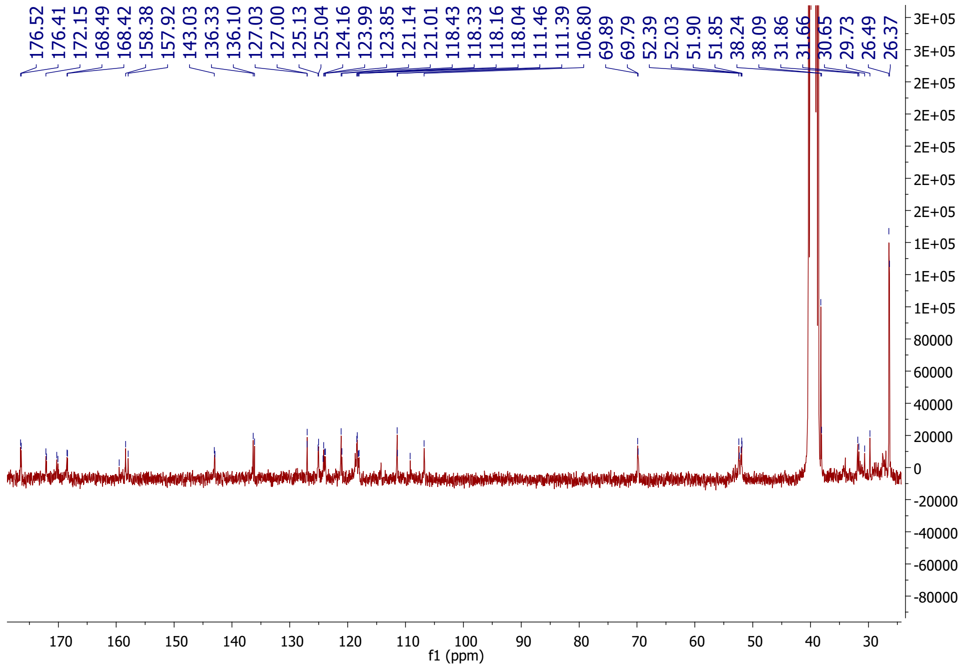

Figure S10. ^{1}H NMR (300 MHz, DMSO-*d*6) and ^{13}C NMR (75 MHz, DMSO-*d*6) spectra of compound **10**.

International Journal of
Molecular Sciences

MDPI

2. Physico-chemical properties of aqueous solutions

All compounds were dissolved in DMSO at $c=10^{-3}$ M. The stock solutions where stored at +4 °C. No visible precipitation in working solutions was noticed. The experiments were performed in buffer solution (sodium cacodylate buffer, I = 50 mM, pH = 7.0).

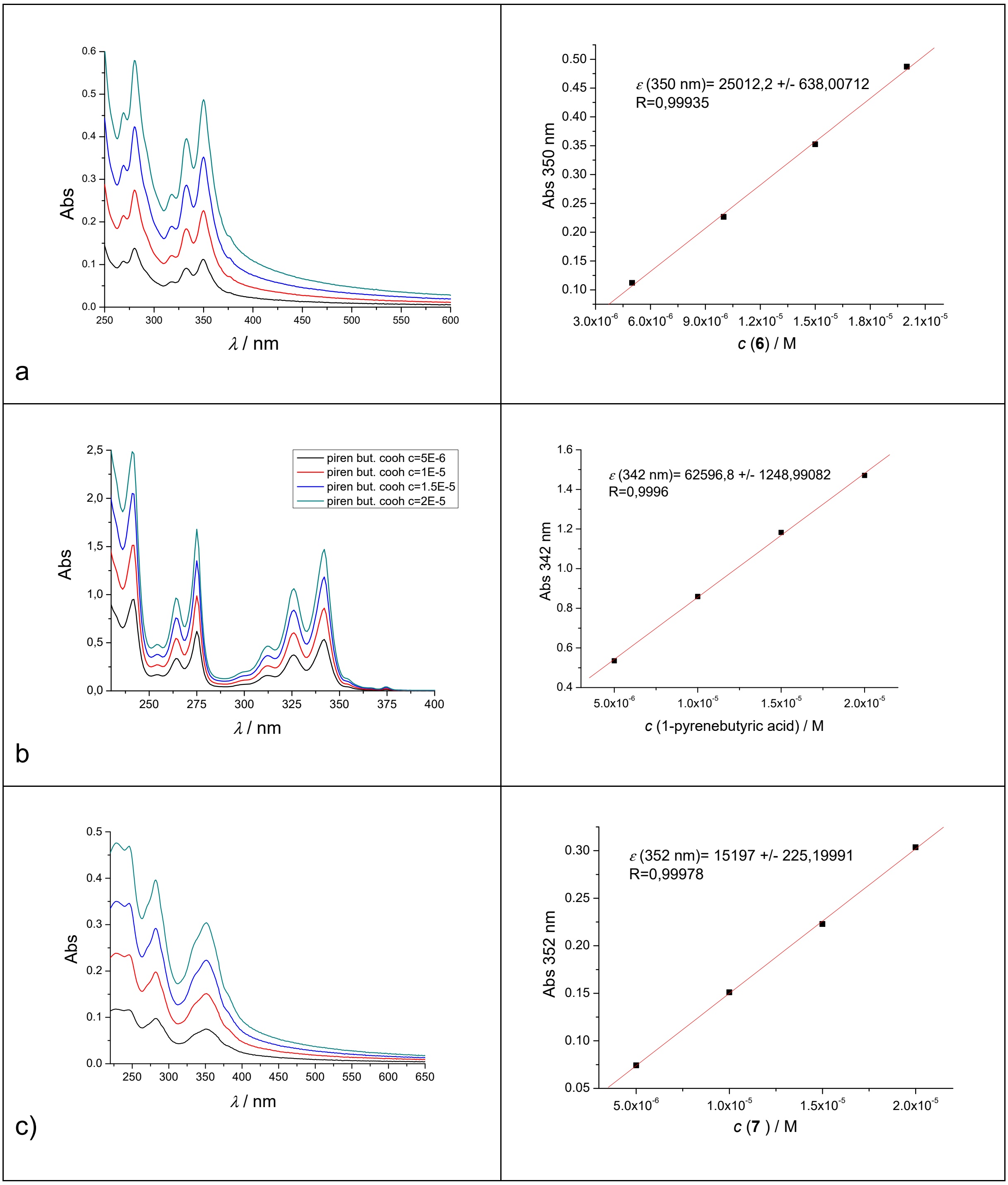

International Journal of
Molecular Sciences

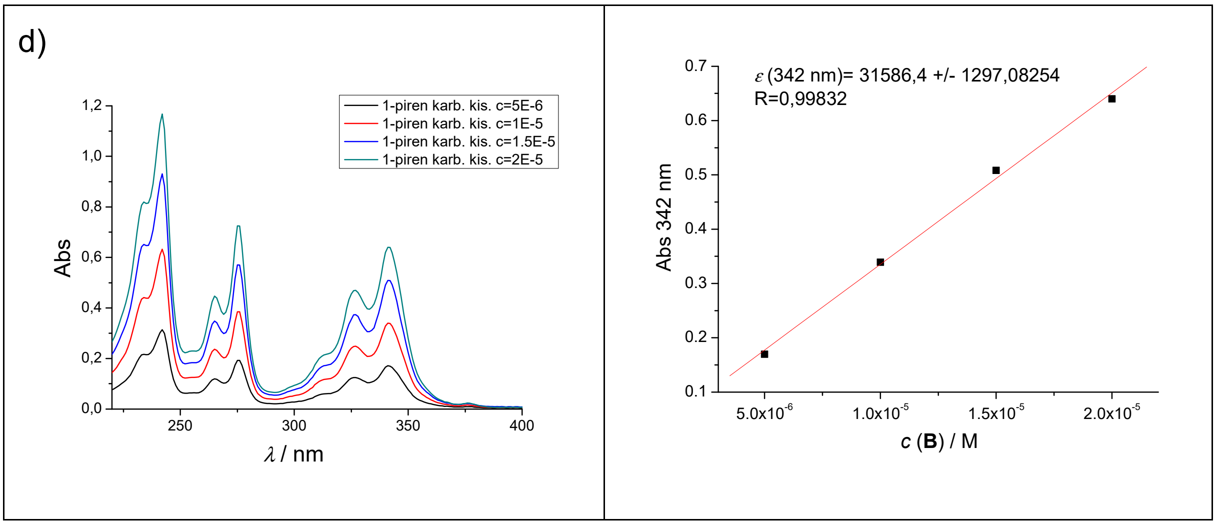

Figure S11. Concentration dependence of a) **6** and b) 1-pyrenebutyric acid **(A)** ; c) **7** and d) 1-pyrene carboxylic acid **(B)** in buffered solution pH 7, *I* = 0.05 M. in buffered solution pH 7, *I* = 0.05 M.

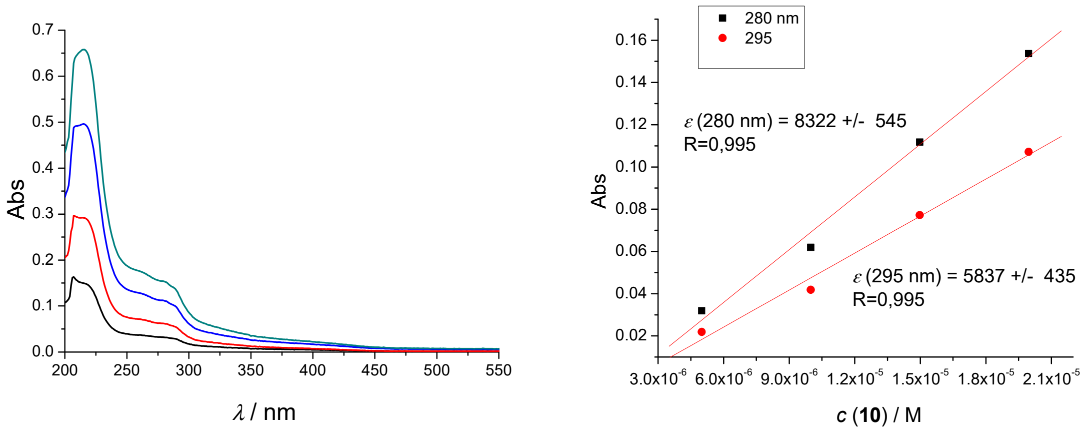

Figure S12. Concentration dependence (concentration range from 5×10^{-6} – 2×10^{-5} mol dm^{-3}) of **K14** in buffered solution pH 7, *I* = 0.05 mol dm^{-3}.

International Journal of
Molecular Sciences

MDPI

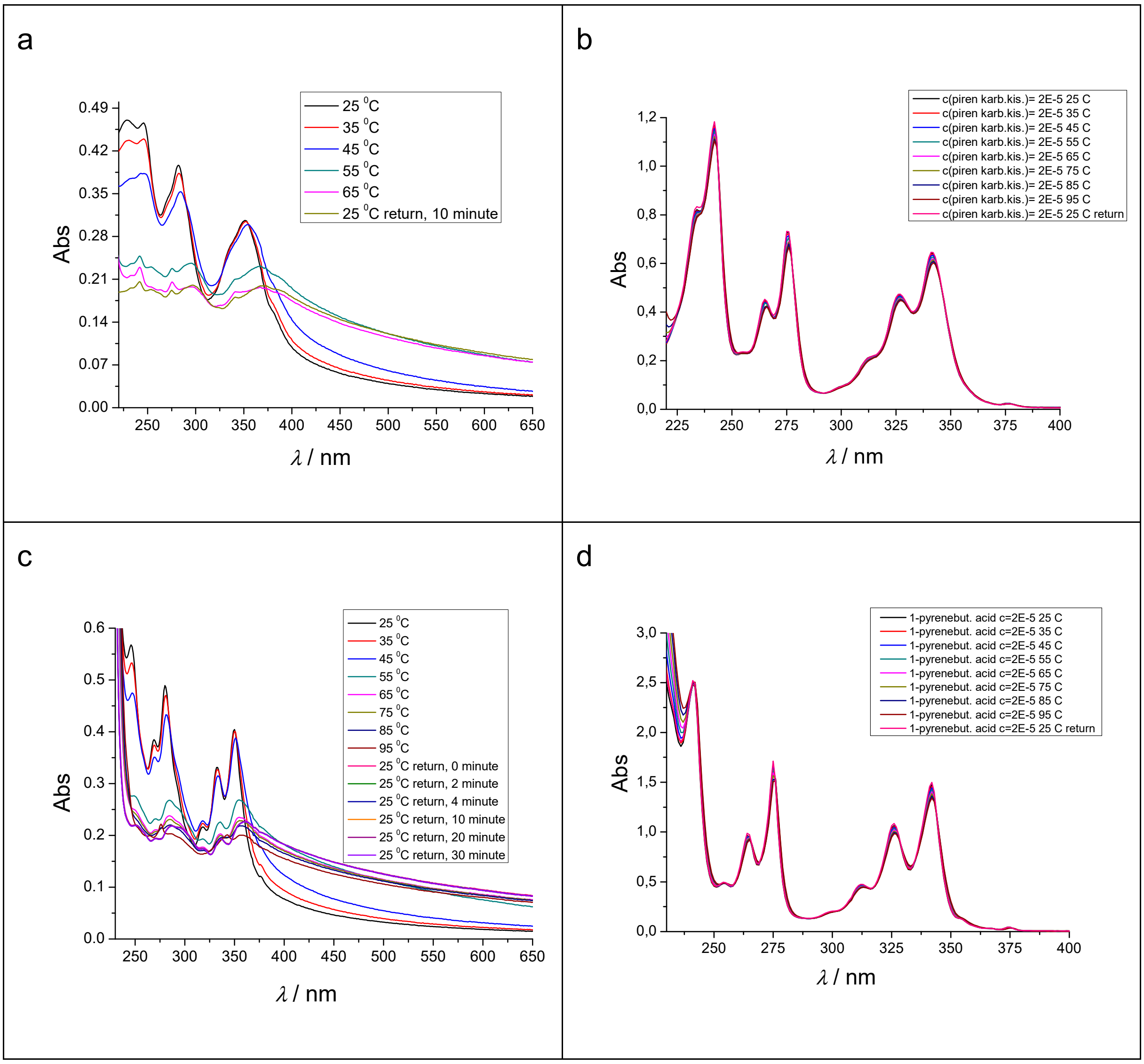

Figure S13. Temperature dependence of the UV/Vis spectra of a) **7,** b) 1-pyrenecarboxylic acid **(B),** c) **6** and d) 1-pyrenebutyric acid **(A)** (c=2 ×10^{-5} M) in buffered solution pH 7, *I* = 0.05 M.

International Journal of
Molecular Sciences

MDPI

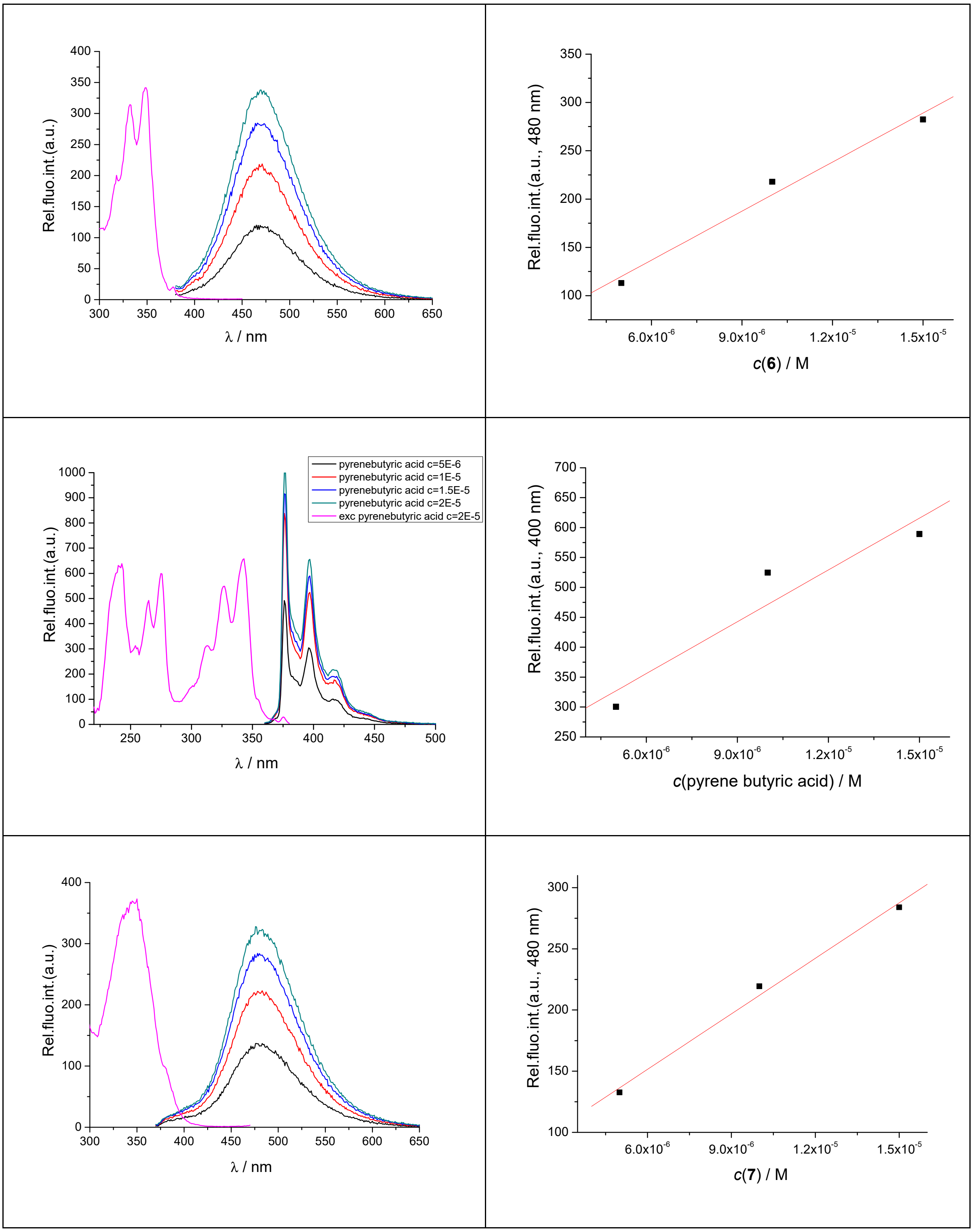

International Journal of
Molecular Sciences

MDPI

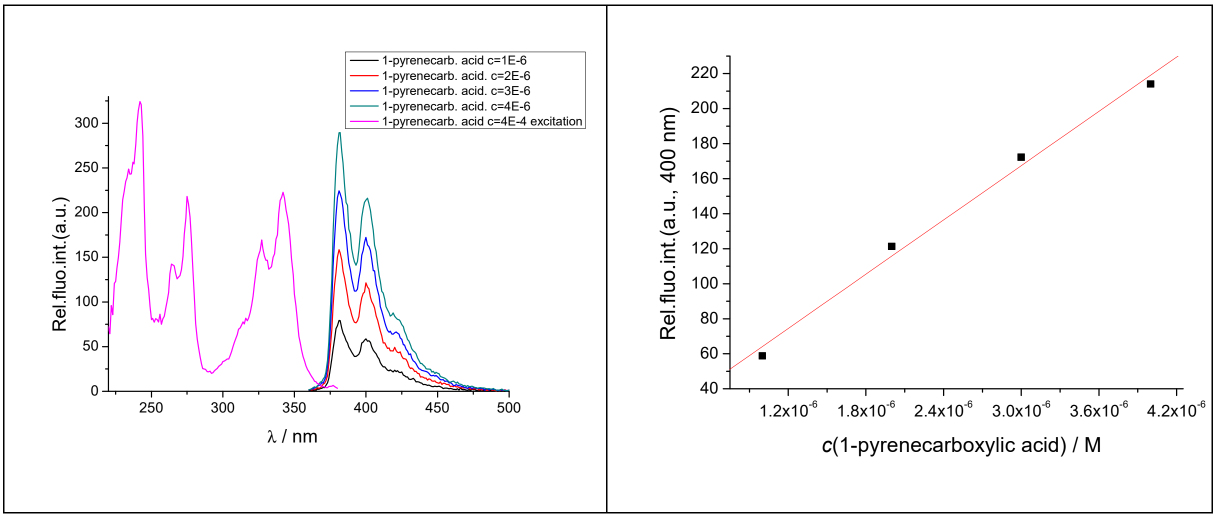

Figure S14. Fluorescence spectra of a) **6** (λ_{exc}= 350 nm) at concentration range from 5×10^{-6}– 1.5×10^{-5} M) and b) 1-pyrenebutyric acid **(A)** (λ_{exc}= 342 nm) at concentration range from (5×10^{-6}– 1.5×10^{-5} M), c) **7** (λ_{exc}= 352 nm) at concentration range from 5×10^{-6}– 1.5×10^{-5} M) and d) 1-pyrenecarboxylic acid **(B)** (λ_{exc}= 341 nm) at concentration range from (1×10^{-6}– 4×10^{-6} M), in buffer sodium cacodylate (pH 7.0, *I* = 0.05 M). Pink lines in the range 300 – 400 nm are excitation spectra collected at emission maximum.

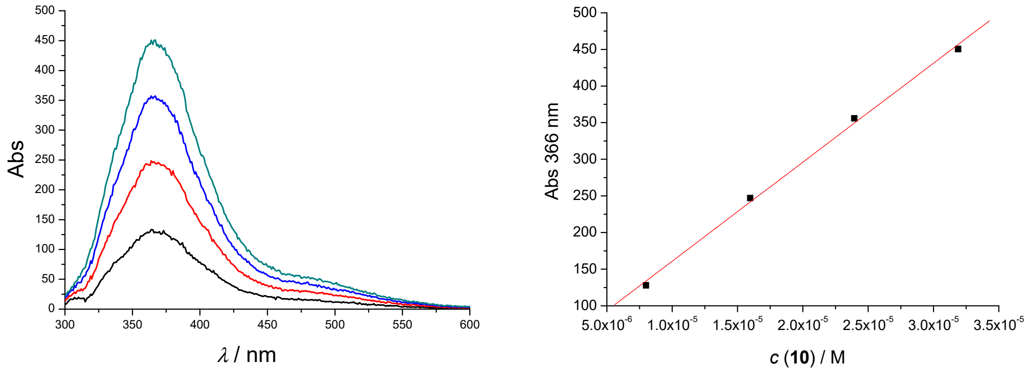

a b

Figure S15. a) Fluorescence spectra of **10** (λ_{exc}= 280 nm) at b) concentration range 2×10^{-6}– 8×10^{-6} M, in buffer sodium cacodylate (pH 7.0, *I* = 0.05 M).

International Journal of
Molecular Sciences

MDPI

Time Resolved Fluorescence, TCSPC (Time Correlated Single Photon Counting)

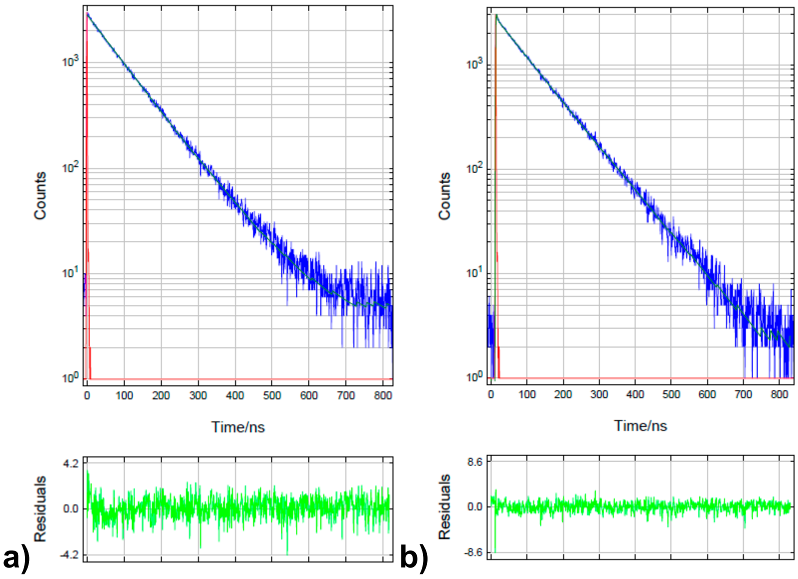

Figure S16. Data of fluorescence decay of **1-pyrenebutyric acid (A)** obtained by the Time Correlated Single-Photon Counting technique in **a)** non-degassed and **b)** degassed solution at λ_{exc} = 340 nm and λ_{em} = 419 nm. Done at pH 7.0, sodium cacodylate buffer, *I* = 0.05 M. Number of channels used in the fitting 1024 and 3000 cps. The instrument response function (IRF) was measured with an aqueous suspension of Silica Gel at the same conditions as decay. Fluorescence decay (lifetime), their relative representation and statistical parameter χ^2 are given in Table 1.

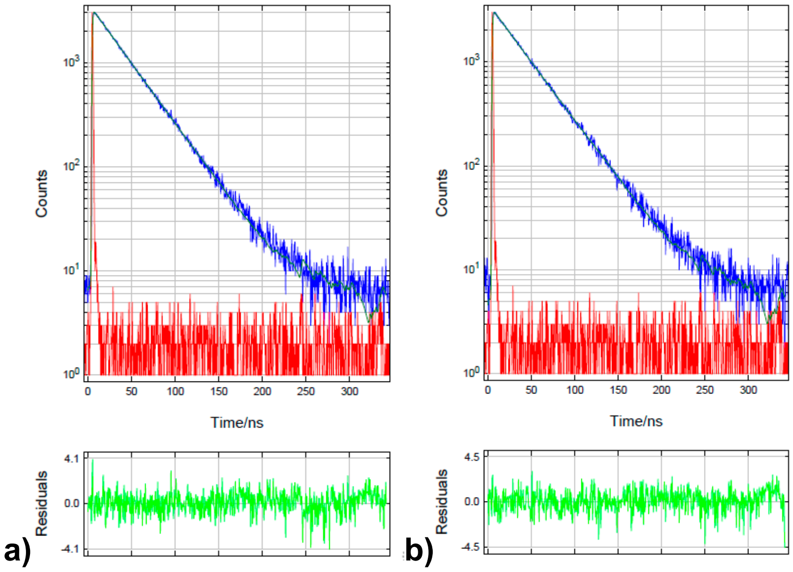

Figure S17. Data of fluorescence decay of **1-pyrenecarboxylic acid (B)** obtained by the Time Correlated Single-Photon Counting technique in **a)** non-degassed and **b)** degassed solution at λ_{exc} = 340 nm and λ_{em} = 400 nm at pH 7.0, sodium cacodylate buffer, *I* = 0.05 M. Number of channels used in the fitting 1024 and 3000 cps. The instrument response function (IRF) was measured with an aqueous suspension of Silica Gel at the same conditions as decay. Fluorescence decay (lifetime), their relative representation and statistical parameter χ^2 are given in Table 1.

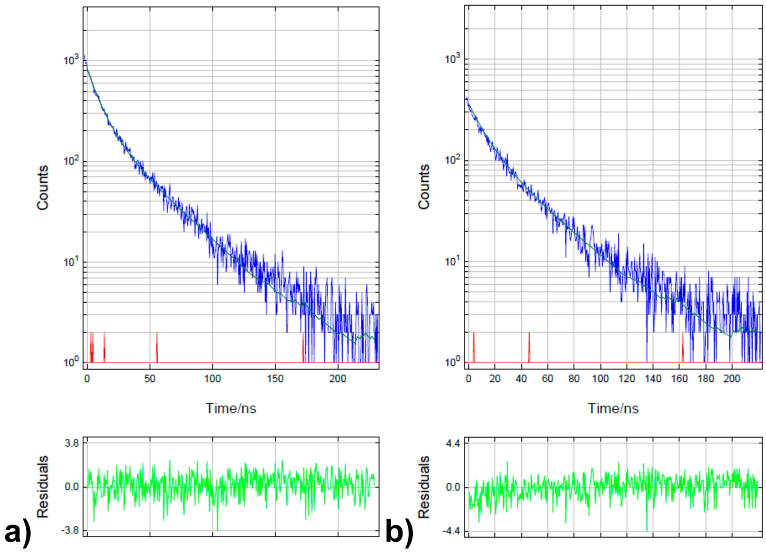

Figure S18. Data of fluorescence decay of **6** acid obtained by the Time Correlated Single-Photon Counting technique in **a)** non-degassed and **b)** degassed solution at λ_{exc} = 340 nm and λ_{em} = 471 nm at pH 7.0, sodium cacodylate buffer, *I* = 0.05 M. Number of channels used in the fitting 1024 and 3000 cps. The instrument response function (IRF) was measured with an aqueous suspension of Silica Gel at the same conditions as decay. Fluorescence decay (lifetime), their relative representation and statistical parameter χ^2 are given in Table 1.

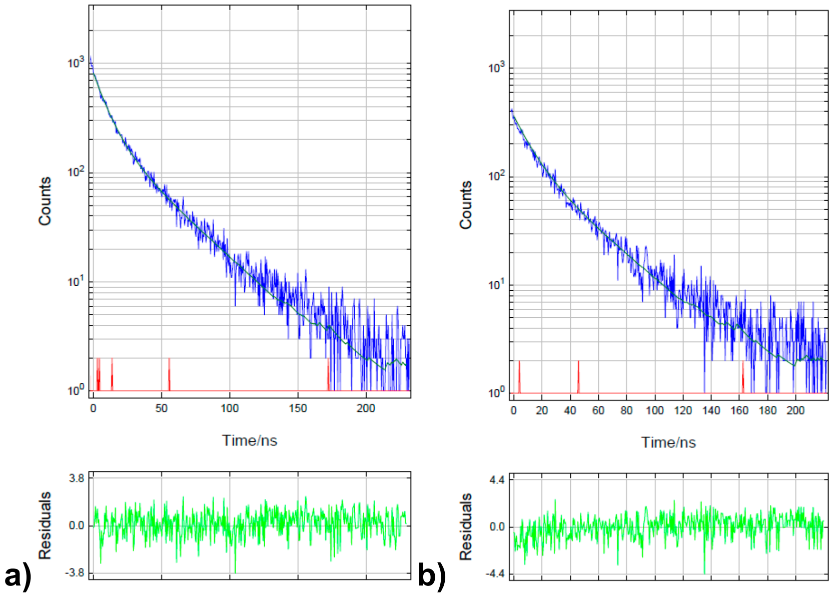

Figure S19. Data of fluorescence decay of **7** acid obtained by the Time Correlated Single-Photon Counting technique in **a)** non-degassed and **b)** degassed solution at λ_{exc} = 340 nm and λ_{em} = 472 nm at pH 7.0, sodium cacodylate buffer, *I* = 0.05 M. Number of channels used in the fitting 1024 and 3000 cps. The instrument response function (IRF) was measured with an aqueous suspension of Silica Gel at the same conditions as decay. Fluorescence decay (lifetime), their relative representation and statistical parameter χ^2 are given in Table 1.

International Journal of
Molecular Sciences

MDPI

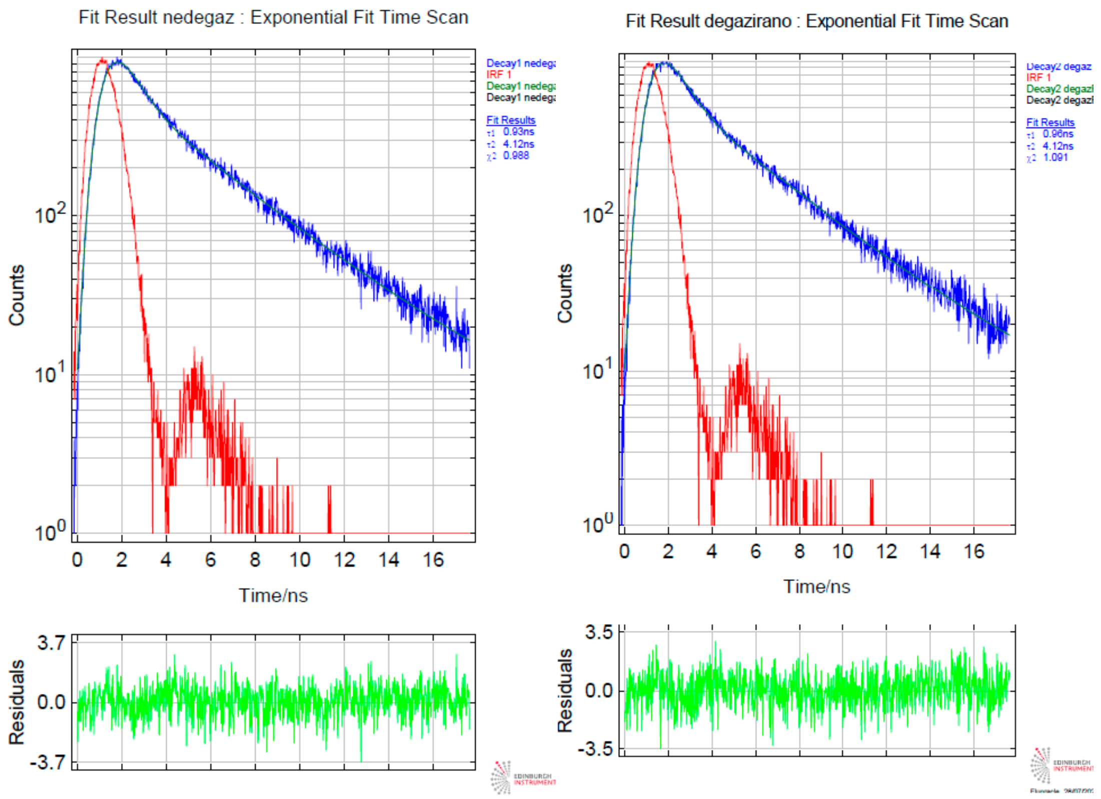

Figure S20. Data of fluorescence decay of **10** acid obtained by the Time Correlated Single-Photon Counting technique in **a)** non-degassed and **b)** degassed solution at λ_{exc} = 280 nm and λ_{em} = 380 nm at pH 7.0, sodium cacodylate buffer, *I* = 0.05 M. Number of channels used in the fitting 1024 and 3000 cps. The instrument response function (IRF) was measured with an aqueous suspension of Silica Gel at the same conditions as decay. Fluorescence decay (lifetime), their relative representation and statistical parameter χ^2 are given in Table 1.

Table S1. Lifetime data obtained by measuring lifetime decay of degassed **6** and **7**, and their complexes with ctDNA and with $CuCl_2$.

	**6**	**6 + ctDNA**	**6 + CuCl₂**
τ_1	2.682E-009 s	2.618E-009 s	2.241E-009 s
τ_2	1.034E-008 s	1.174E-008 s	9.496E-009 s
τ_3	3.548E-008 s	3.703E-008 s	3.374E-008 s
	**7**	**7 + ctDNA**	**7 + CuCl₂**
τ_1	2.541E-009 s	2.720E-009 s	2.774E-009 s
τ_2	1.311E-008 s	1.320E-008 s	1.327E-008 s
τ_3	3.693E-008 s	3.842E-008 s	3.587E-008 s

International Journal of *Molecular Sciences*

MDPI

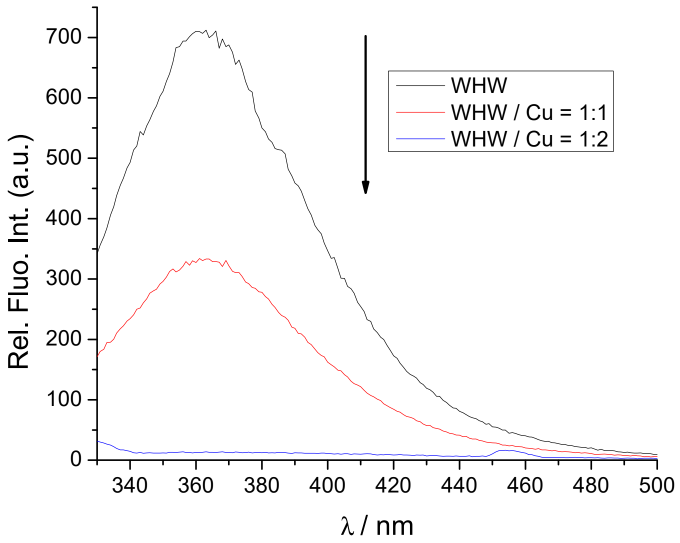

Figure S21. Fluorimetric titration of **WHW** (c = 5×10^{-6} M ; λ_{exc}= 295 nm) with $CuCl_2$. Done at pH = 7.0, sodium cacodylate buffer, I = 0.05 M.

3. Study of interactions with double-stranded DNA/RNA

<u>Structural properties of studied DNA and RNA</u>

Polynucleotides were purchased as noted: poly dGdC – poly dGdC, poly dAdT – poly dAdT, poly A – poly U, *calf thymus* (ct)-DNA (Aldrich) and dissolved in sodium cacodylate buffer, *I* = 0.05 M, pH=7.0. The ct-DNA was additionally sonicated and filtered through a 0.45 mm filter to obtain mostly short (ca. 100 base pairs) rod-like B-helical DNA fragments [[7]]. The polynucleotide concentration was determined spectroscopically [[8]] as the concentration of phosphates (corresponds to *c*(nucleobase)).

Table S2. Groove widths and depths for selected nucleic acid conformation [9,10].

Structure type	Groove width [Å]		Groove depth [Å]	
	major	minor	major	minor
[a] poly rA – poly rU (AU-RNA)	**3.8**	10.9	**13.5**	2.8
[b] ct-DNA (48% of GC-pairs)	11.4	3.3	7.5	7.9
[b] poly dAdT – poly dAdT(AT-DNA)	11.2	**6.3**	8.5	**7.5**
[c] poly dGdC – poly dGdC	13.5	9.5	10.0	7.2

[a] A - helical structure
[b] B - helical structure
[c] B- helical structure with minor groove sterically blocked by amino groups of guanines

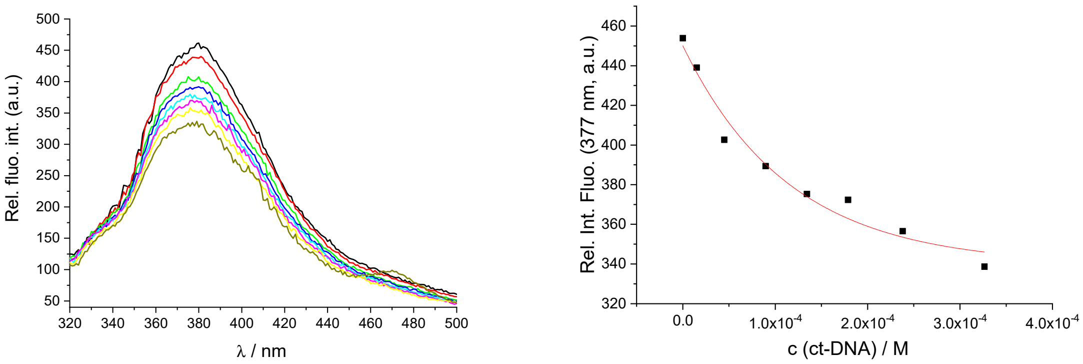

a)

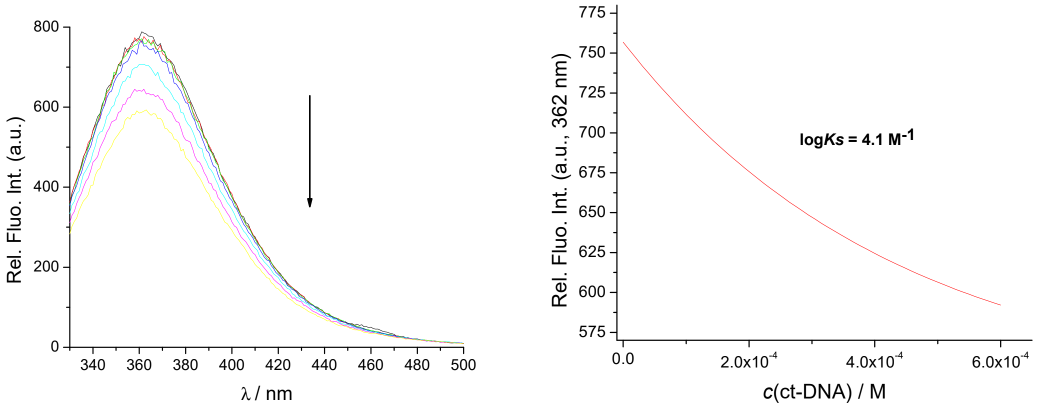

b)

Figure S22. a) Fluorimetric titration of **10** (c = 5×10^{-6} M ; λ_{exc}= 295 nm) with ctDNA. RIGHT: dependence of fluorescence at λ_{max}= 377 nm on c(ctDNA). b) Fluorimetric titration of **WHW** (c = 5×10^{-6} M ; λ_{exc}= 295 nm) with ctDNA. RIGHT: dependence of fluorescence at λ_{max}= 362 nm on c(ctDNA). Done at pH 7, sodium cacodylate buffer, I = 0.05 M. Red line: fitting titration data to Scatchard eq.; n=0.2.

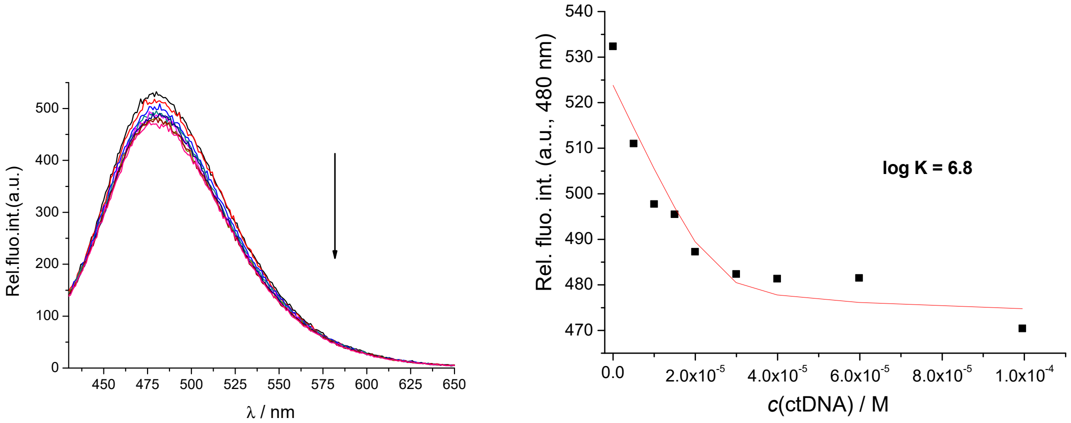

a)

International Journal of *Molecular Sciences*

MDPI

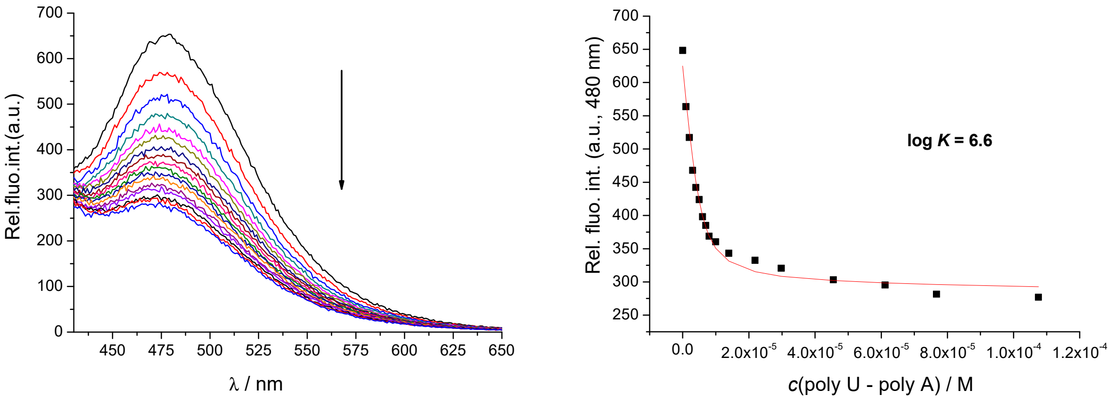

b)

Figure S23. Fluorimetric titration of **7** (c = 5×10^{-6} M ; λ_{exc}= 352 nm) a) with ctDNA. RIGHT: dependence of fluorescence at λ_{max}= 480 nm on *c*(ctDNA). b) with poly A – poly U. RIGHT: dependence of fluorescence at λ_{max}= 480 nm on *c*(pApU). Done at pH 7, sodium cacodylate buffer, *I* = 0.05 M.

International Journal of
Molecular Sciences

MDPI

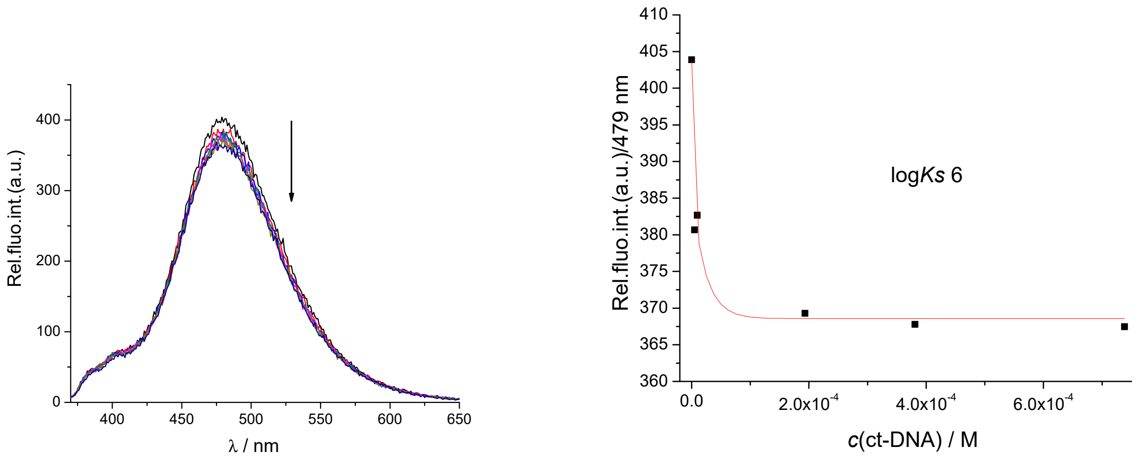

Figure S24. Fluorimetric titration of **7**/Cu^{2+} complex (c(**7**)= 5×10^{-6} M ; λ_{exc}= 352 nm) with ctDNA. RIGHT: dependence of fluorescence at λ_{max}= 479 nm on c(ctDNA). Done at pH 7, sodium cacodylate buffer, I = 0.05 M.

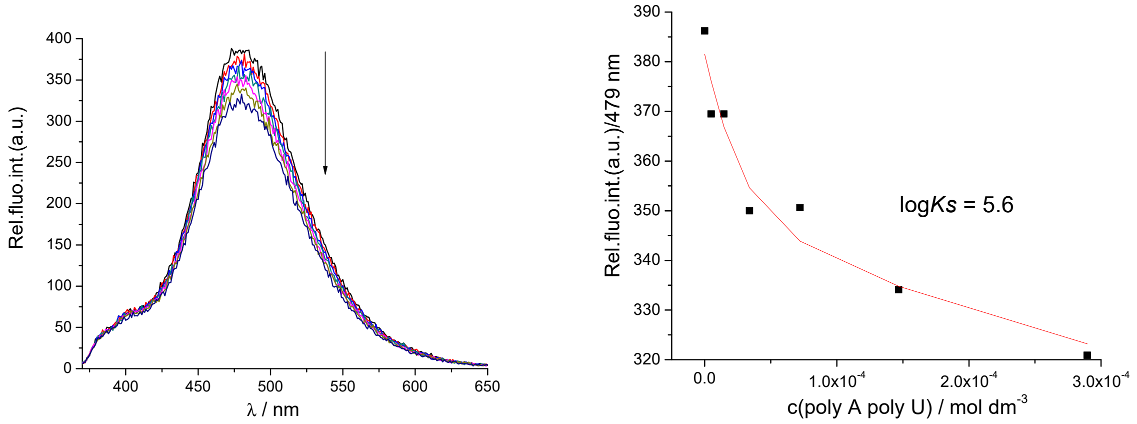

Figure S25. Fluorimetric titration of complex **7**/Cu^{2+} complex (c(**7**)= 5×10^{-6} M ; λ_{exc}= 352 nm) with poly A poly U. RIGHT: dependence of fluorescence at λ_{max}= 479 nm on c(poly A poly U). Done at pH 7, sodium cacodylate buffer, I = 0.05 M.

International Journal of
Molecular Sciences

MDPI

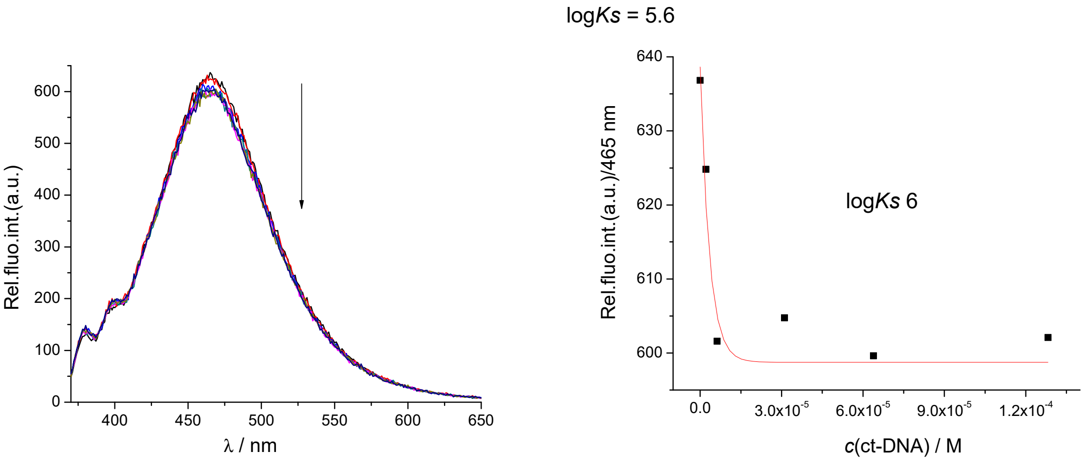

Figure S26. Fluorimetric titration of **6**/Cu^{2+} complex (c(**6**)= 5×10^{-6} M ; λ_{exc}= 352 nm) with ct-DNA. RIGHT: dependence of fluorescence at λ_{max}= 465 nm on c(ctDNA). Done at pH 7, sodium cacodylate buffer, I = 0.05 M.

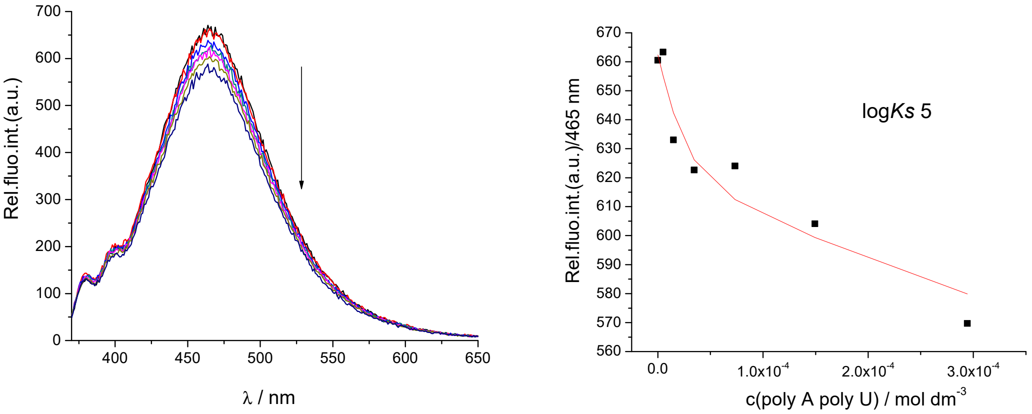

Figure S27. Fluorimetric titration of **6**/Cu^{2+} complex (c(**6**)= 5×10^{-6} M ; λ_{exc}= 352 nm) with poly A poly U. RIGHT: dependence of fluorescence at λ_{max}= 465 nm on c(poly A poly U). Done at pH 7, sodium cacodylate buffer, I = 0.05 M.

CD experiments

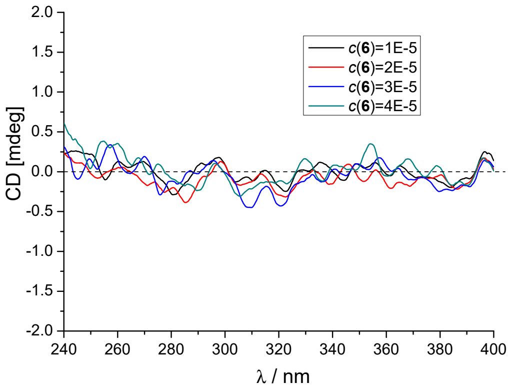

a)

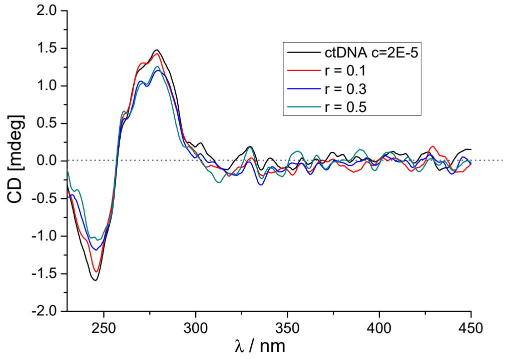

b)

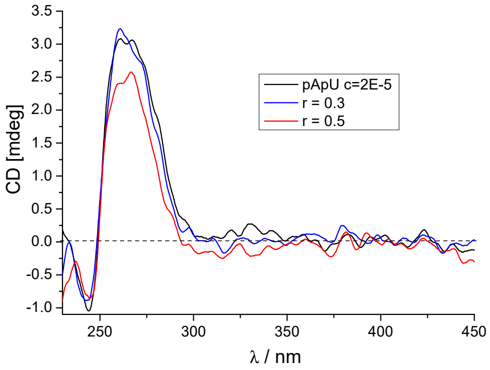

c)

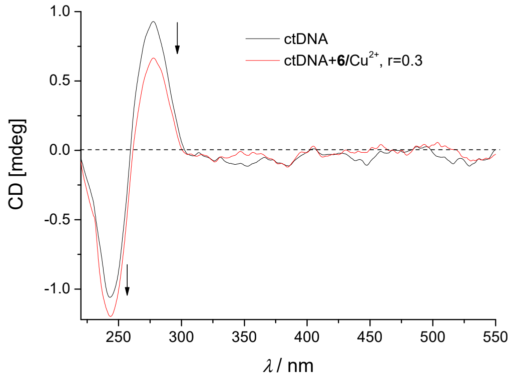

d)

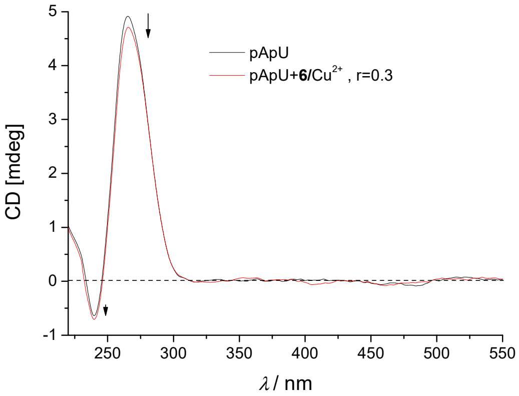

e)

Figure S28. CD spectra of: a) **6** $c = 1\times10^{-5}$– 4×10^{-5} M. The CD titrations with **6** of b) ct-DNA and c) poly A – poly U at molar ratios r = [**6**] / [polynucleotide]; the CD titrations with **6**/Cu^{2+} of d) ctDNA and e) poly A – poly U at r = [**6**/Cu^{2+}] / [polynucleotide] =0.3. Done at pH 7.0, buffer sodium cacodylate, I = 0.05 M.

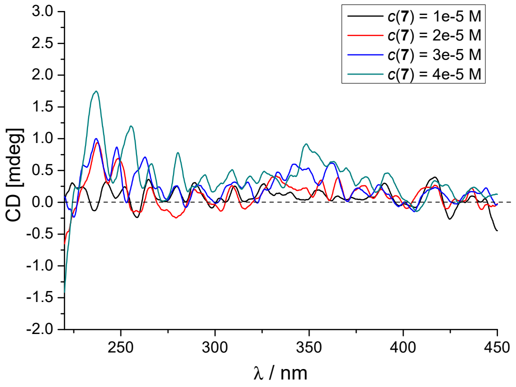

a)

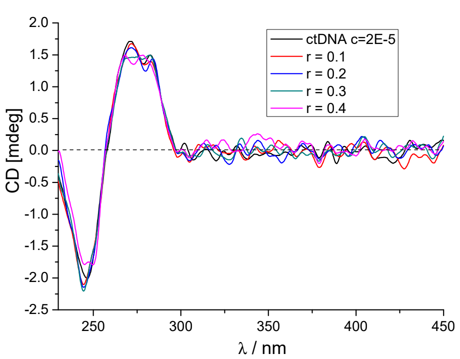

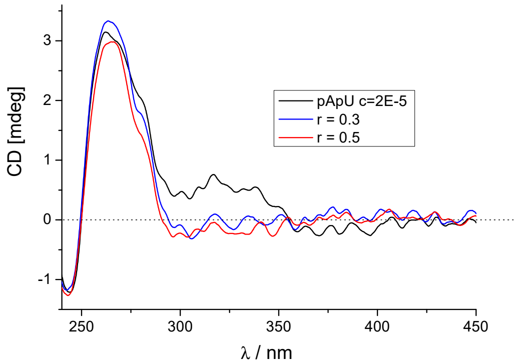

b) c)

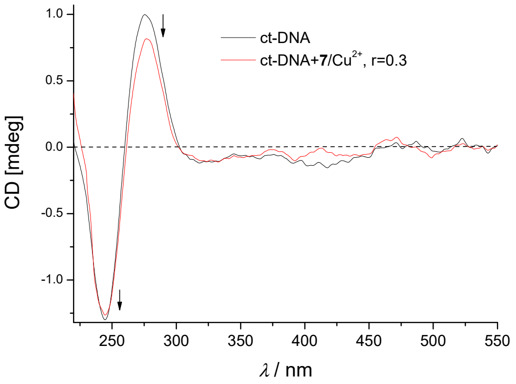

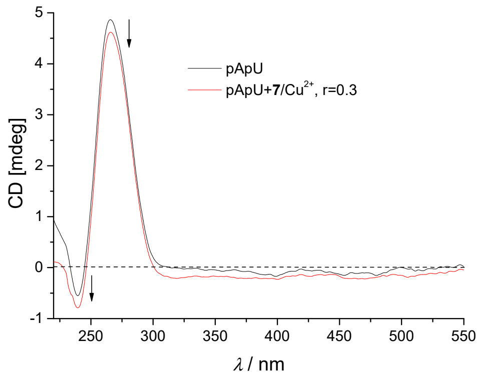

d) e)

Figure S29. CD spectra of: a) **7** *c* = 1×10^{-5}– 4×10^{-5} M. The CD titrations with **7** of b) ct-DNA and c) poly A – poly U at molar ratios *r* = [**7**] / [polynucleotide]; the CD titrations with **7**/Cu^{2+} of d) ctDNA and e) poly A – poly U at *r* = [**7**/Cu^{2+}] / [polynucleotide] =0.3. Done at pH 7.0, buffer sodium cacodylate, *I* = 0.05 M.

International Journal of
Molecular Sciences
MDPI

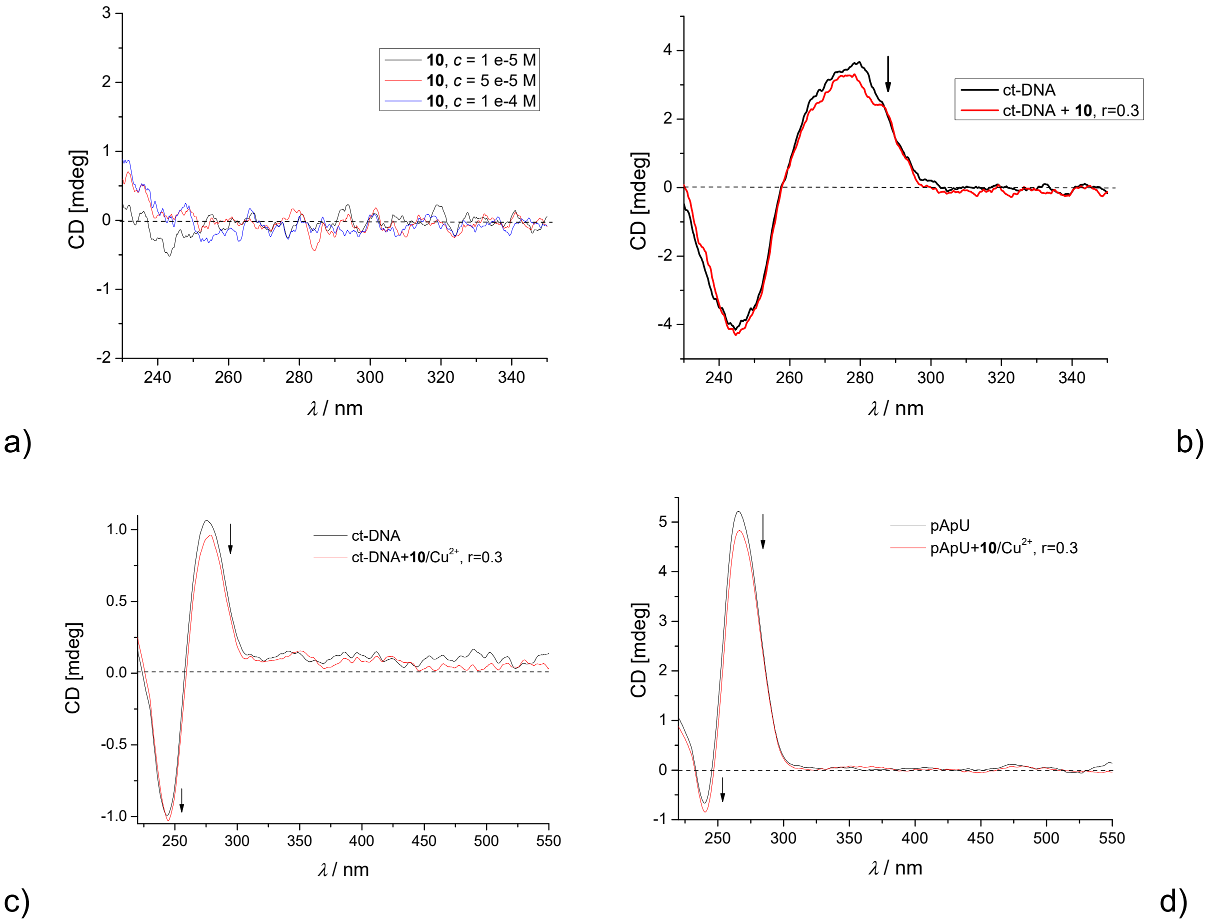

Figure S30. CD spectra of: a) **10** $c = 1\times10^{-5} – 1\times10^{-4}$ M. CD titrations with **10** of b) ct-DNA at molar ratio r = [**10**] / [polynucleotide] = 0.3; CD titrations with **10**/Cu^{2+} of c) ct-DNA and d) poly A – poly U at r = [**10**/Cu^{2+}] / [polynucleotide] =0.3. Done at pH 7.0, buffer sodium cacodylate, I = 0.05 M.

International Journal of
Molecular Sciences

MDPI

Thermal denaturation of DNA/RNA

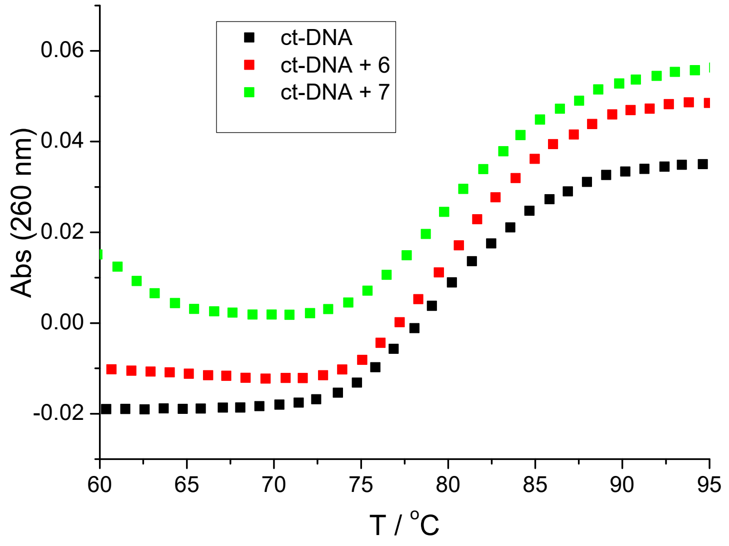

Figure S31. Thermal denaturation curves of ct-DNA (c(ct-DNA) = 2.5 × 10^{-5} M, $r_{[\mathbf{(peptide)}]/[\text{ct-DNA}]}$ = 0.3) at pH 7.0 (sodium cacodylate buffer, I = 0.05 M) upon addition of **6** or **7**. Error in ΔTm values: ±0.5 °C.

International Journal of
Molecular Sciences
MDPI

Complexes of **6** and **7** with $CuCl_2$ were prepared by adding 5-fold excess of $CuCl_2$ to the 5×10^{-6}M of peptide in the 1 mL of the sodium cacodylate buffer (pH 7). After 1 min. incubation period (to ensure complex formation), aliquot of DNA or RNA was added and immediately thermal denaturation experiment performed.

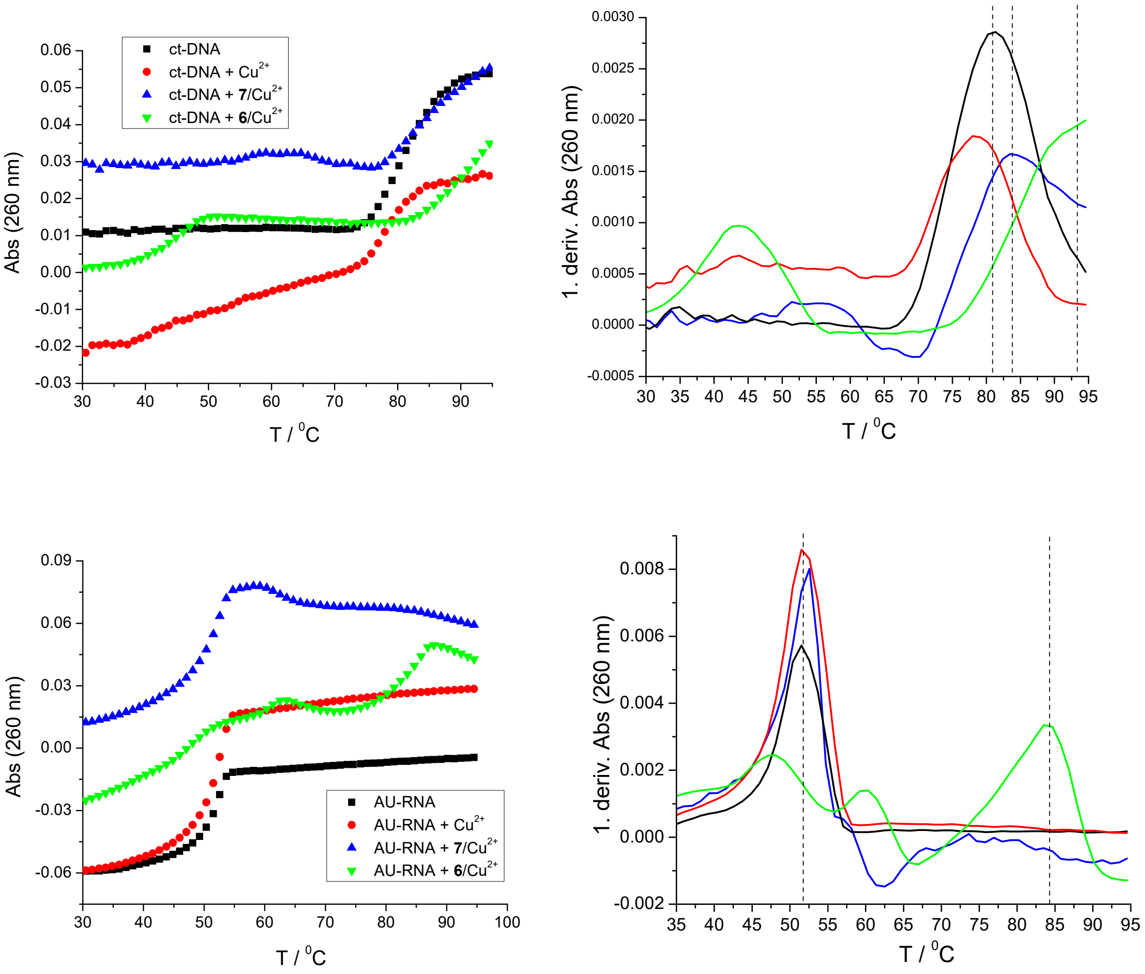

Figure S32. Thermal denaturation curves of: UP) ct-DNA (c(ct-DNA) = 2.5×10^{-5}M, and DOWN) poly A poly U (c(RNA)= 2.5 10^{-5}M, at pH 7.0 (sodium cacodylate buffer, I = 0.05 M) upon addition of only Cu^{2+} solution, **6**/Cu^{2+} or **7**/Cu^{2+} complex at ratio r[**6**/Cu^{2+} or **7**/Cu^{2+}]/[polynucleotide] = 0.25). Error in ΔTm values: ±0.5 °C.

International Journal of
Molecular Sciences

MDPI

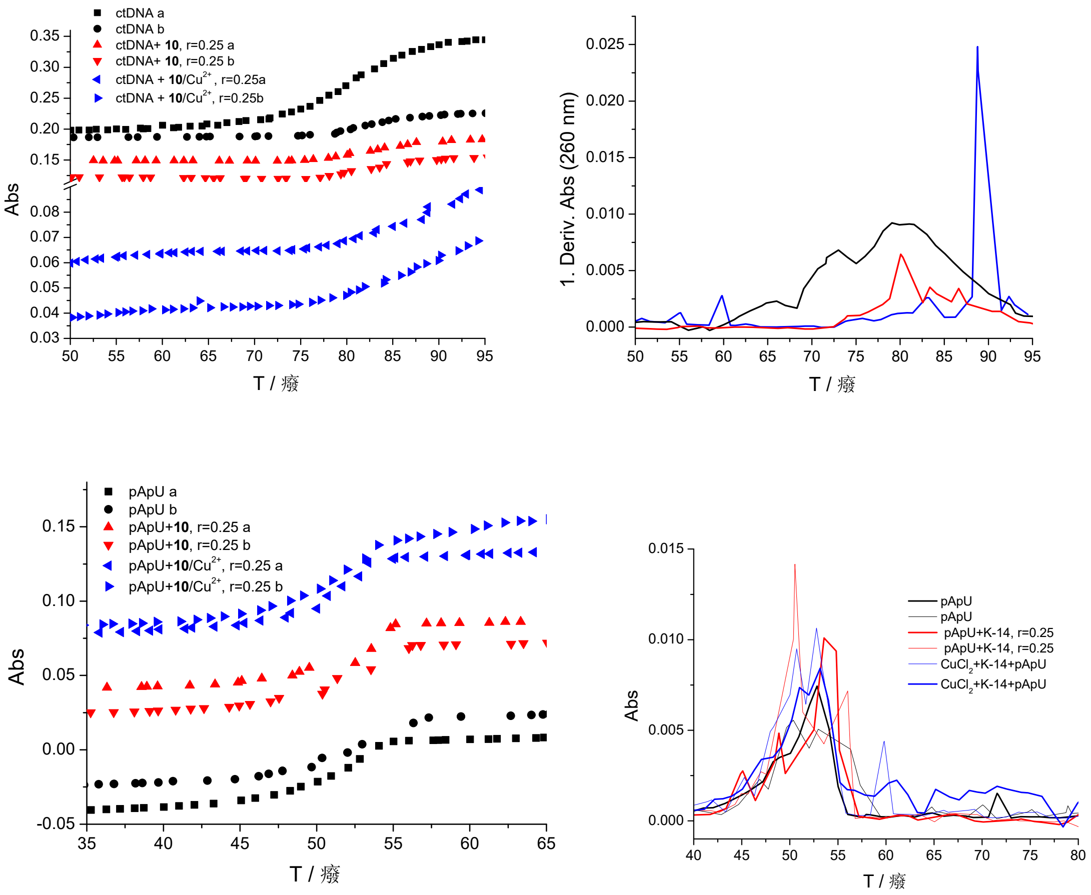

Figure S33. Thermal denaturation curves of: UP) ct-DNA (c(ct-DNA) = 2.5×10^{-5}M, and DOWN) poly A poly U (c(RNA)= 2.5 10^{-5}M, at pH 7.0 (sodium cacodylate buffer, I = 0.05 M) upon addition of **10** or **10**/Cu^{2+} at ratio r[**10**]/[polynucleotide] = 0.25). Error in ΔTm values: ±0.5 °C.

DAPI displacement:

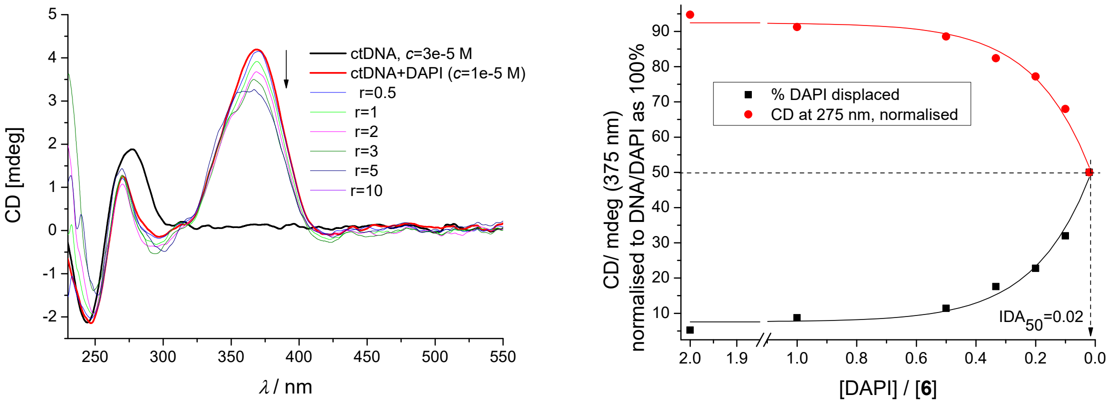

a)

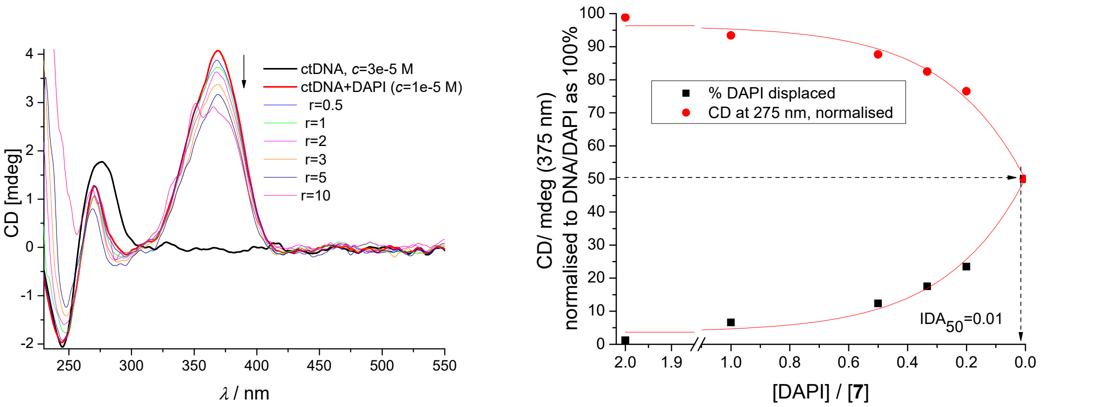

b)

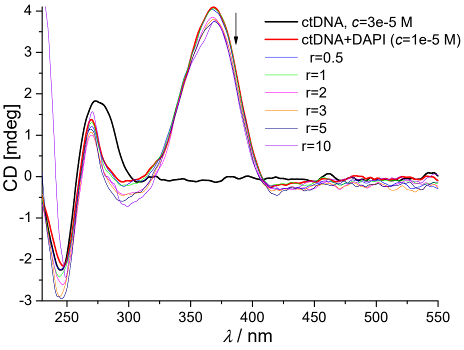

c)

Figure S34. Changes in induced CD band of DAPI at 375 nm (c = 5 × 10^{-6} M) from the complex with the ct-DNA (c = 2 × 10−5 M): a) upon addition of a) **6**,b) **7** and c) **10**. Done at pH 7.0, buffer sodium cacodylate, I = 0.05 M. The percentage of DAPI displaced by peptidoid was calculated, given IDA50% values presenting the ratio r = [DAPI] / [peptidoid] at which 50% of ICD375nm band is decreased in respect to the zero baseline of the free DNA.

International Journal of Molecular Sciences

4. Biology:

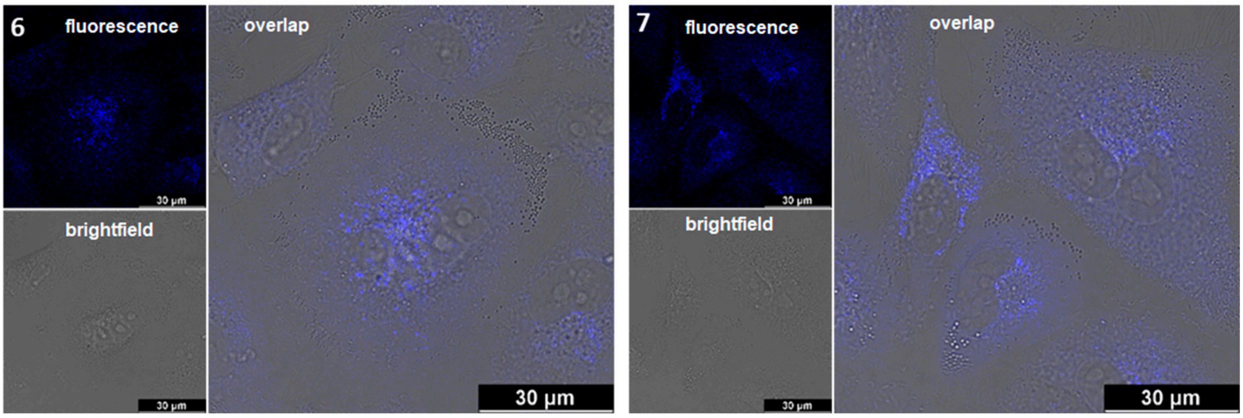

Figure S35. Uptake of compounds **6** and **7** in live A549 cells. Cells were treated with 10 µM of each compound respectively for 90 min at 37ºC and subsequently monitored by confocal microscopy (ʎexc = 405 nm, ʎem = 450-550 nm).

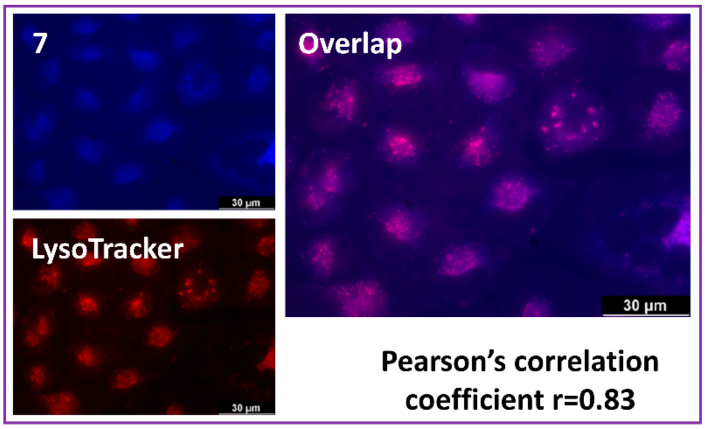

Figure S36. Intracellular localization of **7** (shown in blue) in A549 cells. Cells were treated with 10 µM of compound for 90 min at 37ºC and co-localization with lysosomes (LysoTracker), shown in red, was monitored by confocal microscopy. Cells were treated with 10 µM of each compound respectively for 90 min at 37ºC and subsequently monitored by confocal microscopy (ʎexc = 405 nm, ʎem = 450-550 nm). Analysis was done using ImageJ software and the appropriate JACoP plugin. Co-localization was assessed by the Pearson correlation coefficient.

5. References:

1. Stanley, N.J.; Sejer Pedersen, D.; Nielsen, B.; Kvist, T.; Mathiesen, J.M.; Bräuner-Osborne, H.; Taylor, D.K. ; Abell, A.D. 1,2,3-Triazolyl amino acids as AMPA receptor ligands. *Bioorg. Med. Chem. Lett*. **2010**, *20*, 7512–7515.
2. Li, J.; Sha, Y. A Convenient Synthesis of Amino Acid Methyl Esters. *Molecules* **2008**, *13*, 1111–1119, https://doi.org/10.3390/molecules13051111.
3. Dourtoglou, V.; Gross, B.; Lambropoulou, V.; Zioudrou, C. O-benzotriazolyl-N,N,N',N'-tetramethyluronium hexafluoro-phosphate as coupling reagent for the synthesis of peptides of biological interest. *Synthesis* **1984**, *07*, 572–574.
4. Feldman, A.K.; Colasson, B.; Fokin, V.V. One-Pot Synthesis of 1,4-Disubstituted 1,2,3-Triazoles from In Situ Generated Azides. *Org. Lett.* **2004**, *6*, 3897–3899, https://doi.org/10.1021/ol048859z.
5. Hassan, S.; Mueller, T.J.J. ChemInform Abstract: Multicomponent Syntheses Based upon Copper-Catalyzed Alkyne-Azide Cycloaddition. *ChemInform* **2015**, *46*, https://doi.org/10.1002/chin.201521260.
6. Keller, O.; Keller, W.E.; van Look, G.; Wersin, G. Working with Hazardous Chemicals. *Org. Synth*. **1985**, *63*, 160.
7. Chaires, J.; Dattagupta, N.; Crothers, D.M. Studies on interaction of anthracycline antibiotics and deoxyribonucleic acid: equilibrium binding studies on the interaction of daunomycin with deoxyribonucleic acid. *Biochemistry* **1982**, *21*, 3933–3940, https://doi.org/10.1021/bi00260a005.
8. Tumir, L.-M.; Piantanida, I.; Cindrić, I.J.; Hrenar, T.; Meić, Z.; Žinić, M. New permanently charged phenanthridinium-nucleobase conjugates. Interactions with nucleotides and polynucleotides and recognition of ds-polyAH+. *J. Phys. Org. Chem*. **2003**, *16*, 891–899, https://doi.org/10.1002/poc.680.
9. Saenger, W. *Principles of Nucleic Acid Structure*; Springer: New York, NY, USA, 1983.
10. Cantor, C.R.; Schimmel, P.R. *Biophysical Chemistry*; WH Freeman and Co**.:** San Francisco, CA, USA, 1980; Volume 3, pp. 1109-1181.
